# Catalyst-Free Site Selective Hydroxyalkylation of 5-Phenylthiophen-2-amine with α-Trifluoromethyl Ketones through Electrophilic Aromatic Substitution

**DOI:** 10.3390/molecules27030925

**Published:** 2022-01-29

**Authors:** Valentin Duvauchelle, David Bénimélis, Patrick Meffre, Zohra Benfodda

**Affiliations:** UPR CHROME, Université de Nîmes, CEDEX 1, F-30021 Nîmes, France; valentin.duvauchelle@unimes.fr (V.D.); david.benimelis@unimes.fr (D.B.); patrick.meffre@unimes.fr (P.M.)

**Keywords:** hydroxyalkylation, trifluoromethyl hydroxyalkylation, trifluoromethyl ketone, catalyst-free, site selective, chemoselective, 2-aminothiophene, 5-phenylthiophen-2-amine

## Abstract

An original and effective approach for achieving trifluoromethyl hydroxyalkylation of 5-phenylthiophen-2-amine using α-trifluoromethyl ketones is described. In the last few years, reaction of Friedel-Crafts had been widely used to realize hydroxyalkylation on heterocycles such as indoles or thiophenes by means of Lewis acid as catalyst. Additionally, amine functions are rarely free when carbonyl reagents are used because of their tendency to form imines. This is the first time that a site-selective electrophilic aromatic substitution on C_3_ atom of an unprotected 5-phenylthiophen-2-amine moiety is reported. The liberty to allow reaction in neutral conditions between free amine is valuable in a synthesis pathway. The reaction proceeds smoothly using an atom-economical metal-and catalyst-free methodology in good to excellent yields. A mechanism similar to an electrophilic aromatic substitution has been proposed.

## 1. Introduction

**Figure 1:**
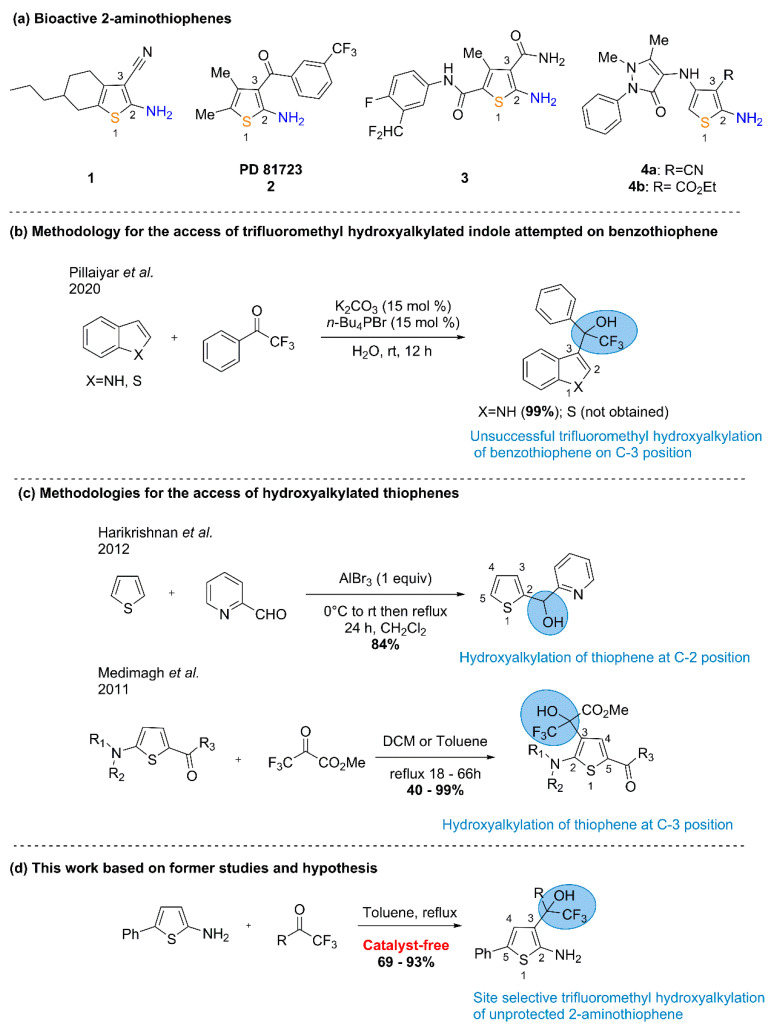
Background and overview. (**a**) Bioactives 2-AT derivatives. (**b**) Method to access to trifluoromethyl hydroxyalkylated indole. (**c**) Methods to access to hydroxyalkylated thiophenes. (**d**) First hypothesis based on previous studies.

2-aminothiophene (2-AT) moiety is widespread in FDA-approved drugs and is a privileged scaffold in medicinal chemistry that is known to confer many biological activities [1,2,3,4,5]. As examples, substituted-2-AT moiety such as compound **1** (Figure 1a) demonstrated activity against *Mycobacterium tuberculosis* by targeting the Ag85 enzymes [3]. **PD 81723** (**2**, Figure 1a) has been shown to be the first allosteric specific and selective adenosine A_1_ receptor ligands [6]. Compound **3** has been described as a hepatitis B virus replication inhibitor [7] and compound **4** showed antimicrobial activity against *A. fumigatus*, *G. candidum, C. albicans* and *S. racemosum* [8]. 2-AT derivatives are mostly synthesized using Gewald reaction [9,10]. Technically, it involves condensation of a carbonyl derivative, a α-cyanoester in the presence of sulfur source. To this day, used methodologies often undergo to the generation of trisubstituted thiophene ring with an electron withdrawing group–particularly negative mesomeric effect–on C_3_ atom (Figure 1a) [11,12,13,14,15].

Over recent decades, interest for hydroxyalkylation on aryl derivatives as C-C bond forming reaction has grown [16,17,18,19]. Ullyot first reported hydroxyalkylation of aryl compounds with a carbonyl derivative under acidic conditions as new way to synthesize benzoins [20]. Thereafter, synthesis methodologies have been refined to fit with chemical diversity: heteroaryls were used as substrates; carbonyl derivatives were more complex, likewise the Lewis acids. The methodologies described, respectively, by Schnakenburg [21], Ramanathan [22] and Chatti [23] are relevant examples of hydroxyalkylation on heteroaryls scaffolds (Figure 1b,c).

Hence, trifluoromethyl group introduction onto 2-AT via hydroxyalkylation methodology provides a dual benefit: it introduces a chemical diversity that was lacking in 2-ATs and it inserts the trifluoromethyl group, which is very interesting from a medicinal point of view because it is known to confer biological properties of high value [24,25,26,27]. Chemists are currently faced with the difficult task of increasing the efficacy of this kind of reactions while also seeking greener processes [28]. As we know, a more responsible use of metals as catalysts as well as the use of atom economical reactions participate in the development of a greener chemistry [29,30,31]. To our knowledge, the site-selective, metal-and catalyst-free trifluoromethyl hydroxyalkylation of unprotected 5-phenylthiophen-2-amine has never been described in the literature. Herein, we report the trifluoromethyl hydroxyalkylation methodology we developed in presence of various α-trifluoromethyl ketones (Figure 1d).

## 2. Results

**Scheme 1:**
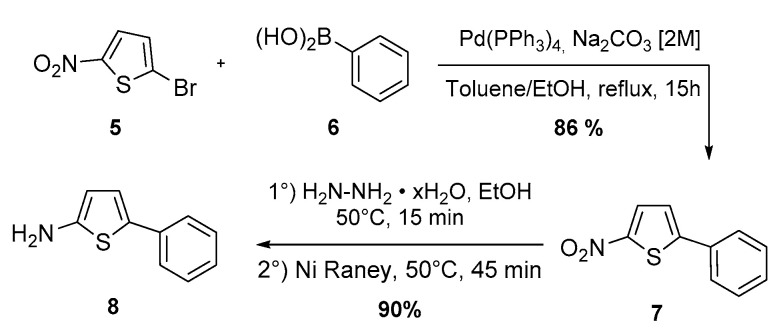
Synthesis of precursor **8**.

To begin, the synthesis of compound 8 has been investigated as described in Scheme 1. 2-bromo-5-nitrothiophene 5 and phenylboronic acid 6, which under Suzuki-Miyaura coupling conditions, developed in our laboratory by Boibessot et al., form intermediate 7 in 86% yield after purification [32]. Then, we realized the reduction in the nitro function, following Zhang and co-workers methodology, in the presence of hydrazine hydrate in absolute ethanol at 50 °C for 15 min, followed by the careful addition of Raney nickel to smoothly yield to 8 [33]. (90% yield after purification).

**Scheme 2:**
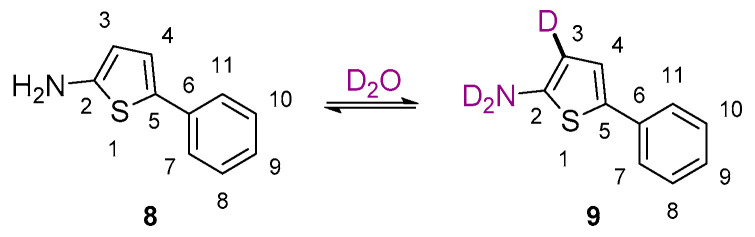
Deuteriation of **8** on C_3_ atom in presence of deuterium oxide.

On running ^1^H NMR analysis of compound **8** in D_2_O deuteriation on C_3_ atom has been observed (Scheme 2). On the ^1^H spectra, H_3_ signal disappears and H_4_ signal appears as a singlet at 7.28 ppm. On ^13^C spectra, C_3_ atom couples with the deuterium it carries to give a triplet at 123.40 ppm (See Supplementary Materials for More Details, Figures S1 and S2).

**Scheme 3:**
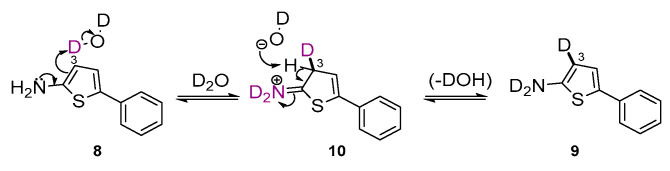
Proposed mechanism for the deuteriation of **9**.

The deuteriation on C_3_ atom seems to be due to the positive mesomeric (+**M**) effect of the amino group responsible for the reactivity shown thereafter.^17^ A plausible mechanism of this deuteriation is proposed in Scheme 3, such as suggested by Garnett and his team [34]. A delocalization of the lone pair of nitrogen atoms would result in a deuteriation on C_3_ atom to generate the intermediate **10** before the rearomatization of the structure to afford **9**.

The reactivity of **8** was then studied in the presence of other electrophiles. When **8** is reacted with *p*-anisaldehyde or acetophenone in toluene under reflux, no reaction occurs and only starting materials are recovered (See Supplementary Materials, Table S1). Facing this lack of reactivity, we decided to use stronger electrophilic compounds to exploit the natural reactivity of 2-AT. α-trifluorinated ketones have been chosen, as suggested in the literature [35]. In that case, when **8** is in the presence of α-trifluorinated ketone **11e** in toluene under reflux, substitution product **12e** is obtained in good yields. It suggests that α-trifluomethyl ketone are harder electrophiles than methyl ketones, following the hard and soft acids and bases theory [36,37]. Temperature has been investigated. Best yield of 83% has been obtained when temperature was set to 120 °C. At 100 °C, the reaction was incomplete and decomposition products have been observed at 140 °C (Table 1, entries 1–3). When an excess of α-trifluorinated ketone **11e** (1.5 and 2.0 equiv.) was reacted with **8**, yields stayed similar (Table 1, entries 4–5).

The reaction occurs under metal and catalyst free conditions, in toluene under reflux for 2 to 5 h. As predicted, the reaction is site selective. This reactivity may be directed by the +**M** effect of the amino group, which confers an enhanced nucleophilic reactivity of the C_3_ atom and allows reaction with α-trifluoromethyl ketones as electrophiles. When acidic catalytic conditions are used (AlCl_3_ or Sc(OTf)_3_) no product is observed, showing that the donor effect of the amino group is sufficient to observe the formation of the desired derivative (See Supplementary Materialsfor more details, Table S2).

**Scheme 4:**
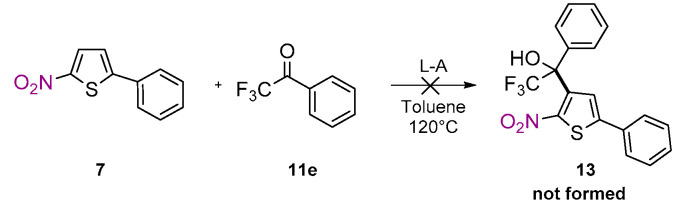
Attempts of hydroxyalkylation of **7**.

Additionally, when the amino group is replaced by the electron withdrawing nitro group on **7**, no substitution product is formed in presence of ketone **11e**. Whether in the presence or absence of acidic catalysis (AlCl_3_ and Sc(OTf)_3_ 10 mol%, see Supplementary Materials for More Details, Table S3), only the starting material has been recovered showing that the amine function is important. The presence of +**M** effect of the amino function is crucial in the reactivity (Scheme 4).

**Scheme 5:**
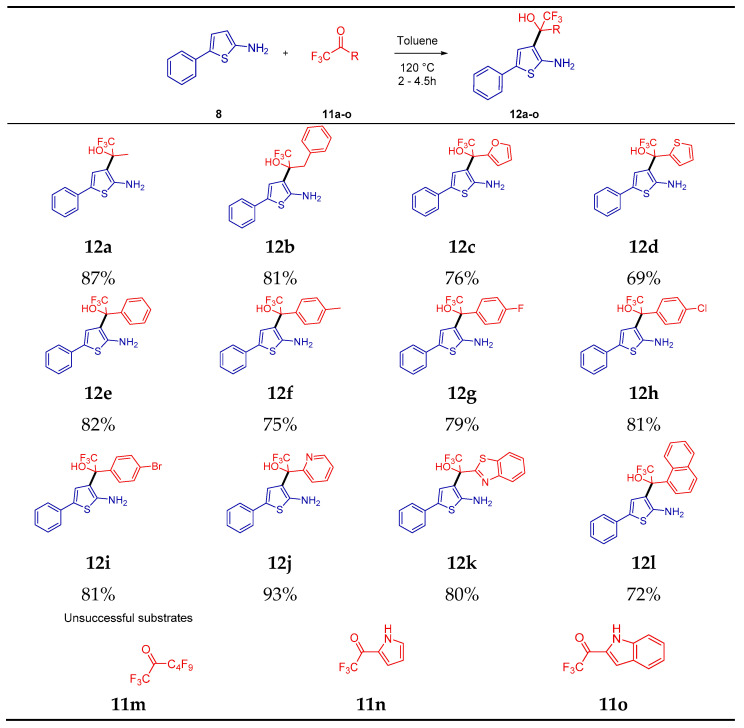
Scope of substituted aminothiophenes. Reactions conditions: 5-phenylthiophen-2-amine **8** (1.0 equiv.), α-trifluoromethyl ketones **11a–o** (1.0 equiv.), toluene (3.5 mL/mmol), under argon for 2–4.5 h, 120 °C. Yields obtained after purification on flash chromatography. Not isolated, only starting materials have been recovered.

To investigate the scope of the proposed methodology, various α-trifluoromethyl ketones **11a–o** were allowed to react with 5-phenylthiophen-2-amine **8** in stoechiometric amounts in toluene under reflux for 2 to 4.5 h (Scheme 5). The aliphatic nature of the group grafted on ketone did not prevent the reactivity and substitution molecules are formed in good yields (**12a**: 87%, **12b**: 81%). Aryls and heteroaryls groups were also investigated and good to excellent yields were obtained with 6-membered rings (**12e–j**: 75–93%), giving slightly better yields than 5-membered rings (**12c**: 76%, **12d**: 69%). Steric hindrance did not seem to be a determining factor because aromatic bicycles reacted smoothly to afford desired compounds in very good yields too (**12k**: 80%, **12l**: 72%). In most cases, the hydroxyalkylation was observed in good to excellent yields after purification with flash chromatography (69–93%). Reaction did not occur in the presence of ketones **11m**, **11n** and **11o**. Anyway, this reaction seems to be substrate-dependent, in light of the absence of reactivity for α-trifluoromethyl ketones **11m–o**. The presence of pyrrole and indole, known to be two rich electron heterocycles, may be responsible for the deactivation of the hard nucleophilic center that is the trifluoromethyl ketones **11n** and **11o** [38]. Considering the perfluoro-2-hexanone **11m**, no example of such reactivity has been reported in the literature for the last 20 years (see Supplementary Materials for More Details, Table S4).

**Scheme 6:**
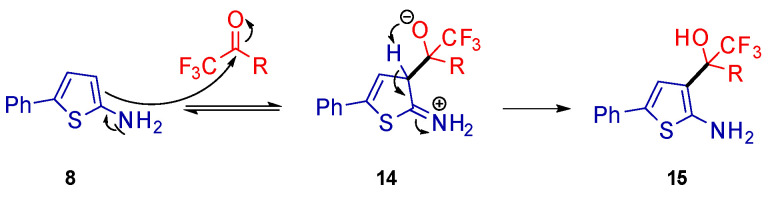
Mechanistic proposal for the coupling reaction with 5-phenyl-2-aminothiophene **8** and α-trifluoromethyl ketone.

A mechanistic proposal is given in Scheme 6 for the conversion of **8** into **15** through a similar mechanism of an electrophilic aromatic substitution [39,40,41]. First step is the attack of C_3_ atom on electrophilic center of trifluoromethyl ketone to afford intermediate **14**. Then, aromatization drives the formation of structure **15**.

**Figure 2:**
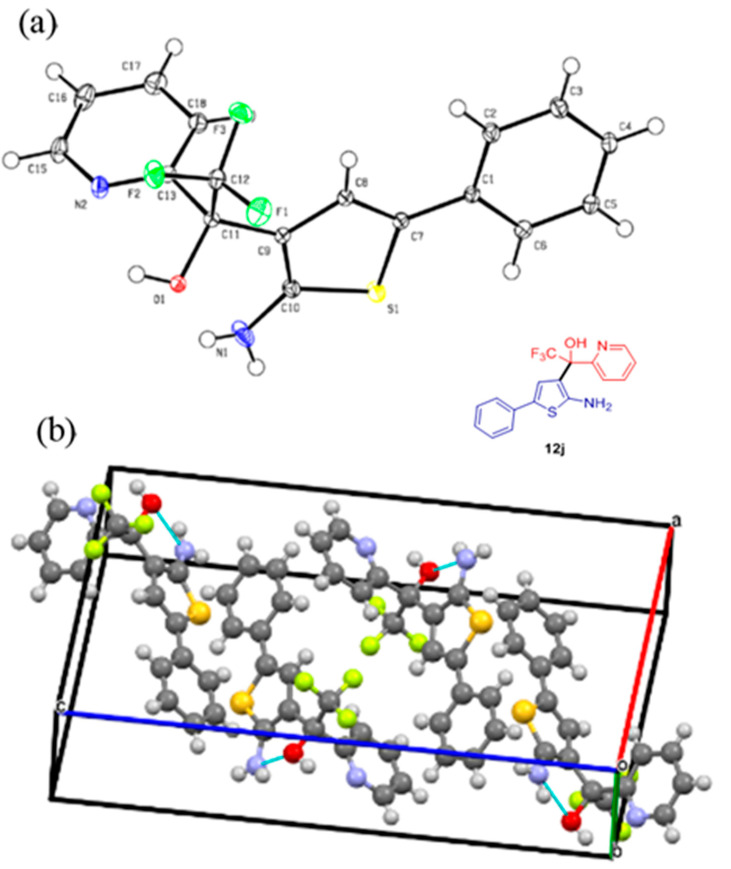
(**a**) XP diagram of compound **12j** with atomic numbering scheme; (**b**) Expansion of the packing diagram of compound **12j** within the crystal mesh trough intra- and intermolecular hydrogen bonds.

An X-ray crystal structure was carried out to establish the authenticity of **12j** structure. We can observe that the structure has a planar part composed of the phenyl and thiophenyl moiety. Then, alkylation observed and assessed on C_2_ atom is composed of a pyridyl group almost perpendicular to both other aromatic cycles. A very strong intramolecular H-bond is observed between the new hydroxyl generated group and the amine group of the thiophenyl moiety [N_1_-H_1c_ ⋯ O_1_ 2.187 Å and 129°] (Figure 2a,b). This bond, stabilizing the whole structure, could drive the reaction. Another one is observed between nitrogen atom of pyridyl group and hydroxyl function [O_1_-H_1c_ ⋯ N_2_ 2.237 Å and 137°]. (See Supplementary Materials, Figures S3 and S4, Tables S5–S9). The expansion of the packing diagram also showed the alternance of R and S enantiomers in the crystal mesh. (Figure 2b). Since no catalysts or chiral auxiliaries were used, we did not expect the reaction to be enantioselective.

## 3. Conclusions

In summary, we have developed an atom-economical approach to synthesize site-specific substituted 5-phenylthiophen-2-amine from simple and commercially available starting materials, namely, α-trifluoromethyl ketones and 5-phenylthiophen-2-amine, by exploiting a trifluoromethyl hydroxyalkylation reaction. The chosen scope shows the variety we can introduce on 5-phenylthiophen-2-amine scaffold. The reaction showed good to excellent yields after purification (69–93%) and with a total chemoselectivity given that only carbon-carbon bond is formed. The site selective introduction of trifluoromethyl hydroxyalkyl groups contrasts with traditionally inserted substituents with -M effect on thiophene scaffold. Moreover, the chemo-and regioselectivity described allows flexibility in substitutions possibilities in drug discovery.

## 4. Materials and Methods

### 4.1. General Experimental Methods

All reagents were purchased from commercial suppliers (Acros Geel—Belgium, Sigma Aldrich L’lsle-d’Abeau Chesnes—France, Alfa Aesar Kandel—Germany and TCI Zwijndrecht—Belgium) and were used without further purification. NMR spectra were recorded with a Bruker Avance 300 spectrometer (300 MHz and 75 MHz for ^1^H and ^13^C NMR, respectively) and Bruker Avance 400 spectrometer (376.5 MHz for ^19^F). Chemical shifts (δ) and coupling constants (*J*) are given in ppm and Hz, respectively, using residual solvent signals as reference for the ^1^H and ^13^C. The following abbreviations are used: s = singlet, d = doublet, t = triplet, q = quartet, br s = broad signal, dd = doublet of doublets, dt = double of triplets, m = multiplet. High-resolution mass spectra (HRMS) were obtained by electrospray using a TOF analyzer Platform. IR spectra were obtained using a Jasco FT-IR 410 instrument as a thin film on NaCl disc as stated; only structurally important peaks (*ῡ*) are presented in cm^−1^. Reactions were monitored with Merck Kieselgel 60F_254_ precoated aluminum silica gel plates (0.25 mm thickness). Melting points were determined on a Stuart scientific SMP10 apparatus and are uncorrected. Flash chromatography was performed on a Grace Reveleris X2 using a 40 µm packed silica cartridge. HPLC analyses were obtained on the Waters Alliance 2795 using the following conditions: Thermo Hypersil C18 column (3 μm, 50 mm × 2.1 mm), 20 °C column temperature, 0.2 mL/min flow rate, photodiodearray detection (210−400 nm), mobile phase consistent of a gradient of water and acetonitrile (each containing 0.1% trifluoroacetic acid). UPLC analyses were obtained on the Waters Acquity H-Class using the following conditions: Waters Acquity BEH C18 column (1.7 μm, 50 × 2.1 mm), 25 °C column temperature, 0.5 mL/min flow rate, photodiodearray detection (TUV−214 nm), mobile phase consistent of a gradient of water and acetonitrile (each containing 0.1% of formic acid).

### 4.2. Preparation of 5-Phenylthiophen-2-amine (***8***)

Starting from 2-bromo-5-nitrothiophene **5** (2.4 mmol; 500 mg), phenylboronic acid **6** (3.6 mmol; 440 mg) and Pd(PPh_3_)_4_ (0.12 mmol; 140 mg) in a mixture of toluene/ethanol (16 mL, 2.3:1, *v/v*) was added a [2 M] of aqueous solution of Na_2_CO_3_ (4.8 mmol; 4.5 mL). The reaction mixture was refluxed over 15 h. The cold solution was diluted with ethyl acetate (50 mL) and filtered through a Celite pad, and the filtrate was diluted with water (60 mL). The aqueous solution was extracted with ethyl acetate (3 × 50 mL). The organic phases were combined, dried over MgSO4, filtered, and concentrated under reduced pressure to give the crude compound. The residue was purified by flash column chromatography (silica gel, AcOEt/Petroleum ether (PE), 0/100 ramping to 100/0, *v/v*) to give the desired compound. 2-nitro-5-phenylthiophene (**7**): Yield: 86% (756 mg); yellow powder: mp 124–126 °C (lit. [32] 123–124 °C); R*_f_*: 0.51 (PE/AcOEt: 8/2). ν_max_/cm^−1^ 1512 (N-O). ^1^H NMR (300 MHz, DMSO-d6) δ 7.47–7.55 (m, 3H, 3H_Ar_), 7.66–7.55 (m, 1H, H_Ar_), 7.80–7.87 (m, 2H, 2H_Ar_), 8.17 (d, 1H, *J* = 4.5 Hz, H_Ar_). ^13^C NMR (DMSO-d_6_, 75 MHz) δ 124.07 (CH_Ar_), 126.29 (2CH_Ar_), 129.54 (2CH_Ar_), 130.35 (CH_Ar_), 131.37 (CH_Ar_), 131.48 (C_Ar_), 149.32 (C_Ar_), 151.52 (C-NO_2_). UPLC: t_R_: 3.15 min; purity: 97%; HRMS: [M + H]^+^ calculated for C_10_H_8_NO_2_S: 206.0276; found: 206.0276.

The synthesis of 5-phenylthiophen-2-amine (**8**) was prepared according to a procedure described by Zhang et al. or with minor modifications thereof [33]. To a solution of 2-nitro-5-phenylthiophene (**7**) (1.71 mmol; 300 mg) and Pd/C (0.28 mmol; 30 mg) in absolute ethanol (5 mL, C = 0.1 M) was added hydrazine hydrate (15 eq). The reaction was stirred at 50 °C for 20 min and an excess of Raney nickel slurry in water (1.2 eq) was slowly added. The reaction was monitored by TLC (PE/AcOEt 70/30). After 1.5 h, when the bubbling ceased, the mixture was cooled to room temperature and filtered through celite. The filtrate was condensed under reduced pressured and the crude was purified on flash silica gel chromatography (PE/AcOEt 100/0 ramping 0/100 *v/v*) to afford the desired product. 5-phenylthiophen-2-amine (**8**): Yield: 90% (232 mg); white powder: mp 127–129 °C. R_f_: 0.64 (PE/EtOAc: 70/30). ν_max_/cm^−1^ 3110 (NH). ^1^H NMR (300 MHz, DMSO-d6) δ 5.77 (br s, 2H, NH_2_), 5.86 (d, *J* = 3.8 Hz, 1H, H_Ar_), 7.01 (d, *J* = 3.8 Hz, 1H, H_Ar_), 7.05–7.12 (m, 1H, H_Ar_), 7.24–7.31 (m, 1H, H_Ar_), 7.35–7.41 (m, 1H, H_Ar_). ^1^H NMR (300 MHz, D_2_O) δ 7.30 (s, 1H, H_Ar_), 7.38–7.50 (m, 3H, 3H_Ar_), 7.64–7.69 (m, 2H, 2H_Ar_). ^13^C NMR (75 MHz, DMSO-d_6_,) δ 104.72 (CH_Ar_), 123.05 (CH_Ar_), 123.39 (2CH_Ar_), 125.01 (C_Ar_), 125.08 (CH_Ar_), 128.80 (2CH_Ar_), 135.04 (C_Ar_), 154.51 (C-NH_2_). ^13^C NMR (75 MHz, D_2_O) δ 122.06 (CH_Ar_), 123.40 (t, *J* = 15.7 Hz, CD_Ar_), 125.53 (2CH_Ar_), 128.48 (CH_Ar_), 129.07 (C_Ar_), 129.22 (2CH_Ar_), 132.67 (CH_Ar_), 142.24 (C-NH_2_). UPLC: t_R_: 3.15 min; purity: 97%; HRMS: [M + H]^+^ calcd for C_10_H_10_NS: 176.0528; found: 176.0527.

### 4.3. Procedure for the Preparation of Trifluorohydroxyalkyl-5-Phenylthiophen-2-amine (***12a–12l***)

In a round bottom flask, 5-phenylthiophen-2-amine (**8**) (1eq, 0.57 mmol; 100 mg), and the corresponding trifluorinated compound (**12a–o**) (1eq, 0.57 mmol) are added in 2mL of dry toluene under reflux and inert atmosphere until complete substrate consumption followed by TLC (PE/AcOEt 7/3) and HPLC (H_2_O/ACN). The cold solution is then diluted with AcOEt (50 mL) and water (50 mL). The aqueous layer is extracted with ethyl acetate (3 × 50 mL) and the combined organic layers are washed with water (50 mL), brine (50 mL), dried over MgSO_4_ and concentrated under reduced pressure to give the crude compound (brown oil). The residue is further purified by flash column chromatography (silica gel, AcOEt/PE, 0/100 ramping to 100/0, *v/v*) to give the desired compound as a brown powder or brown crystals.

2-(2-amino-5-phenylthiophen-3-yl)-1,1,1-trifluoropropan-2-ol (**12a**): Yield: 87% (142 mg); brown powder: mp 135–137 °C. R*_f_*: 0.44 (PE/AcOEt: 70/30 *v/v*). ν_max_/cm^−1^ 3615 (NH), 3362 (OH), 1609 (N-H) and 1144 (C-OH). ^1^H NMR (300 MHz, DMSO-d6) δ 1.67 (s, 3H, CH_3_), 5.87 (br s, 2H, NH_2_), 6.72 (s, 1H, H_Ar_), 7.03 (s, 1H, OH), 7.12 (t, *J* = 7.3 Hz, 1H, H_Ar_), 7.29 (t, *J* = 7.5 Hz, 2H, 2H_Ar_), 7.40 (d, *J* = 8.1 Hz, 2H, 2H_Ar_). ^13^C NMR (75 MHz, DMSO-d6) δ 23.11 (CH_3_), 73.87 (q, ^2^*J*_CF_ = 28.6 Hz, C-CF_3_), 113.42 (C_Ar_), 122.89 (C_Ar_), 123.17 (CH_Ar_), 123.48 (2CH_Ar_), 125.49 (CH_Ar_), 126.60 (q, ^1^*J*_CF_ = 285.8 Hz, CF_3_), 128.84 (2CH_Ar_), 134.43 (C_Ar_), 152.47 (C_Ar_). ^19^F NMR (376 MHz, DMSO-d6) δ −80.50 (CF_3_). HPLC: t_R_: 30.28 min. HRMS: [M + H]^+^ calcd for C_13_H_13_NOSF_3_: 288.0670; found: 288.0685.

2-(2-amino-5-phenylthiophen-3-yl)-1,1,1-trifluoro-3-phenylpropan-2-ol (**12b**): Yield: 81% (168 mg); brown powder: mp 118–119 °C. R*_f_*: 0.37 (PE/AcOEt: 70/30 *v/v*). ν_max_/cm^−1^ 3615 (NH), 3375 (OH), 1596 (N-H) and 1145 (C-OH). ^1^H NMR (300 MHz, DMSO-d6) δ 3.16 (d, *J* = 14.2 Hz, 1H, CH_2a_), 3.51 (d, *J* = 14.2 Hz, 1H, CH_2b_), 5.71 (br s, 2H, NH_2_), 6.92 (s, 1H, H_Ar_), 7.08–7.21 (m, 5H, 5H_Ar_), 7.26–7.35 (m, 4H, 3H_Ar_, OH), 7.37–7.42 (m, 2H, 2H_Ar_).The signal corresponding to CH_2_ appears under the solvent signal on ^13^C spectrum. Yet, the signal appears on DEPT-135 spectrum.^13^C NMR (75 MHz, DMSO-d6) δ 38.82 (CH_2_), 78.04 (q, ^2^*J*_CF_ = 27.2 Hz, C-CF_3_), 110.57 (C_Ar_), 120.78 (C_Ar_), 122.90 (C_Ar,_ CH_Ar_), 123.49 (2CH_Ar_), 125.49 (2CH_Ar_), 126.26 (CH_Ar_), 126.43 (q, ^1^*J*_CF_ = 287.2 Hz, CF_3_), 127.48 (2CH_Ar_), 128.85 (2CH_Ar_), 130.83 (2CH_Ar_), 134.46 (C_Ar_), 135.34 (C_Ar_), 152.85 (C_Ar_). ^19^F NMR (376 MHz, DMSO-d6) δ −76.34 (CF_3_). HPLC: t_R_: 32.13 min. HRMS: [M + H]^+^ calcd for C_19_H_17_F_3_NS: 364.0977; found: 364.0982.

1-(2-amino-5-phenylthiophen-3-yl)-2,2,2-trifluoro-1-(furan-2-yl)ethan-1-ol (**12c**): Yield: 76% (147 mg); brown powder: mp 126–128 °C. R*_f_*: 0.46 (PE/AcOEt: 70/30 *v/v*). ν_max_/cm^−1^ 3615 (NH), 3362 (OH), 1596 (N-H) and 1159 (C-OH). ^1^H NMR (300 MHz, DMSO-d6) δ 5.80 (br s, 2H, NH_2_), 6.57–6.52 (m, 2H, H_Ar_), 6.65 (s, 1H, H_Ar_), 7.18–7.08 (m, 1H, H_Ar_), 7.29 (d, *J* = 1.4 Hz, 2H, H_Ar_), 7.30 (s, 2H, H_Ar_), 7.61 (s, 1H, OH), 7.76 (dd, *J* = 1.7, 0.9, 1H, H_Ar_). ^13^C NMR (75 MHz, DMSO-d6) δ 74.82 (q, ^2^*J*_CF_ = 30.4 Hz, C-CF_3_), 109.21 (CH_Ar_), 110.42 (CH_Ar_), 110.54 (C_Ar_), 122.60 (CH_Ar_), 122.78 (C_Ar_), 123.50 (2CH_Ar_), 125.11 (q, ^1^*J*_CF_ = 286.1 Hz, CF_3_), 125.73 (CH_Ar_), 128.96 (2CH_Ar_), 134.11 (C_Ar_), 143.60 (CH_Ar_), 150.91 (C_Ar_), 153.01 (C_Ar_). ^19^F NMR (376 MHz, DMSO-d6) δ −74.25 (CF_3_). HPLC: t_R_: 30.28 min. HRMS: [M + H]^+^ calcd for C_16_H_12_F_3_NO_2_S: 339.0535; found: 339.0532.

1-(2-amino-5-phenylthiophen-3-yl)-2,2,2-trifluoro-1-(thiophen-2-yl)ethan-1-ol (**12d**): Yield: 69% (140 mg); brown powder: mp 120–121 °C. R*_f_*: 0.46 (PE/AcOEt: 70/30 *v/v*). ν_max_/cm^−1^ 3619 (NH), 3244 (OH), 1606 (N-H) and 1157 (C-OH). ^1^H NMR (300 MHz, DMSO-d6) δ 5.52 (br s, 2H, NH_2_), 6.83 (s, 1H, H_Ar_), 7.04 (t, 1H, *J* = 4.2 Hz, H_Ar_), 7.10–7.16 (m, 2H, H_Ar_), 7.26–7.37 (m, 5H, 4H_Ar_, OH), 7.58 (d, *J* = 4.9 Hz, 1H, H_Ar_). ^13^C NMR (75 MHz, DMSO-d6) δ 76.19 (q, ^2^*J*_CF_ = 27.7 Hz, C-CF3), 113.09 (CH_Ar_), 122.08 (CH_Ar_), 122.89 (CH_Ar_), 123.57 (2CH_Ar_), 125.79 (CH_Ar_), 126.05 (q, ^1^*J*_CF_ = 280.9 Hz, CF_3_), 126.41 (CH_Ar_), 126.75 (CH_Ar_), 126.99 (CH_Ar_), 128.99 (2CH_Ar_), 134.13 (C_Ar_), 143.25 (C_Ar_), 152.88 (C_Ar_). ^19^F NMR (376 MHz, DMSO-d6) δ −76.06 (CF_3_). HPLC: t_R_: 32.27 min. HRMS: [M + H]^+^ calcd for C_16_H_13_F_3_NOS_2_: 356.0391_;_ found: 356.0397.

1-(2-amino-5-phenylthiophen-3-yl)-2,2,2-trifluoro-1-phenylethan-1-ol (**12e**): Yield: 82% (163 mg); brown powder: mp 116–118 °C. R*_f_*: 0.60 (PE/AcOEt: 70/30 *v/v*). ν_max_/cm^−1^ 3376 (NH, OH), 1608 (N-H) and 1147 (C-OH). ^1^H NMR (300 MHz, DMSO-d6) δ 5.50 (br s, 2H, NH_2_), 6.95 (s, 1H, H_Ar_), 7.15 (t, *J* = 7.1 Hz, 1H, H_Ar_), 7.28–7.44 (m, 8H, 7H_Ar_, OH), 7.49 (d, *J* = 7.2 Hz, 2H, 2H_Ar_). ^13^C NMR (75 MHz, DMSO-d6) δ 76.90 (q, ^2^*J*_CF_ = 27.9 Hz, C-CF_3_), 114.35 (C_Ar_), 122.00 (CH_Ar_), 123.00 (C_Ar_), 123.63 (2CH_Ar_), 125.67 (q, ^1^*J*_CF_ = 281.2 Hz, CF_3_), 125.79 (CH_Ar_), 127.16 (2CH_Ar_), 128.02 (2CH_Ar_), 128.35 (CH_Ar_), 129.06 (2CH_Ar_), 134.30 (C_Ar_), 138.80 (C_Ar_), 152.53 (C_Ar_). ^19^F NMR (376 MHz, DMSO-d6) δ −75.54 (CF_3_). HPLC: t_R_: 32.93 min. HRMS: [M + H]^+^ calcd for C_18_H_15_NOSF_3_, 350.0826; found 350.0829.

1-(2-amino-5-phenylthiophen-3-yl)-2,2,2-trifluoro-1-(*p*-tolyl)ethan-1-ol (**12f**): Yield: 75% (155 mg); brown powder: mp 113–115 °C. R*_f_*: 0.56 (PE/AcOEt: 70/30 *v/v*). ν_max_/cm^−1^ 3628 (NH), 3336 (OH), 1610 (N-H) and 1157 (C-OH). ^1^H NMR (300 MHz, DMSO-d6) δ 2.29 (s, 3H, CH_3_), 5.48 (br s, 2H, NH_2_), 6.95 (s, H, H_Ar_), 7.10–7.21 (m, 3H, 3H_Ar_), 7.29–7.42 (m, 7H, 6H_Ar_, OH). ^13^C NMR (75 MHz, DMSO-d6) δ 20.65 (CH_3_), 76.78 (q, ^2^*J*_CF_ = 28.3 Hz, C-CF_3_), 114.47 (C_Ar_), 122.00 (C_Ar_), 122.87 (C_Ar_), 123.57 (2CH_Ar_), 125.70 (CH_Ar_), 126.96 (q, ^1^*J*_CF_ = 279.0 Hz, CF_3_), 127.07 (2CH_Ar_), 128.54 (2CH_Ar_), 129.00 (2CH_Ar_), 134.30 (C_Ar_), 135.83 (C_Ar_), 137.57 (C_Ar_), 152.46 (C_Ar_). ^19^F NMR (376 MHz, DMSO-d6) δ −76.22 (CF_3_). HPLC: t_R_: 34.05 min. HRMS: [M + H]^+^ calcd for C_19_H_17_F_3_NOS: 346.0986; found: 364.0977.

1-(2-amino-5-phenylthiophen-3-yl)-2,2,2-trifluoro-1-(4-fluorophenyl)ethan-1-ol (**12g**): Yield: 79% (165 mg); brown powder: mp 115–116 °C. R*_f_*: 0.46 (PE/AcOEt: 70/30 *v/v*). ν_max_/cm^−1^ 3612 (NH), 3376 (OH), 1609 (N-H) and 1144 (C-OH). ^1^H NMR (300 MHz, DMSO-d6) δ 5.52 (br s, 2H, NH_2_), 6.95–7.00 (m, 1H, H_Ar_), 7.11–7.18 (m, 1H, H_Ar_), 7.21 (t, *J* = 8.9 Hz, 2H, 2H_Ar_), 7.32 (t, *J* = 7.7 Hz, 2H, 2H_Ar_), 7.37–7.42 (m, 2H, 2H_Ar_), 7.44 (s, 1H, OH), 7.48–7.54 (m, 2, 2H_Ar_). ^13^C NMR (75 MHz, DMSO-d6) δ 76.51 (q, ^2^*J*_CF_ = 28.7 Hz, C-CF_3_), 114.05 (CH_Ar_), 114.78 (d, ^2^*J*_CF_ = 22.5 Hz, 2CH_Ar_), 121.75 (CH_Ar_), 123.07 (C_Ar_), 123.63 (2CH_Ar_), 125.77 (CH_Ar_), 126.22 (q, ^1^*J*_CF_ = 286.5 Hz, CF_3_), 129.00 (2CH_Ar_), 129.45 (d, ^3^*J*_CF_ = 8.3 Hz, 2CH_Ar_), 134.25 (C_Ar_), 134.88 (C_Ar_), 152.63 (C_Ar_), 161.96 (d, ^1^*J*_CF_ = 243.0 Hz, C_Ar_-F). ^19^F NMR (376 MHz, DMSO-d6) δ −75.15 (CF_3_), −114.21 (C_Ar_-F). HPLC: t_R_: 30.28min. HRMS: [M + H]^+^ calcd for C_18_H_14_F_4_NOS: 368.0727; found: 368.0725.

1-(2-amino-5-phenylthiophen-3-yl)-1-(4-chlorophenyl)-2,2,2-trifluoroethan-1-ol (**12h**): Yield: 81% (177 mg); brown powder: mp 120–122 °C. R*_f_*: 0.46 (PE/AcOEt: 70/30 *v/v*). ν_max_/cm^−1^ 3601 (NH), 3349 (OH), 1596 (N-H) and 1136 (C-OH). ^1^H NMR (300 MHz, DMSO-d6) δ 5.53 (br s, 2H, NH_2_), 6.98 (s, 1H, H_Ar_), 7.15 (t, *J* = 7.2 Hz, 1H, H_Ar_), 7.32 (t, *J* = 7.7 Hz, 2H, 2H_Ar_), 7.38–7.42 (m, 2H, 2H_Ar_), 7.45–7.51 (m, 5H, 4H_Ar_, OH). ^13^C NMR (75 MHz, DMSO-d6) δ 76.44 (d, ^2^*J*_CF_ = 28.8 Hz, C-CF_3_), 113.80 (C_Ar_), 121.67 (CH_Ar_), 123.11 (C_Ar_), 123.63 (2CH_Ar_), 123.92 (q, ^1^*J*_CF_ = 272.3 Hz, CF_3_), 125.79 (CH_Ar_), 127.99 (2CH_Ar_), 129.00 (2CH_Ar_), 129.17 (2CH_Ar_), 133.12 (C_Ar_), 134.21 (C_Ar_), 137.70 (C_Ar_), 152.68 (C_Ar_). ^19^F NMR (376 MHz, DMSO-d6) δ −75.10 (CF_3_). HPLC: t_R_: 30.28 min. HRMS: [M + H]^+^ calcd for C_18_H_14_ClF_3_NOS: 384.0431; found: 384.0428

1-(2-amino-5-phenylthiophen-3-yl)-1-(4-bromophenyl)-2,2,2-trifluoroethan-1-ol (**12i**): Yield: 81% (198 mg); brown powder: mp 110–112 °C. R*_f_*: 0.46 (PE/AcOEt: 70/30 *v/v*). ν_max_/cm^−1^ 3601 (NH), 3348 (OH), 1596 (N-H) and 1135 (C-OH). ^1^H NMR (300 MHz, DMSO-d6) δ 5.53 (br s, 2H, NH_2_), 6.98 (s, 1H, H_Ar_), 7.15 (t, *J* = 7.2 Hz, 1H, H_Ar_), 7.32 (t, *J* = 7.7 Hz, 2H, 2H_Ar_), 7.37–7.46 (m, 4H, 4H_Ar_), 7.49 (s, 1H, OH), 7.55–7.63 (m, 2H, 2H_Ar_). ^13^C NMR (75 MHz, DMSO-d6) δ 76.55 (q, ^2^*J*_CF_ = 28.52 Hz, C-CF_3_), 113.78 (C_Ar_), 121.65 (CH_Ar_), 121.83 (C_Ar_), 123.14 (C_Ar_), 123.63 (2CH_Ar_), 125.29 (q, ^1^*J*_CF_ = 285.6 Hz, CF_3_) 125.77 (CH_Ar_), 128.98 (2CH_Ar_), 129.48 (2CH_Ar_), 130.91 (2CH_Ar_), 134.21 (C_Ar_), 138.14 (C_Ar_), 152.69 (C_Ar_). ^19^F NMR (376 MHz, DMSO-d6) δ −75.10 (CF_3_). HPLC: t_R_: 30.28 min. HRMS: [M + H]^+^ calcd for C_18_H_14_BrF_3_NOS: 427.9926; found: 427.9917.

1-(2-amino-5-phenylthiophen-3-yl)-2,2,2-trifluoro-1-(pyridin-3-yl)ethan-1-ol (**12j**): Yield: 93% (185 mg); brown crystals: mp 121–122 °C. R*_f_*: 0.26 (PE/AcOEt: 80/20 *v/v*). ν_max_/cm^−1^ 3599 (NH), 3389 (OH) and 1169 (C-OH). ^1^H NMR (300 MHz, DMSO-d6) δ 5.78 (br s, 2H, NH_2_), 6.95 (s, 1H, H_Ar_), 7.13 (t, *J* = 6.7 Hz, 1H, H_Ar_), 7.24–7.38 (m, 4H, 4H_Ar_), 7.39–7.45 (m, 1H, H_Ar_), 7.47 (s, 1H, OH), 7.69 (d, *J* = 8.1 Hz, 1H, H_Ar_), 7.90 (t, *J* = 7.8 Hz, 1H, H_Ar_), 8.62 (d, *J* = 4.7 Hz, 1H, H_Ar_). ^13^C NMR (75 MHz, DMSO-d6) δ 77.97 (q, ^2^*J*_CF_ = 28.3 Hz, C-CF_3_), 112.69 (C_Ar_), 122.25 (CH_Ar_), 122.59 (CH_Ar_), 122.95 (C_Ar_), 123.52 (2CH_Ar_), 123.68 (CH_Ar_), 125.64 (q, ^1^*J*_CF_ = 276.4 Hz, CF_3_), 125.71 (CH_Ar_), 128.97 (2CH_Ar_), 134.22 (C_Ar_), 137.46 (CH_Ar_), 147.72 (CH_Ar_), 152.53 (C_Ar_), 157.48 (C_Ar_). ^19^F NMR (376 MHz, DMSO-d6) δ −74.25 (CF_3_). HPLC: t_R_: 31.37 min. HRMS: [M + H]^+^ calcd for C_17_H_14_N_2_OF_3_S: 351.0779_;_ found: 351.0793.

1-(2-amino-5-phenylthiophen-3-yl)-1-(benzo[d]thiazol-2-yl)-2,2,2-trifluoroethan-1-ol (**12k**): Yield: 80% (185 mg); brown powder: mp 115–117 °C. R*_f_*: 0.46 (PE/AcOEt: 70/30 *v/v*). ν_max_/cm^−1^ 3606 (NH), 3376 (OH), 1609 (N-H) and 1157 (C-OH). ^1^H NMR (300 MHz, DMSO-d6) δ 6.01 (br s, 2H, NH_2_), 7.08 (s, 1H, H_Ar_), 7.10–7.17 (m, 1H, H_Ar_), 7.26–7.36 (m, 4H, 4H_Ar_), 7.47–7.60 (m, 2H, 2H_Ar_), 8.08–8.18 (m, 2H, 2H_Ar_) 8.64 (s, 1H, OH). ^13^C NMR (75 MHz, DMSO-d6) δ 77.60 (q, ^2^*J*_CF_ = 29.9 Hz, C-CF_3_), 110.61 (C_Ar_), 122.23 (CH_Ar_), 122.35 (CH_Ar_), 123.38 (CH_Ar_), 123.59 (2CH_Ar_), 124.66 (q, ^1^*J*_CF_ = 287.8 Hz, CF_3_), 125.87 (CH_Ar_), 125.90 (CH_Ar_), 126.47 (CH_Ar_), 128.99 (2CH_Ar_), 133.96 (C_Ar_), 134.67 (C_Ar_), 152.48 (C_Ar_), 153.15 (2C_Ar_), 171.32 (C_Ar_). ^19^F NMR (376 MHz, DMSO-d6) δ −75.14 (CF_3_). HPLC: t_R_: 30.28 min. HRMS: [M + H]^+^ calcd for C_19_H_14_F_3_N_2_OS_2_: 407.0494; found: 407.0500.

1-(2-amino-5-phenylthiophen-3-yl)-2,2,2-trifluoro-1-(naphthalen-2-yl)ethan-1-ol (**12l**): Yield: 72% (164 mg); brown powder: mp 127–128 °C. R*_f_*: 0.60 (PE/AcOEt: 70/30 *v/v*). ν_max_/cm^−1^ 3575 (NH), 3336 (OH), 1609 (N-H) and 1134 (C-OH). ^1^H NMR (300 MHz, DMSO-d6) δ 5.84 (br s, 2H, NH_2_), 6.37 (s, 1H, H_Ar_), 6.99–7.34 (m, 5H, 5H_Ar_), 7.35–7.50 (m, 2H, 2H_Ar_), 7.57 (t, *J* = 7.8 Hz, 1H, H_Ar_), 7.74 (s, 1H, H_Ar_), 7.77 (br s, 1H, OH), 7.93 (d, *J* = 7.2 Hz, 1H, H_Ar_), 7.99 (d, *J* = 8.4 Hz, 1H, H_Ar_), 8.43 (d, *J* = 8.6 Hz, 1H, H_Ar_). ^13^C NMR (75 MHz, DMSO-d6) δ 79.47 (q, ^2^*J*_CF_ = 26.9 Hz), 113.04 (C_Ar_), 122.88 (CH_Ar_), 122.97 (CH_Ar_), 123.33 (2CH_Ar_), 123.42 (C_Ar_), 124.54 (CH_AR_), 125.61 (3CH_Ar_), 126.21 (q, ^1^*J*_CF_ = 281.2 Hz, CF_3_), 126.86 (CH_Ar_), 128.74 (CH_Ar_), 128.90 (2CH_Ar_), 129.83 (CH_Ar_), 130.90 (C_Ar_), 133.96 (C_Ar_), 134.41 (C_Ar_), 134.52 (C_Ar_), 151.68 (C_Ar_). ^19^F NMR (376 MHz, DMSO-d6) δ −74.25 (CF_3_). HPLC: t_R_: 33.70 min. HRMS: [M + H]^+^ calcd for C_22_H_17_F_3_NOS: 400.0977; found: 400.095.

### 4.4. Crystallographic Data

CCDC 2083160 contains the supplementary crystallographic data for this paper. These data can be obtained free of charge via http://www.ccdc.cam.ac.uk/conts/retrieving.html (or from the CCDC, 12 Union Road, Cambridge CB2 1EZ, UK; Fax: +44 1223 336033; E-mail: deposit@ccdc.cam.ac.uk)

## Figures and Tables

**Table 1:**
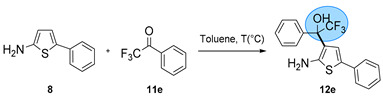
Optimization studies for the synthesis of **12e**.

Entry	11e Equivalent (eq.)	T (°C)	Yield (%) ^b^
1 ^a^	1	100	70%
2 ^a^	1	120	83%
3 ^a^	1	140	47%
4 ^a^	1.5	120	82%
5 ^a^	2	120	81%

^a^ Experiment conducted in toluene. ^b^ Yields obtained after purification on flash chromatography.

## Data Availability

Not applicable.

## Supplementary Materials

The following are available online, Figure S1: ^1^H spectra of **9** in D_2_O (singlet at 7.28 ppm; 300 MHz); Figure S2: ^13^C spectra of **9** in D_2_O (triplet at 123.40 ppm; 75 MHz); Figure S3: Crystal structure of compounds **12j**; Figure S4: (A) XP diagram of compound **12j** with atomic numbering scheme; (B) Expandation of the packing diagram of compound **12j** within the crystal mesh trough intra and intermolecular hydrogen bonds; Table S1: Optimization attempts for the synthesis of **16**–**17**; Table S2: Optimization studies for the synthesis of **12e**; Table S3: Optimization attempts for the synthesis of **13**; Table S4: Kinetics considerations following HPLC spectra; Table S5: Crystal data and structure refinement details for **12j**; Table S6: Bond lengths for **12j** (Å); Table S7: Bond angles for **12j** (°); Table S8: Torsion angles for **12j** (°); Table S9: Hydrogen bond distances (Å) and angles for **12j** (°). Characterization of compounds (^1^H 300MHz, ^13^C 75MHz, ^19^F NMR 376 MHz, DEPT-135 in DMSO-d_6_; HRMS, HPLC).

## Sample Availability

Samples of the compounds are available from the authors.
