# Supplementary material for: Catalyst-Free Site Selective Hydroxyalkylation of 5-Phenylthiophen-2-amine with α-Trifluoromethyl Ketones through Electrophilic Aromatic Substitution
Source: Molecules. 2022 Jan 29;27(3):925. doi: 10.3390/molecules27030925 (PMC8839828; doi:10.3390/molecules27030925)
Supplement: Supplementary file 1 [file molecules-27-00925-s001.zip › molecules-1581986-supplementary.pdf]

- Supporting Information –

Catalyst-Free Site Selective Hydroxyalkylation of 5-phenylthiophen-2-amine with α-trifluoromethyl Ketones through Electrophilic Aromatic Substitution

Valentin Duvauchelle, David Bénimélis, Patrick Meffre and Zohra Benfodda*

UPR CHROME, Université de Nîmes, CEDEX 1, F-30021 Nîmes, France

*E-mail: zohra.benfodda@unimes.fr

Table of Contents

		Page
1.	**General Information**	S2
2.	**Spectra of 9 in D_2O**	S3
3.	**Condition optimization on the synthesis of trifluorohydroxyalkyl-5-phenylthiophen-2-amine**	S4
4.	**Kinetics studies**	S5
5.	**X-ray crystallographic supporting data**	S8
6.	**References**	S15
7.	**Characterization of compounds (^1H, ^{13}C, DEPT-135, HRMS, HPLC)**	S16

General Information

All reagents were purchased from commercial suppliers (Acros, Sigma Aldrich, Alfa Aeser and TCI) and were used without further purification. NMR spectra were recorded with a Bruker Avance 300 spectrometer (300 MHz and 75 MHz for ^{1}H and ^{13}C NMR, respectively) and Bruker Avance 400 spectrometer (376.5 MHz for ^{19}F). Chemical shifts (δ) and coupling constants (*J*) are given in ppm and Hz, respectively, using residual solvent signals as reference for the ^{1}H and ^{13}C. The following abbreviations are used: s = singlet, d = doublet, t = triplet, q = quartet, br s = broad signal, dd = doublet of doublets, dt = double of triplets, m = multiplet. High-resolution mass spectra (HRMS) were obtained by electrospray using a TOF analyzer Platform. IR spectra were obtained using a Jasco FT-IR 410 instrument as a thin film on NaCl disc as stated; only structurally important peaks ($\bar{v}$) are presented in cm^{-1}. Reactions were monitored with Merck Kieselgel $60F_{254}$ precoated aluminum silica gel plates (0.25 mm thickness). Melting points were determined on a Stuart scientific SMP10 apparatus and are uncorrected. Flash chromatography was performed on a Grace Reveleris X2 using a 40µm packed silica cartridge. HPLC analyses were obtained on the Waters Alliance 2795 using the following conditions: Thermo Hypersil C18 column (3 µm, 50 mm × 2.1 mm), 20 °C column temperature, 0.2 mL/min flow rate, photodiodearray detection (210− 400 nm), mobile phase consistent of a gradient of water and acetonitrile (each containing 0.1% trifluoroacetic acid). UPLC analyses were obtained on the Waters Acquity H-Class using the following conditions: Waters Acquity BEH C18 column (1.7 µm, 50 × 2.1 mm), 25 °C column temperature, 0.5 mL/min flow rate, photodiodearray detection (TUV − 214 nm), mobile phase consistent of a gradient of water and acetonitrile (each containing 0.1% of formic acid).

Spectra of compound 9 (^{1}H 300MHz, ^{13}C 75MHz, D_2O)

Figure S1 ^{1}H spectra of **9** in D_2O (singlet at 7.28 ppm; 300 MHz).

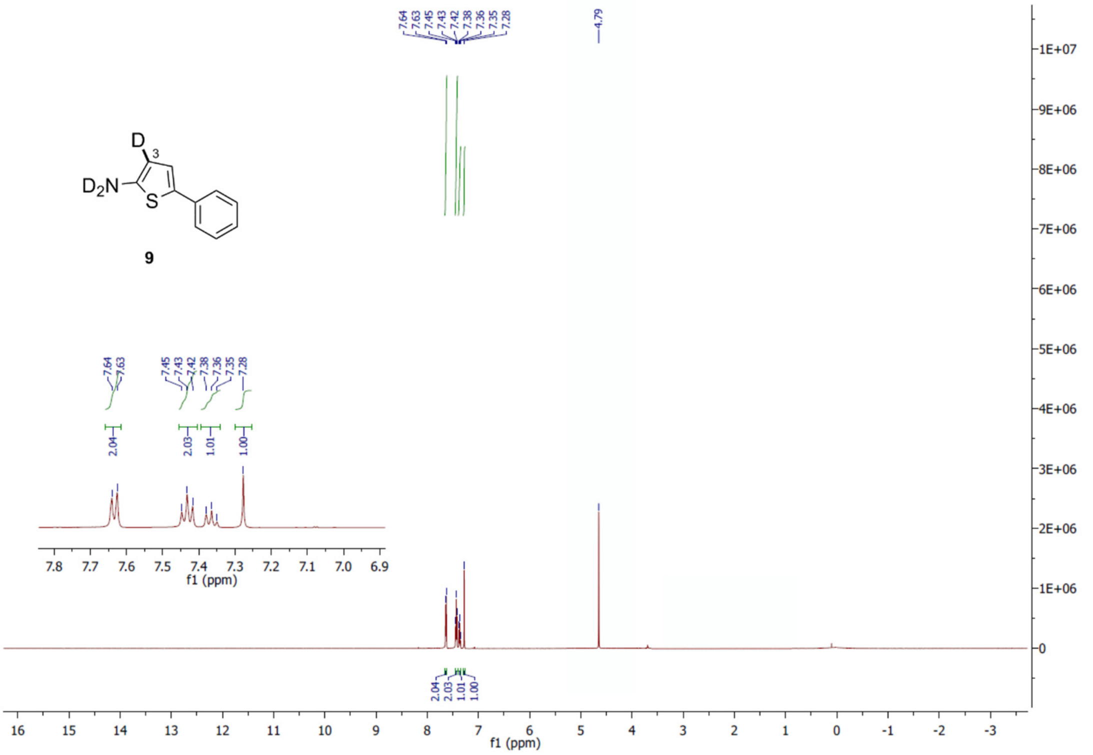

Figure. S2 ^{13}C spectra of **9** in D_2O (triplet at 123.40 ppm; 75 MHz).

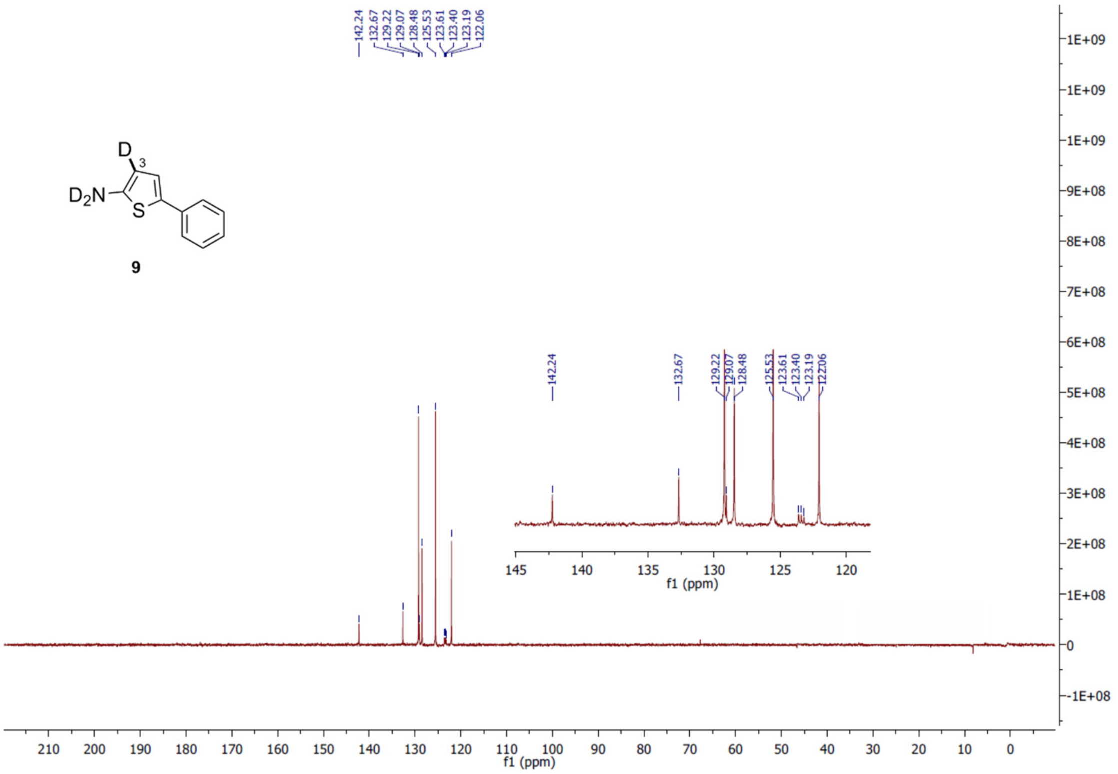

Condition optimization on the synthesis of trifluorohydroxyalkyl-5-phenylthiophen-2-amine

Table S1 Optimization attempts for the synthesis of 16-17

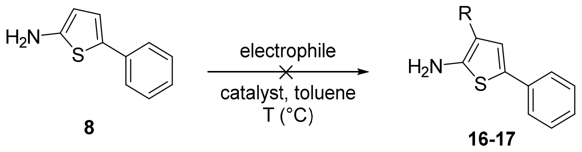

entry	Electrophile	catalyst	T(°C)	Yield[b] (%)
1	Acetophenone	None	120	/
2	Acetophenone	$AlCl_3$ (0.1 eq)	120	/
3	Acetophenone	$Sc(OTF)_3$ (0. 1eq)	120	/
4	*p*-anisaldehyde	None	120	/
5	*p*-anisaldehyde	$AlCl_3$ (0.1 eq)	120	/
6	*p*-anisaldehyde	$Sc(OTf_3)$ (0.1 eq)	120	/

[a]General conditions: **8** (0.57 mmol), electrophile (0.57 mmol), solvent (2mL), stirred at 120°C for 24 hours under argon atmosphere in round bottom flask.[b]Only starting materials are recovered.

Table S2 Optimization studies for the synthesis of 12e

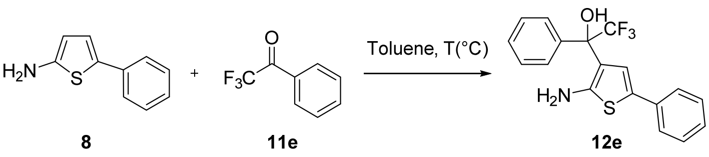

Entry	**11e** quantity	T(°C)	Yield (%)[b]
1[a]	1 eq.	100	70%
2[a]	1 eq.	120	83%
3[a]	1eq.	140	47%
4[a]	1.5 eq.	120	82%
5[a]	2 eq.	120	81%

[a]General conditions: **8** (0.57 mmol), electrophile **11e** (0.57 mmol), Toluene (2mL), stirred at 120°C for 4 hours under argon atmosphere in round bottom flask. [b]Yields obtained after purification on flash chromatography.

Table S3 Optimization attempts for the synthesis of 13

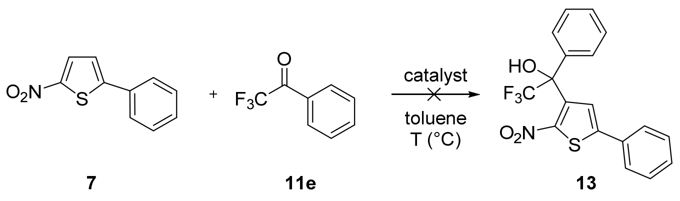

entry	catalyst	T(°C)	Yield (%)
1	None	120	/
2	$AlCl_3$ (0.1 eq)	120	/
3	$Sc(OTF)_3$ (0. 1eq)	120	/

[a]General conditions: **7** (0.57 mmol), electrophile **11e** (0.57 mmol), toluene (2mL), stirred at 120°C for 24 hours under argon atmosphere in round bottom flask.

Kinetics studies

Table S4 Kinetics considerations following HPLC spectra

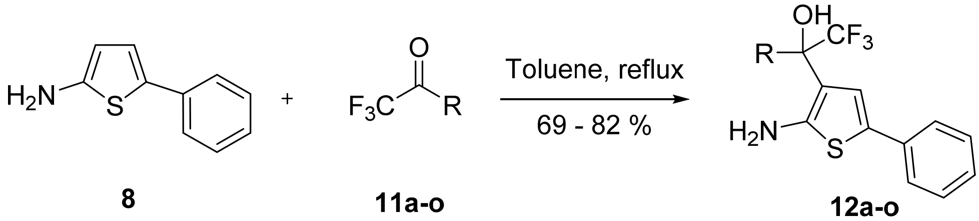

8 11a-o 12a-o

#	Structure	HPLC Yield[a] (%)	Starting material **8** remaining[a] (%)	Byproducts[a] (%)	Reactional time (h)
**12a**	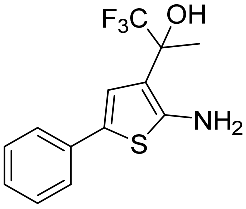	92	0	8	3.5
**12b**	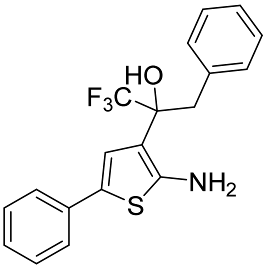	84	0	16	3
**12c**	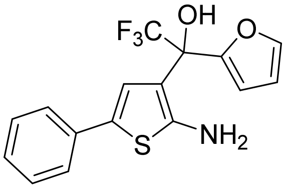	80	7	13	3.5
**12d**	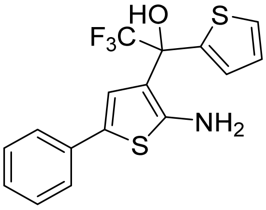	79	5	16	4.5

**12e**	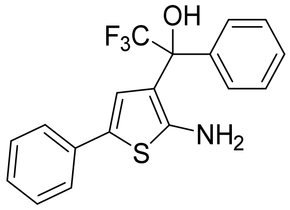	93	0	7	3
**12f**	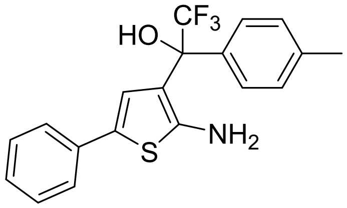	80	1	19	4
**12g**	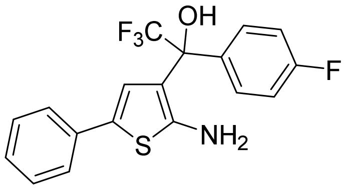	85	1	14	2.5
**12h**	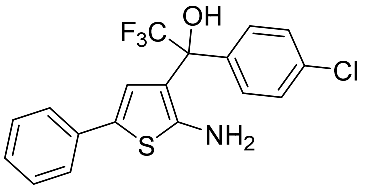	85	0	15	3
**12i**	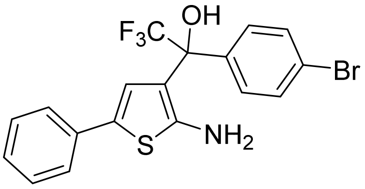	86	0	14	3.5
**12j**	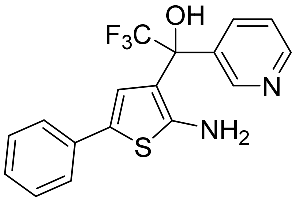	97	0	3	2
**12k**	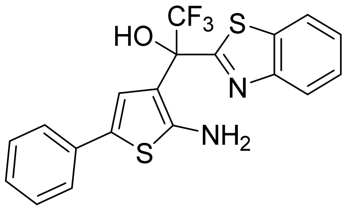	84	0	16	3

**12l**	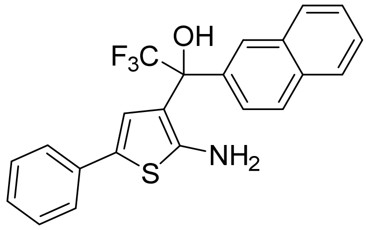	78	0	22	5
**12m**	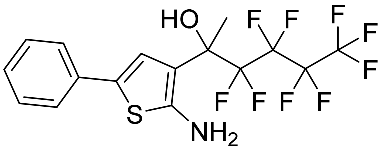	0	/	/	/
**12n**	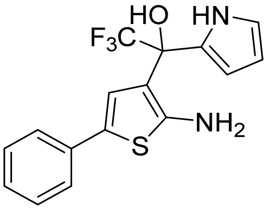	0	/	/	/
**12o**	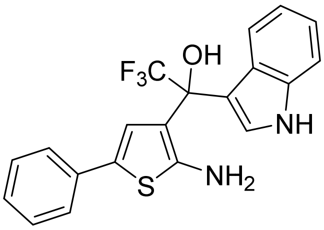	0	/	/	/

[a]Determination realized *via* integration of peaks with DAD HPLC results.

X-ray crystallographic supporting data

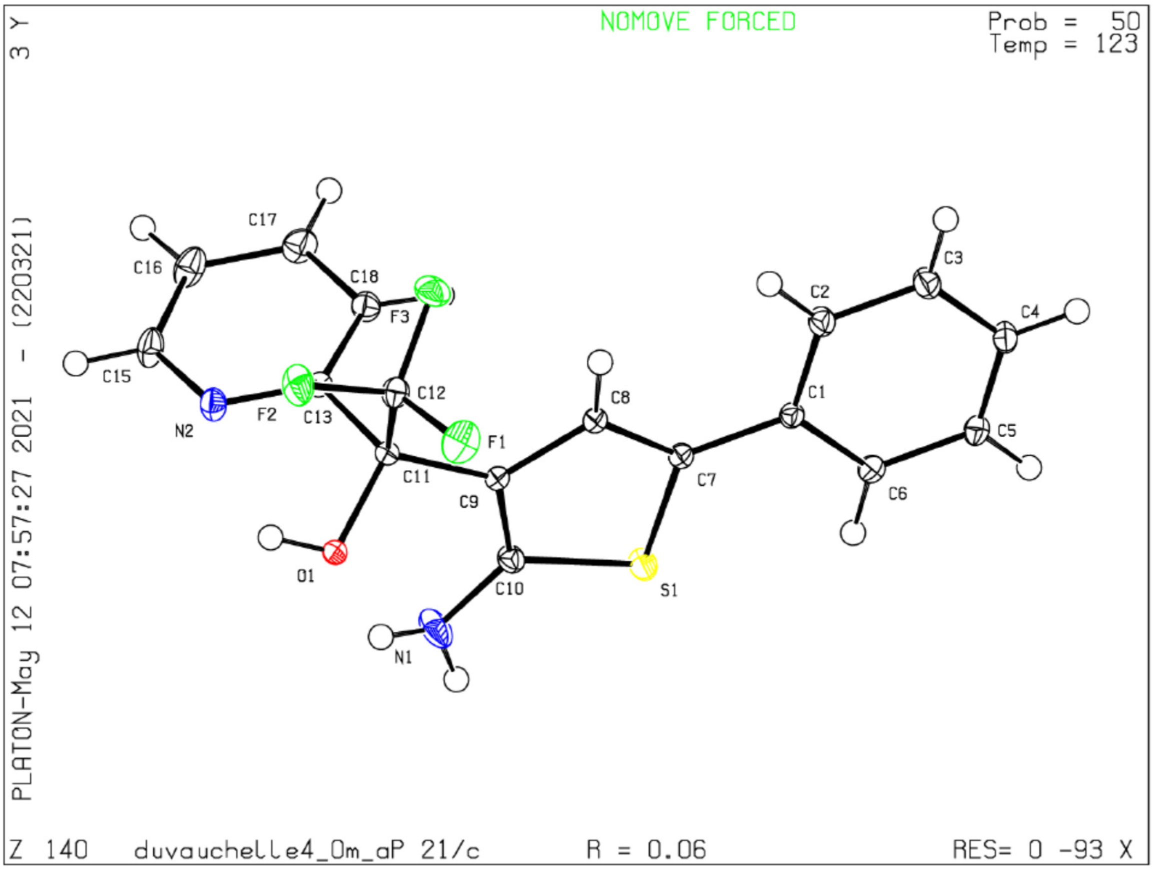

Figure S3 Crystal structure of compounds **12j**

The X-ray single crystal data of **12j**, were collected with monochromated Mo-Kαradiation (0.71073 Å) on a Bruker Apex3 CCD Bruker diffractometer. Intensity data were collected at 123 K using θ-scan method. Data reduction of each compound was carried out using the Bruker SAINT software. Multi-scan absorption correction was applied to all intensity data using the SADABS 2016/2 program.[2] The structures were solved by a combination of direct methods with SHELXT-2014/5 and refined with full-matrix least-squares based on F^2 using SHELXL2018/3.[3] The hydrogen atoms were treated by a mixture of independent and constrained refinement. Molecular and the crystal packing diagrams were drawn with Mercury software. Bruker SHELXTL has been used to prepare material for publication and realize molecular graphics. All of the crystal data are described in supporting information. The crystal structures were deposited with the Cambridge Crystallographic Database Centre (CCDC) and given the number CCDC 2083160.

A colorless plate-like specimen of compound **12j** ($C_{17}H_{13}F_3N_2OS$) with approximate dimensions 0.17 x 0.12 x 0.03 mm has been used. **12j** crystallized in the monoclinic crystal system using the space group P $2_{1/c}$. Short contacts are apparent between two molecules in the solid state between sulfur atom of the thiophenyl and C_1 [C_1 ••• S_1 3.387 Å], F_3 atom of the trifluoride group and H_{18} [C_{12}-F_3 ••• H_{18} 2.551 Å 143°] and between oxygen from the hydroxyl group and aromatics hydrogens, carbon and nitrogen [C_5-H_5 ••• O_1 2.495 Å 160°; C_{13}- H_{13} ••• O_1 2.715 Å 146°; C_{15}-H_{15} ••• O_1 2.691 Å 108°; O_1 ••• N_2 2.237 Å] (Fig. S4AB).

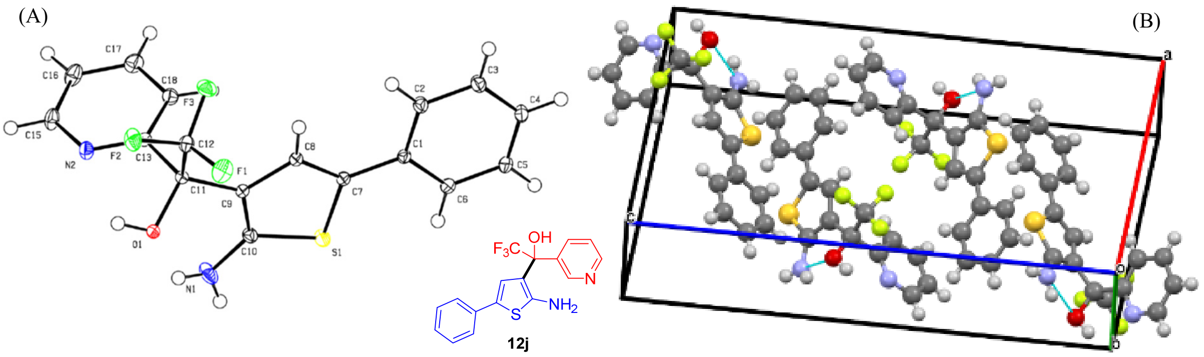

Figure S4 (A) XP diagram of compound **12j** with atomic numbering scheme; (B) Expandation of the packing diagram of compound **12j** within the crystal mesh trough intra and intermolecular hydrogen bonds

Table S5 Crystal data and structure refinement details for **12j**

Identification code	**12j**
Formula	$C_{17}H_{13}F_3N_2OS$
M.W. (g.mol^{-1})	350.3592
Crystal system, space group	Monoclinic, P21/*c*
d_{calcd} (g.cm^{-3})	1.525
Temperature (K)	123
a, b, c (Å)	10.5726(6), 6.8807(3), 21.0661(11)
α (°)	90
β (°)	95.232(2)
γ (°)	90
V (Å^3)	1526.11 (14)
Z	4
Radiation type	Mo *Kα*
μ (mm^{-1})	0.25
Crystal size (mm)	0.17 × 0.12 × 0.03
*R*int	0.054
(sin θ/λ)max (Å−1)	0.913
$R[F2 > 2\sigma(F2)]$, $wR(F2)$, S	0.056, 0.125, 1.10
$\Delta\rho_{max}$, $\Delta\rho_{min}$ (e Å^{-3})	0.69, −0.43
Packing coefficient	0.707
F0000	720
CCDC number	2083160

Table S6 Bond lengths for **12j** (Å).

S1-C10	1.461(1)	C7-C1	1.461(1)
S1-C7	1.405(1)	C1-C2	1.405(1)
F3-C12	1.403(1)	C1-C6	1.403(1)
F1-C12	0.95	C5-H5	0.95
F2-C12	1.389(1)	C5-C6	1.389(1)
O1-C11	0.95	C18-H18	0.95
O1-H1C	1.393(1)	C18-C13	1.393(1)
N2-C13	1.387(1)	C18-C17	1.387(1)
N2-C15	1.537(1)	C13-C11	1.537(1)
N1-C10	1.543(1)	C11-C12	1.543(1)
N1-H1A	0.95	C2-H2	0.95
N1-H1B	1.389(1)	C2-C3	1.389(1)
C9-C8	0.95	C3-H3	0.95
C9-C10	0.95	C6-H6	0.95
C9-C11	0.95	C17-H17	0.95
C8-H8	1.387(2)	C17-C16	1.387(2)
C8-C7	0.95	C15-H15	0.95
C4-H4	1.385(2)	C15-C16	1.385(2)
C4-C5	0.95	C16-H16	0.95
C4-C3			

Table S7 Bond angles for **12j** (°).

C10-S1-C7	92.33(5)	C18-C13-C11	121.60(8)
C11-O1-H1C	106(1)	O1-C11-C9	108.21(7)
C13-N2-C15	117.57(9)	O1-C11-C13	110.80(7)
C10-N1-H1A	114(1)	O1-C11-C12	106.00(7)
C10-N1-H1B	119(1)	C9-C11-C13	111.65(7)
H1A-N1-H1B	120(2)	C9-C11-C12	111.83(7)
C8-C9-C10	111.73(8)	C13-C11-C12	108.21(7)
C8-C9-C11	126.86(8)	C1-C2-H2	119.66
C10-C9-C11	121.22(8)	C1-C2-C3	120.68(9)
C9-C8-H8	122.88	H2-C2-C3	119.7
C9-C8-C7	114.24(8)	C4-C3-C2	120.62(9)
H8-C8-C7	122.88	C4-C3-H3	119.7
H4-C4-C5	120.4	C2-C3-H3	119.7
H4-C4-C3	120.4	F3-C12-F1	107.14(8)
C5-C4-C3	119.22(9)	F3-C12-F2	106.78(8)
S1-C10-N1	119.66(8)	F3-C12-C11	112.32(8)
S1-C10-C9	111.51(7)	F1-C12-F2	107.65(8)
N1-C10-C9	128.79(9)	F1-C12-C11	112.38(8)
S1-C7-C8	110.18(6)	F2-C12-C11	110.28(8)
S1-C7-C1	120.12(6)	C1-C6-C5	121.11(9)
C8-C7-C1	129.70(8)	C1-C6-H6	119.4
C7-C1-C2	120.98(8)	C5-C6-H6	119.4
C7-C1-C6	121.05(8)	C18-C17-H17	120.5
C2-C1-C6	117.97(8)	C18-C17-C16	118.9(1)
C4-C5-H5	119.8	H17-C17-C16	120.5
C4-C5-C6	120.39(9)	N2-C15-H15	118.3
H5-C5-C6	119.8	N2-C15-C16	123.3(1)
H18-C18-C13	120.67	H15-C15-C16	118.4
H18-C18-C17	120.7	C17-C16-C15	118.5(1)
C13-C18-C17	118.64(9)	C17-C16-H16	120.8
N2-C13-C18	123.02(8)	C15-C16-H16	120.8
N2-C13-C11	115.37(8)		

Table S8 Torsion angles for **12j** (°).

C7-S1-C10-N1	177.08(9)	C7-C1-C2-C3	-179.71(9)
C7-S1-C10-C9	-0.98(8)	C6-C1-C2-H2	-179.63
C10-S1-C7-C8	1.03(7)	C6-C1-C2-C3	0.4(1)
C10-S1-C7-C1	-178.40(7)	C7-C1-C6-C5	179.72(9)
H1C-O1-C11-C9	-167(1)	C7-C1-C6-H6	-0.3
H1C-O1-C11-C13	-44(1)	C2-C1-C6-C5	-0.4(1)
H1C-O1-C11-C12	73(1)	C2-C1-C6-H6	179.65
C15-N2-C13-C18	2.0(1)	C4-C5-C6-C1	0.4(2)
C15-N2-C13-C11	-178.87(8)	C4-C5-C6-H6	-179.7
C13-N2-C15-H15	177.02	H5-C5-C6-C1	-179.65
C13-N2-C15-C16	-3.0(2)	H5-C5-C6-H6	0.3
H1A-N1-C10-S1	160(1)	H18-C18-C13-N2	-179.6
H1A-N1-C10-C9	-23(1)	H18-C18-C13-C11	1.3
H1B-N1-C10-S1	9(2)	C17-C18-C13-N2	0.4(1)
H1B-N1-C10-C9	-174(2)	C17-C18-C13-C11	-178.71(9)
C10-C9-C8-H8	-179.87	H18-C18-C17-H17	-1.9
C10-C9-C8-C7	0.1(1)	H18-C18-C17-C16	178.1
C11-C9-C8-H8	5.2	C13-C18-C17-H17	178.1
C11-C9-C8-C7	-174.79(8)	C13-C18-C17-C16	-1.8(2)
C8-C9-C10-S1	0.7(1)	N2-C13-C11-O1	25.3(1)
C8-C9-C10-N1	-177.2(1)	N2-C13-C11-C9	145.98(8)
C11-C9-C10-S1	175.91(7)	N2-C13-C11-C12	-90.52(9)
C11-C9-C10-N1	-1.9(2)	C18-C13-C11-O1	-155.56(8)
C8-C9-C11-O1	-142.52(9)	C18-C13-C11-C9	-34.9(1)
C8-C9-C11-C13	95.3(1)	C18-C13-C11-C12	88.6(1)
C8-C9-C11-C12	-26.1(1)	O1-C11-C12-F3	-172.68(7)
C10-C9-C11-O1	43.0(1)	O1-C11-C12-F1	66.4(1)
C10-C9-C11-C13	-79.2(1)	O1-C11-C12-F2	-53.7(1)
C10-C9-C11-C12	159.40(8)	C9-C11-C12-F3	69.6(1)
C9-C8-C7-S1	-0.8(1)	C9-C11-C12-F1	-51.3(1)
C9-C8-C7-C1	178.52(9)	C9-C11-C12-F2	-171.42(8)
H8-C8-C7-S1	179.15	C13-C11-C12-F3	-53.8(1)
H8-C8-C7-C1	-1.5	C13-C11-C12-F1	-174.69(8)
H4-C4-C5-H5	-0.3	C13-C11-C12-F2	65.2(1)
H4-C4-C5-C6	179.7	C1-C2-C3-C4	-0.4(2)
C3-C4-C5-H5	179.7	C1-C2-C3-H3	179.62
C3-C4-C5-C6	-0.4(2)	H2-C2-C3-C4	179.6
H4-C4-C3-C2	-179.6	H2-C2-C3-H3	-0.4
H4-C4-C3-H3	0.3	C18-C17-C16-C15	0.9(2)
C5-C4-C3-C2	0.4(2)	C18-C17-C16-H16	-179.1
C5-C4-C3-H3	-179.6	H17-C17-C16-C15	-179
S1-C7-C1-C2	173.72(7)	H17-C17-C16-H16	0.9

S1-C7-C1-C6	-6.4(1)	N2-C15-C16-C17	1.5(2)
C8-C7-C1-C2	-5.6(1)	N2-C15-C16-H16	-178.5
C8-C7-C1-C6	174.33(9)	H15-C15-C16-C17	-178.5
C7-C1-C2-H2	0.3	H15-C15-C16-H16	1.5

Table S9 Hydrogen bond distances (Å) and angles for **12j** (°).

	**Donor-H**	**Acceptor-H**	**Donor-Acceptor**	**Angle**
N1-H1A···O1[a]	0.843	2.187	2.800	129
O1-H1C···N2[b]	0.830	2.237	2.899	137

[a]intramolecular interaction
[b]intermolecular interaction

References

1. Boibessot, T.; Zschiedrich, C. P.; Dunyach-Rémy, C.; Lebeau, A.; Bénimèlis, D.; Dunyach-Rémy, C.; Lavigne, J.-P.; Szurmant, H.; Benfodda, Z.; Meffre, P. The Rational Design, Synthesis, and Antimicrobial Properties of Thiophene Derivatives That Inhibit Bacterial Histidine Kinases. *J. Med. Chem.* **2016**, *59* (19), 8830–8847. https://doi.org/10.1021/acs.jmedchem.6b00580.
2. Nguyen, T.; Gamage, T. F.; Decker, A. M.; Barrus, D.; Langston, T. L.; Li, J. X.; Thomas, B. F.; Zhang, Y. Synthesis and Pharmacological Evaluation of 1-Phenyl-3-Thiophenylurea Derivatives as Cannabinoid Type-1 Receptor Allosteric Modulators. *J. Med. Chem.* **2019**, *62* (21), 9806–9823. https://doi.org/10.1021/acs.jmedchem.9b01161.
3. Krause, L.; Herbst-Irmer, R.; Sheldrick, G. M.; Stalke, D. SADABS Version 2016/2. 2015, pp 3–10.
4. Sheldrick, G. Crystal Structure Refinement with SHELXL. *Acta Crystallogr. Sect. C* **2015**, *71*. https://doi.org/10.1107/S2053229614024218.

Characterization of compounds (^{1}H 300MHz, ^{13}C 75MHz, ^{19}F NMR 376 MHz, DEPT-135 in DMSO-d_6; HRMS, HPLC)

2-nitro-5-phenylthiophene (7)

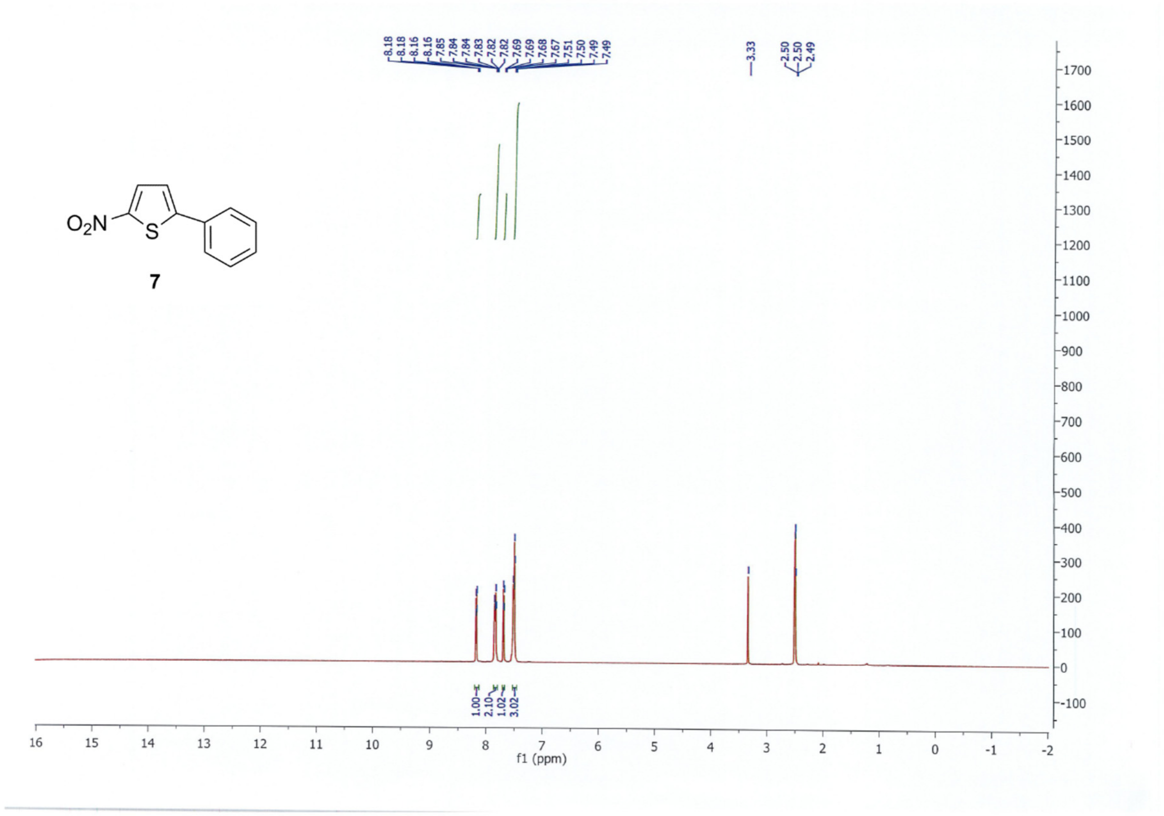

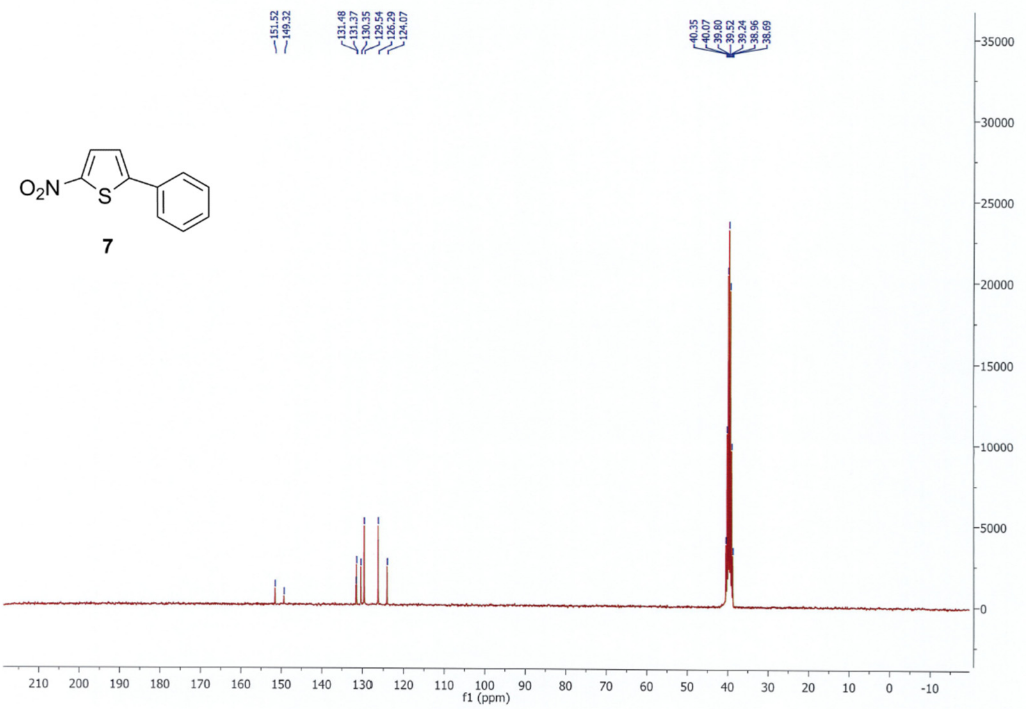
151.52
149.32
131.48
131.37
130.35
129.54
126.29
124.07
40.35
40.07
39.80
39.52
39.24
38.96
38.69
O2N
S
7
f1 (ppm)

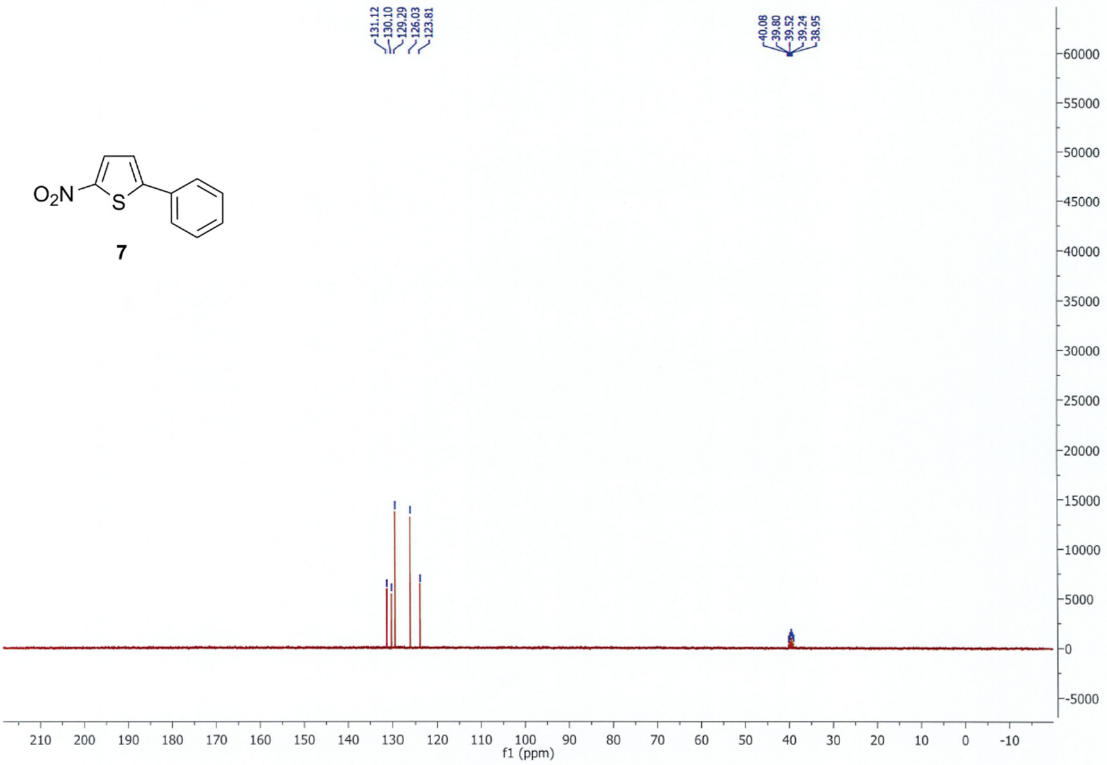
131.12
130.10
129.29
126.03
123.81
40.08
39.80
39.52
39.24
38.95
O2N
S
7
f1 (ppm)

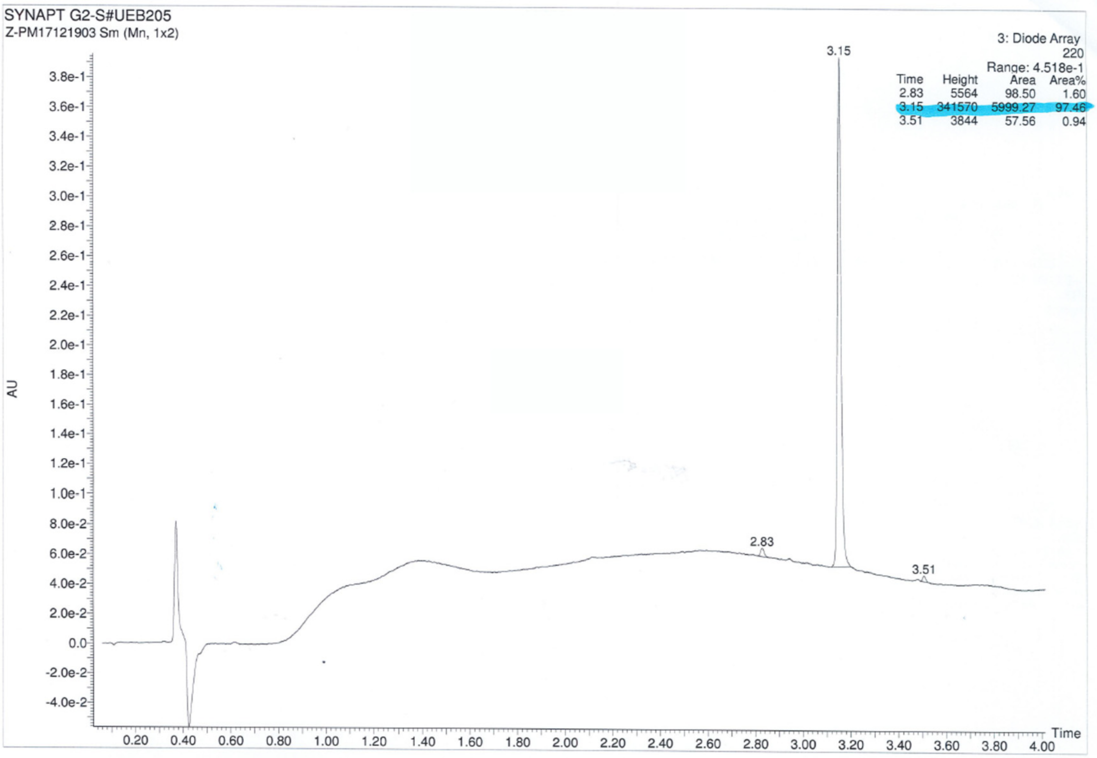

Elemental Composition Report — Page 1

Single Mass Analysis
Tolerance = 1.0 mDa / DBE: min = -1.5, max = 100.0
Element prediction: Off
Number of isotope peaks used for i-FIT = 3

Monoisotopic Mass, Even Electron Ions
495 formula(e) evaluated with 4 results within limits (up to 50 best isotopic matches for each mass)
Elements Used:
C: 0-100 H: 0-100 N: 0-20 O: 0-20 S: 0-2
SYNAPT G2-S#UEB205
Z-PM17121903 798 (3.152) Cm (796:811-(756:771+821:842))

20-Dec-2017
1: TOF MS ES+
7.01e+004

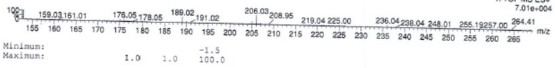

Minimum:				-1.5				
Maximum:		1.0	1.0	100.0				
Mass	Calc. Mass	mDa	PPM	DBE	i-FIT	Norm	Conf(%)	Formula
206.0276	206.0276	0.0	0.0	7.5	127.9	0.000	99.99	C10 H8 N O2 S
	206.0283	-0.7	-3.4	3.5	137.8	9.917	0.00	C3 H8 N7 S2
	206.0269	0.7	3.4	-1.5	137.9	9.996	0.00	C2 H12 N3 O4 S2
	206.0274	0.2	1.0	4.5	145.0	17.165	0.00	C2 H4 N7 O5

*5-phenylthiophen-2-amine (**8**)*

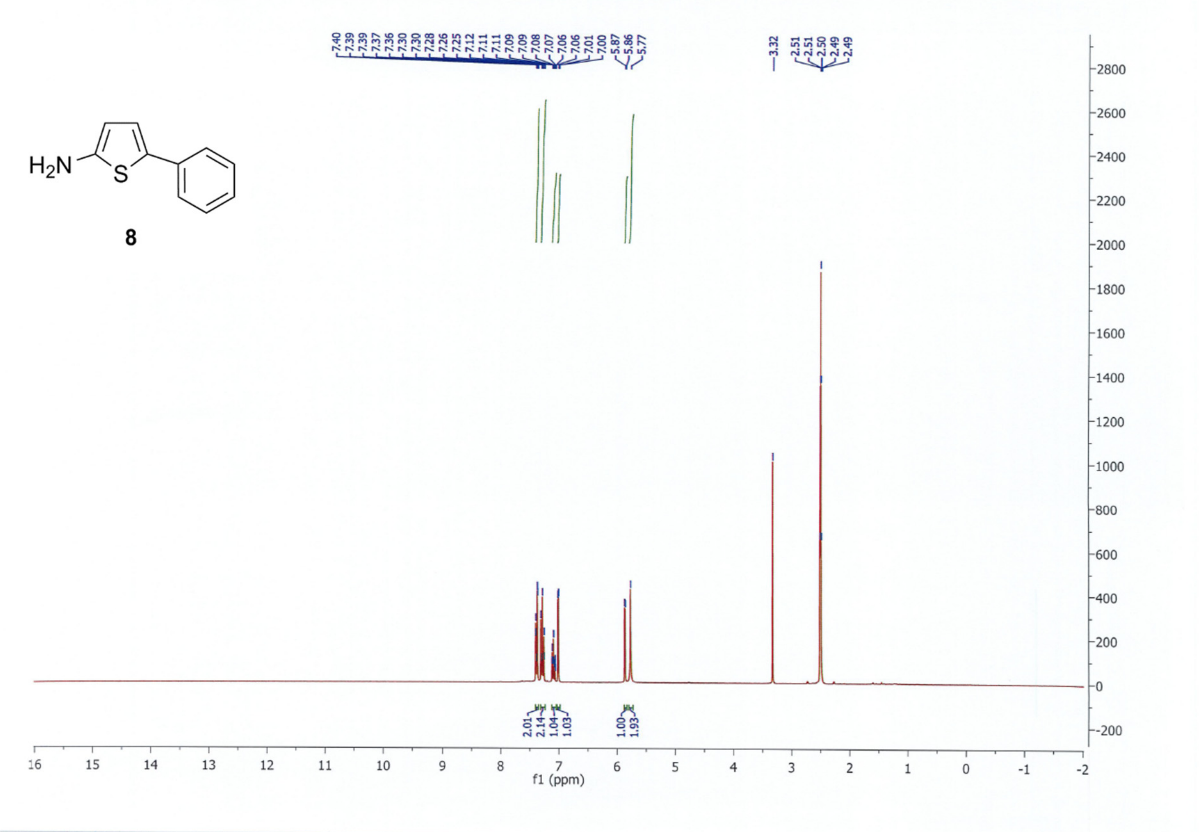

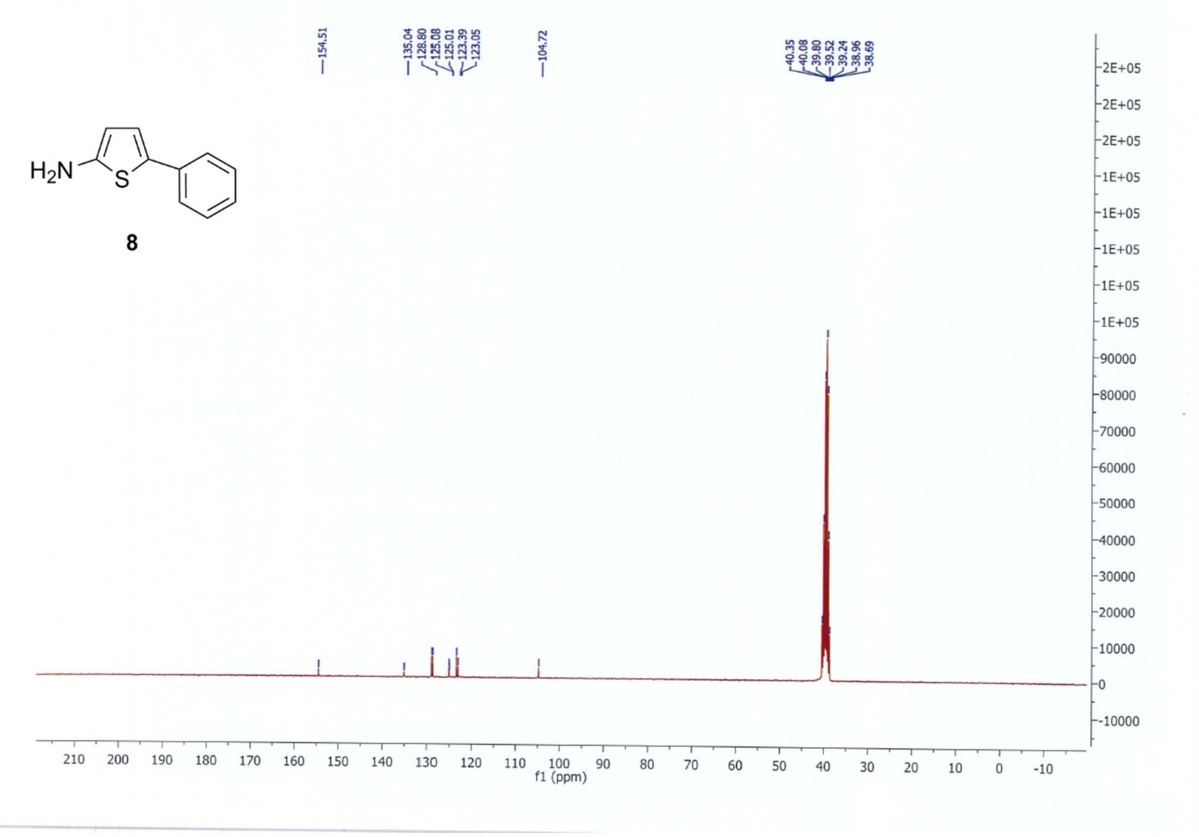

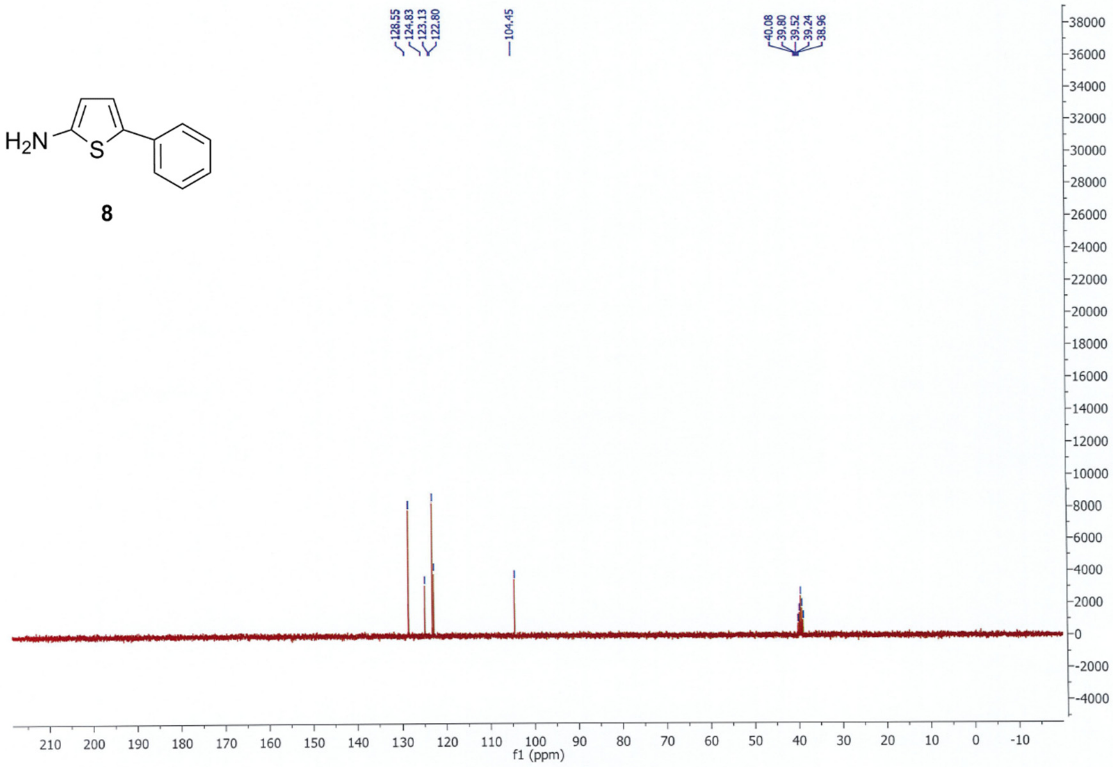

8

High Resolution Mass Result

**Analysis Info**		Acquisition Date	5/10/2021 2:49:16 PM	
Sample Name	**DUV-48 _ VD-MRH-42**	Instrument / Ser#	micrOTOF-Q	228888.10300

Acquisition Parameter

Source Type	ESI	Ion Polarity	Positive	Scan Begin	50 m/z	Scan End	2200 m/z

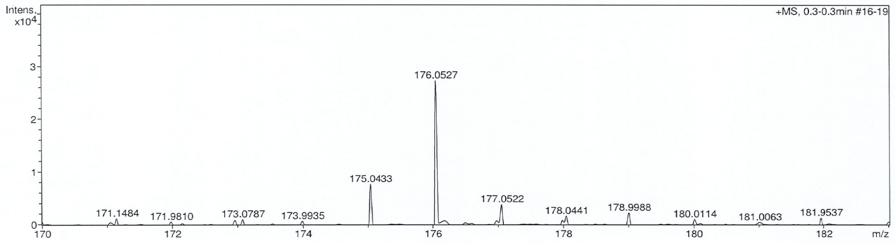

Meas. m/z	#	Ion Formula	m/z	err [ppm]	mSigma	# Sigma	Score	rdb	e⁻ Conf	N-Rule
176.0527	1	C10H10NS	176.0528	-1.0	11.4	1	100.00	6.5	even	ok

Bruker Compass DataAnalysis 4.1 printed: 8/31/2021 11:02:02 AM Page 1 of 1

*2-(2-amino-5-phenylthiophen-3-yl)-1,1,1-trifluoropropan-2-ol (**12a**)*

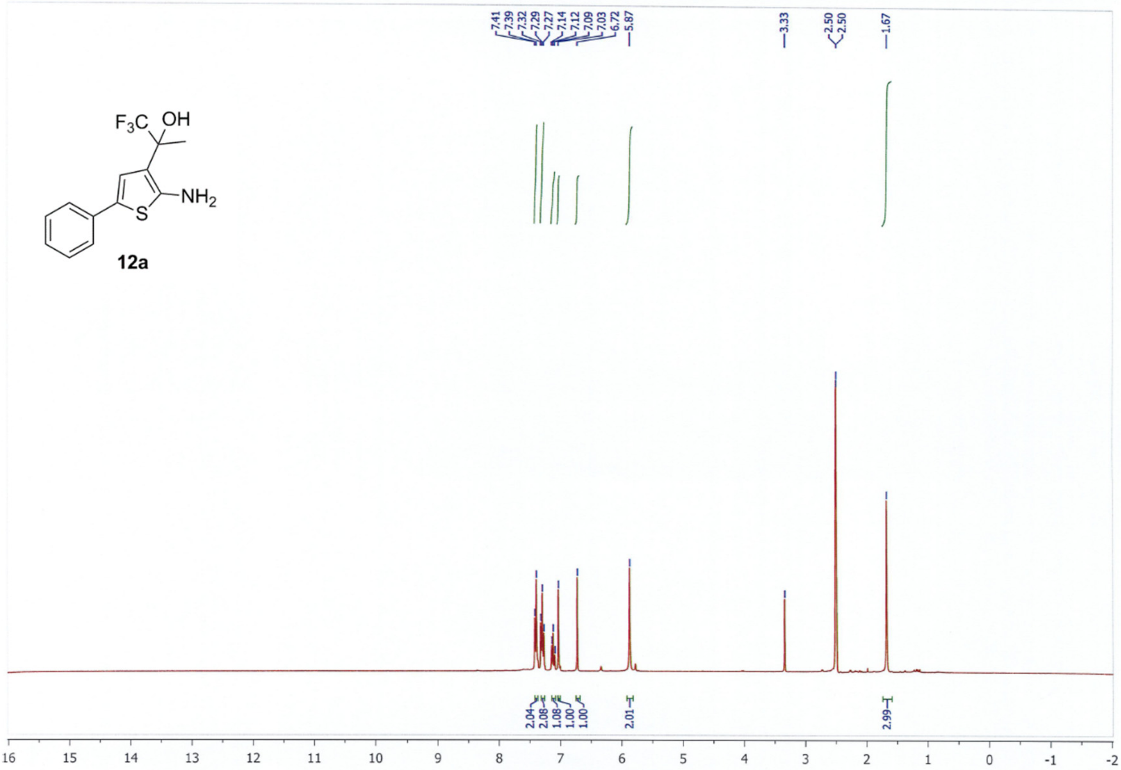

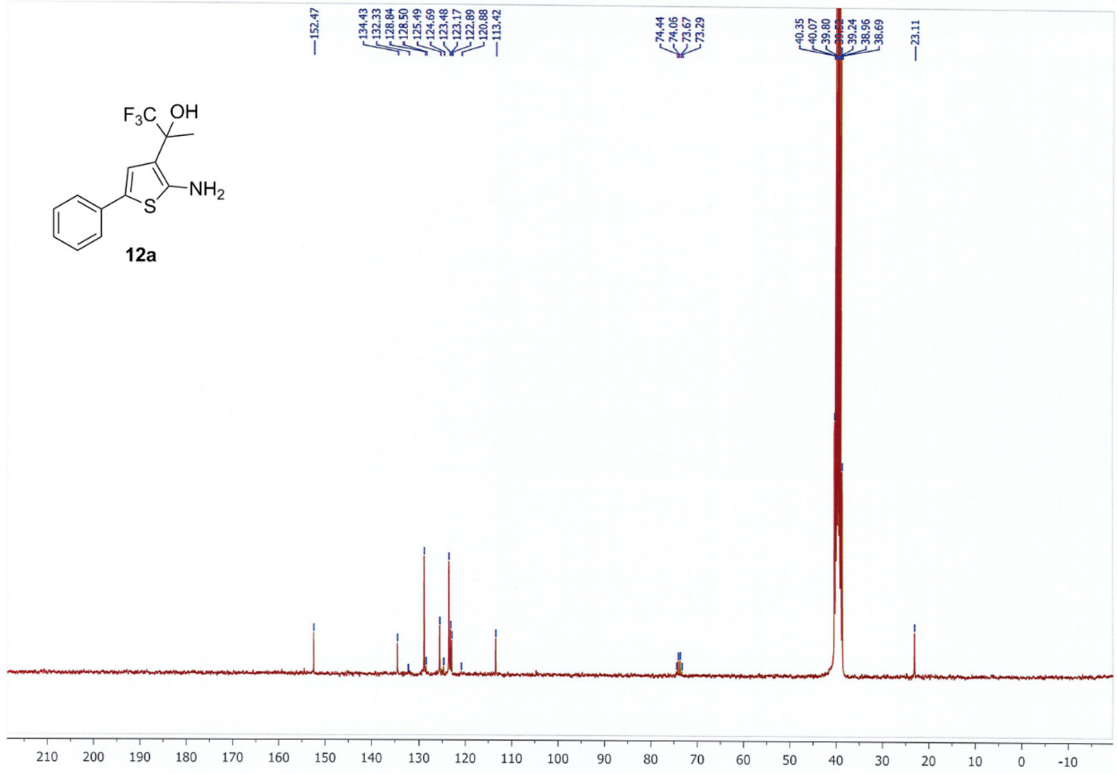

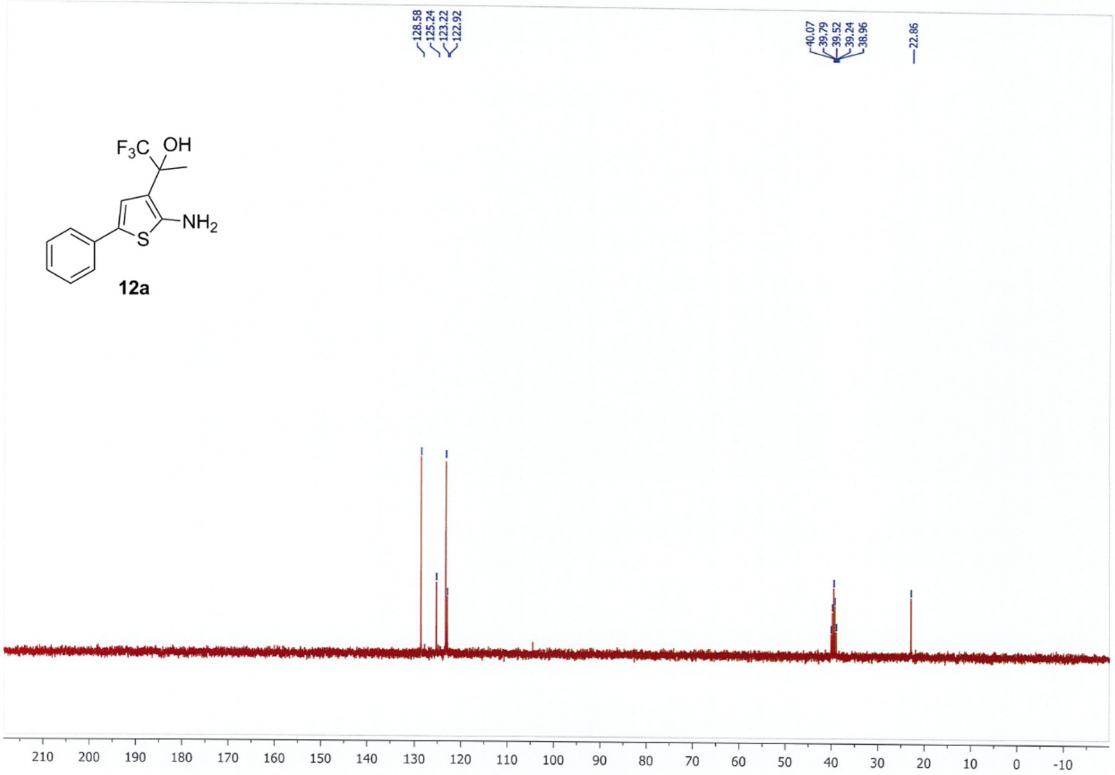

128.58
125.24
123.22
122.92
40.07
39.79
39.52
39.24
38.96
22.86
F3C OH
NH2
S
210 200 190 180 170 160 150 140 130 120 110 100 90 80 70 60 50 40 30 20 10 0 -10

12a

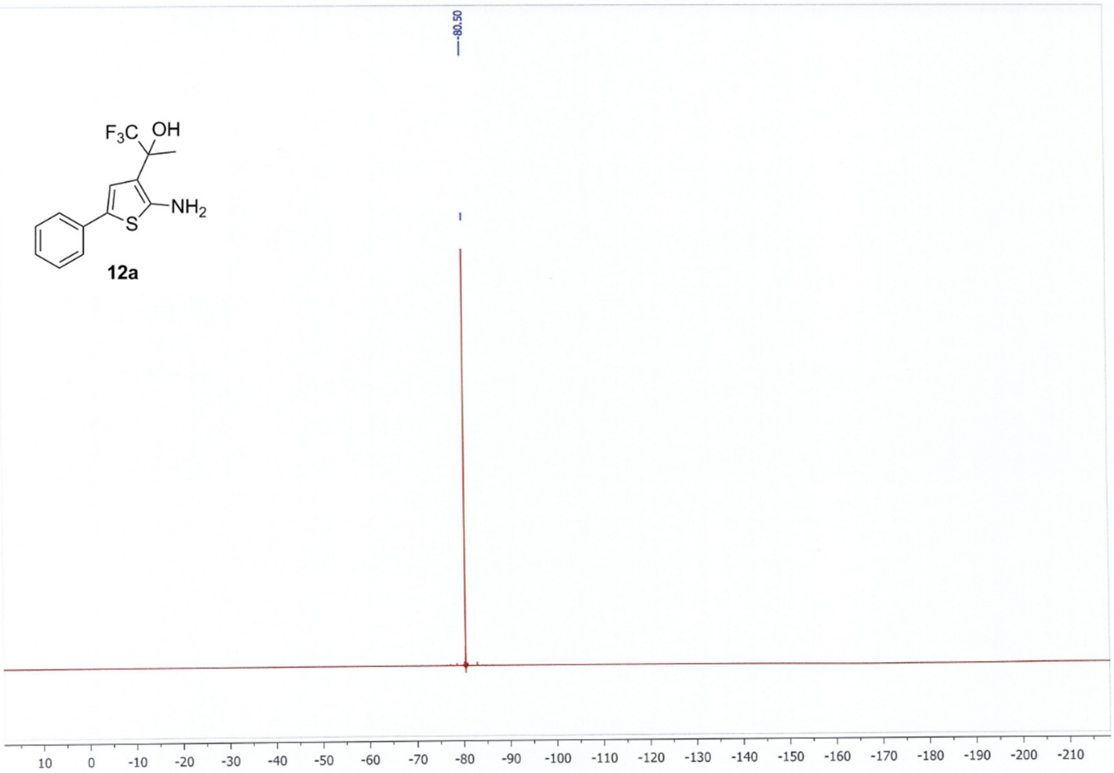

-80.50
F3C OH
NH2
S
10 0 -10 -20 -30 -40 -50 -60 -70 -80 -90 -100 -110 -120 -130 -140 -150 -160 -170 -180 -190 -200 -210

12a

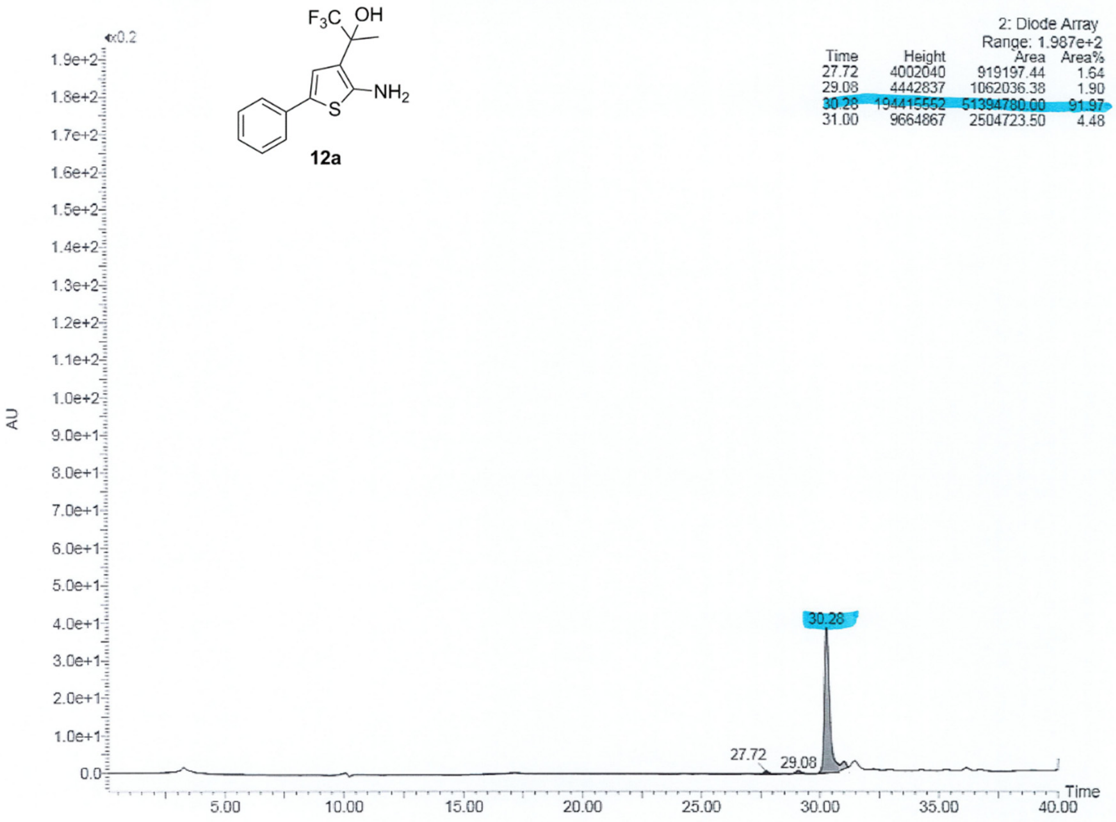

Elemental Composition Report

Page 1

Single Mass Analysis

Tolerance = 3.0 mDa / DBE: min = -1.0, max = 100.0
Element prediction: Off
Number of isotope peaks used for i-FIT = 3

Monoisotopic Mass, Even Electron Ions
196 formula(e) evaluated with 1 results within limits (up to 50 best isotopic matches for each mass)
Elements Used:
C: 0-100 H: 0-100 N: 0-20 O: 0-30 S: 1-1 F: 3-3

SYNAPT G2-S#NotSet VD-MRH-62 02-Feb-2021
CHR-2_3 12 (0.211) Cm (11:14) 1: TOF MS ES+ 1.57e+005

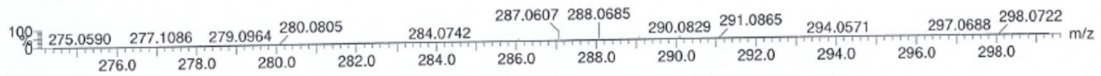

Minimum:				-1.0				
Maximum:		3.0	3.0	100.0				
Mass	Calc. Mass	mDa	PPM	DBE	i-FIT	Norm	Conf(%)	Formula
288.0685	288.0670	1.5	5.2	6.5	2373.7	n/a	n/a	C13 H13 N O S F3

*2-(2-amino-5-phenylthiophen-3-yl)-1,1,1-trifluoro-3-phenylpropan-2-ol (**12b**)*

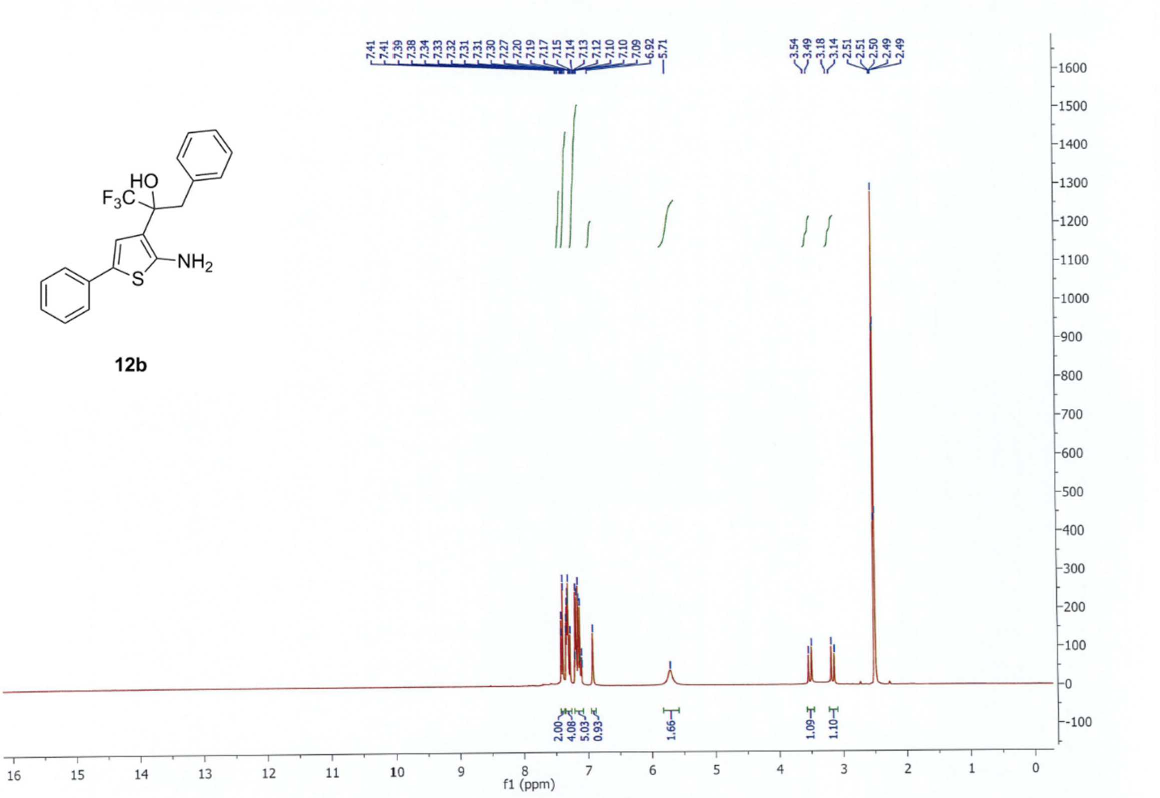

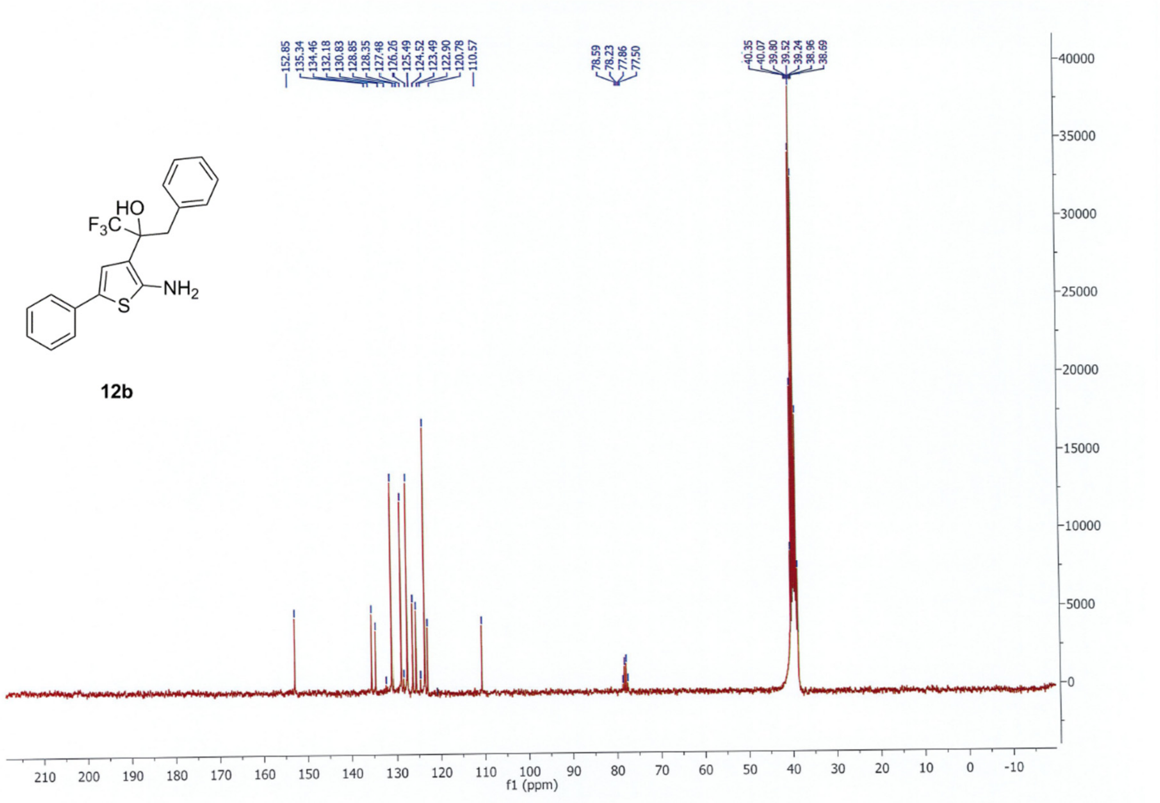

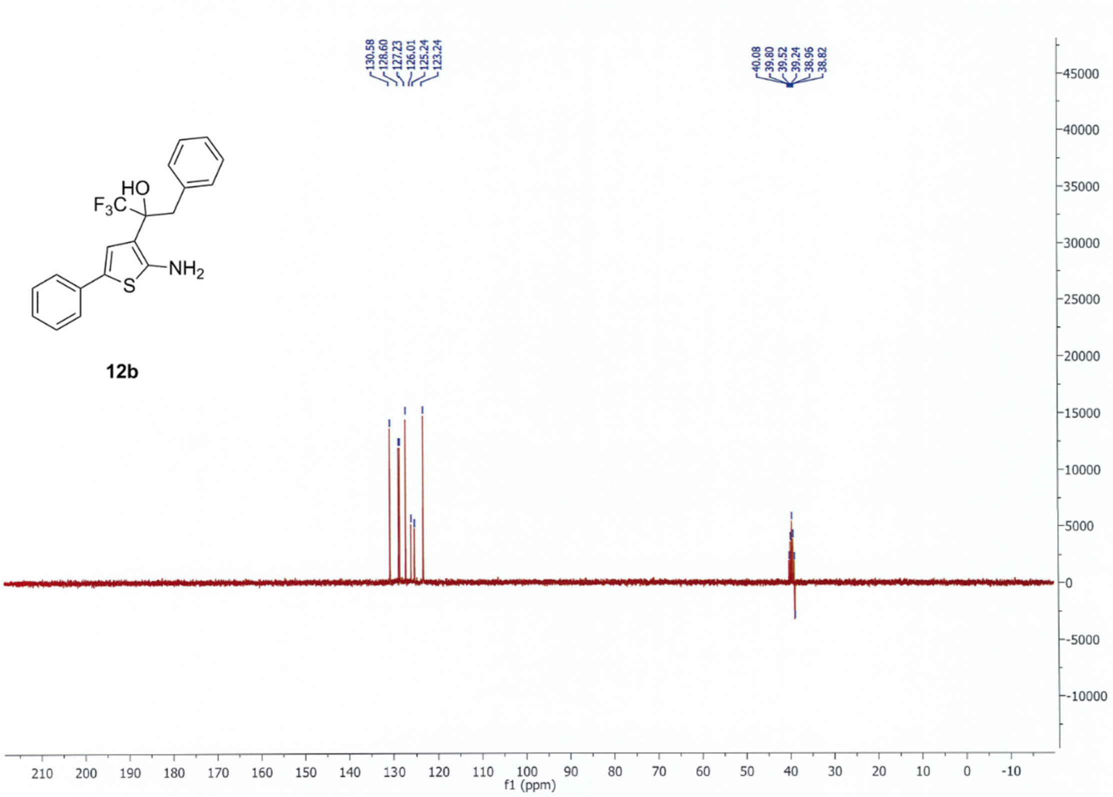
130.58
128.60
127.23
126.01
125.24
123.24
40.08
39.80
39.52
39.24
38.96
38.82
HO
F3C
NH2
S
12b
f1 (ppm)

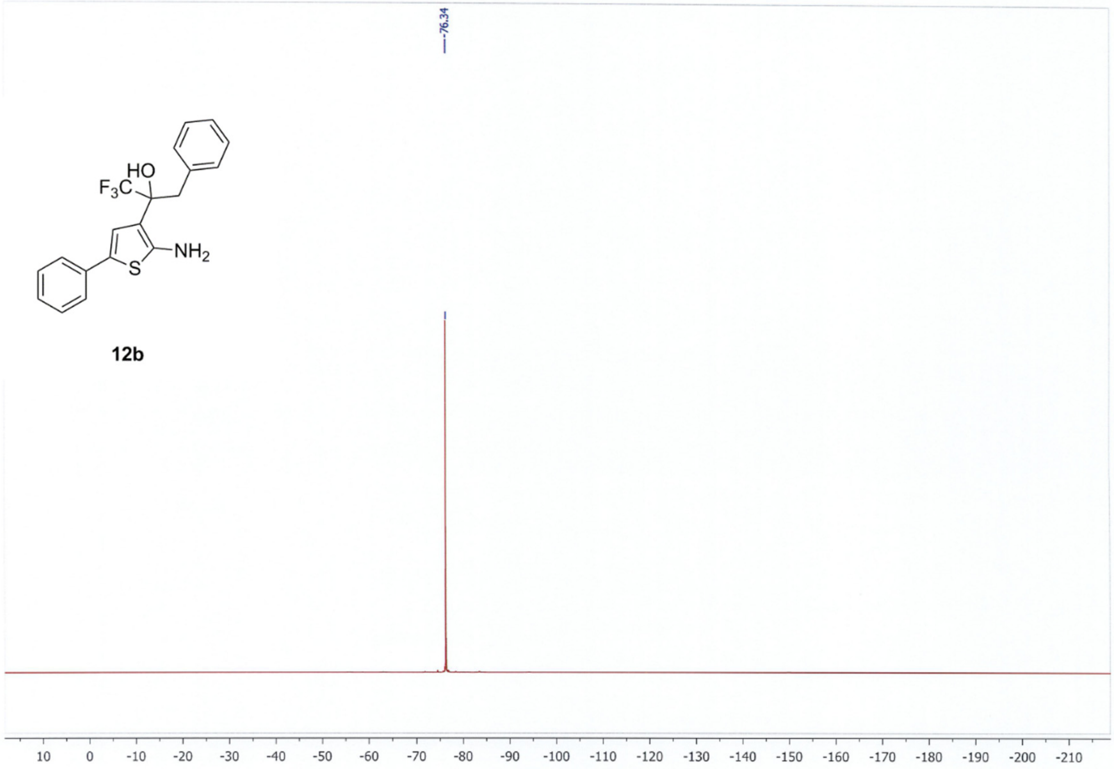
-76.34
HO
F3C
NH2
S
12b

VD-MRH-97-2 Sm (Mn, 5x1)

2: Diode Array
Range: 4.253e+2

Time	Height	Area	Area%
30.72	4716817	1315132.50	0.80
32.13	418473472	138531424.00	83.93
34.75	22003112	20600436.00	12.48
37.18	4745740	1553817.25	0.94
38.75	12351343	3048280.50	1.85

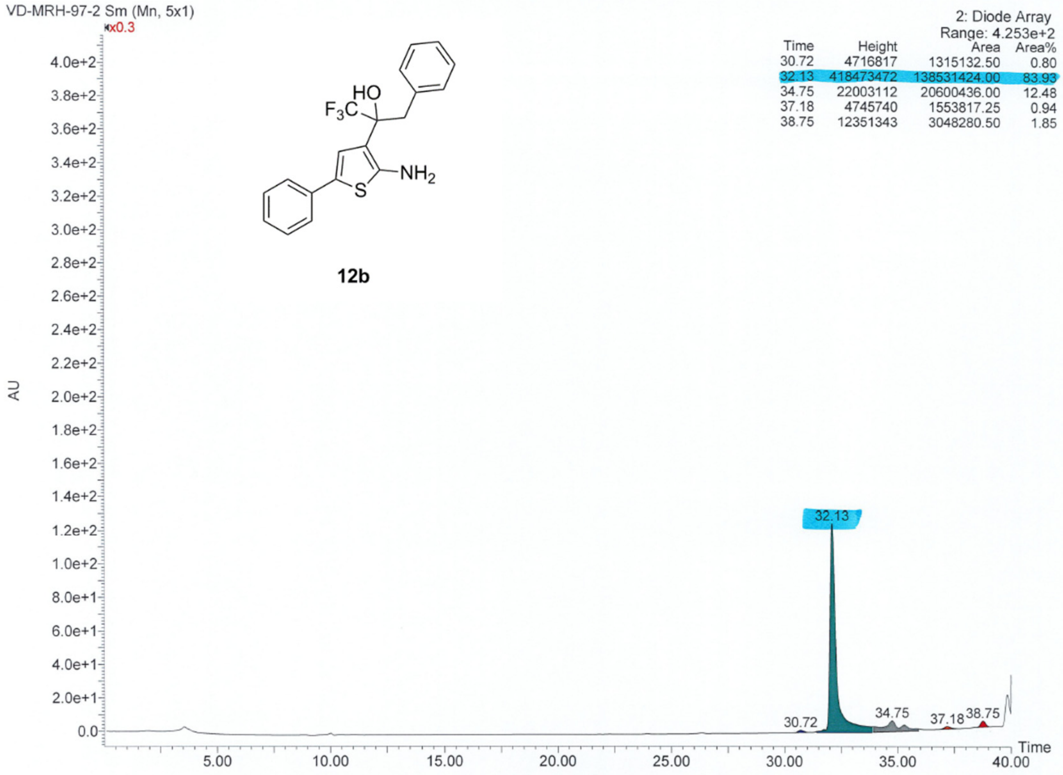

12b

High Resolution Mass Result

Analysis Info

Sample Name **VD-MRH-97**

Acquisition Date 5/10/2021 2:55:21 PM

Instrument / Ser# micrOTOF-Q 228888.10300

Acquisition Parameter

Source Type ESI | Ion Polarity Positive | Scan Begin 50 m/z | Scan End 2200 m/z

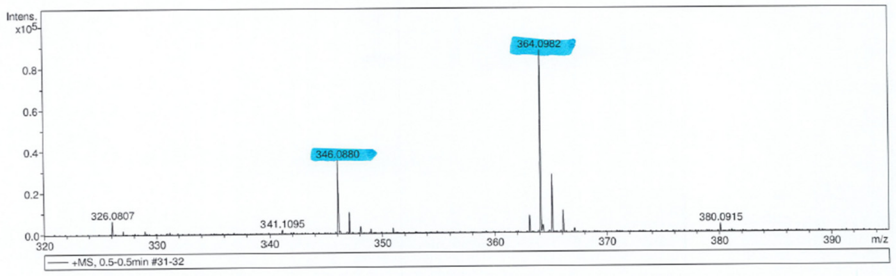

Meas. m/z	#	Ion Formula	m/z	err [ppm]	mSigma	# Sigma	Score	rdb	e⁻ Conf	N-Rule
346.0880	1	C19H15F3NS	346.0872	-2.3	38.7	1	100.00	11.5	even	ok
364.0982	1	C19H17F3NOS	364.0977	-1.2	55.9	1	100.00	10.5	even	ok
	2	C8H17F3N7O4S	364.1009	-7.6	102.2	2	3.12	2.5	even	ok
	3	C7H21F3N3O8S	364.0996	3.9	112.7	3	5.25	-2.5	even	ok

Bruker Compass DataAnalysis 4.1 | printed: 5/10/2021 3:47:57 PM | Page 1 of 1

*1-(2-amino-5-phenylthiophen-3-yl)-2,2,2-trifluoro-1-(furan-2-yl)ethan-1-ol (**12c**)*

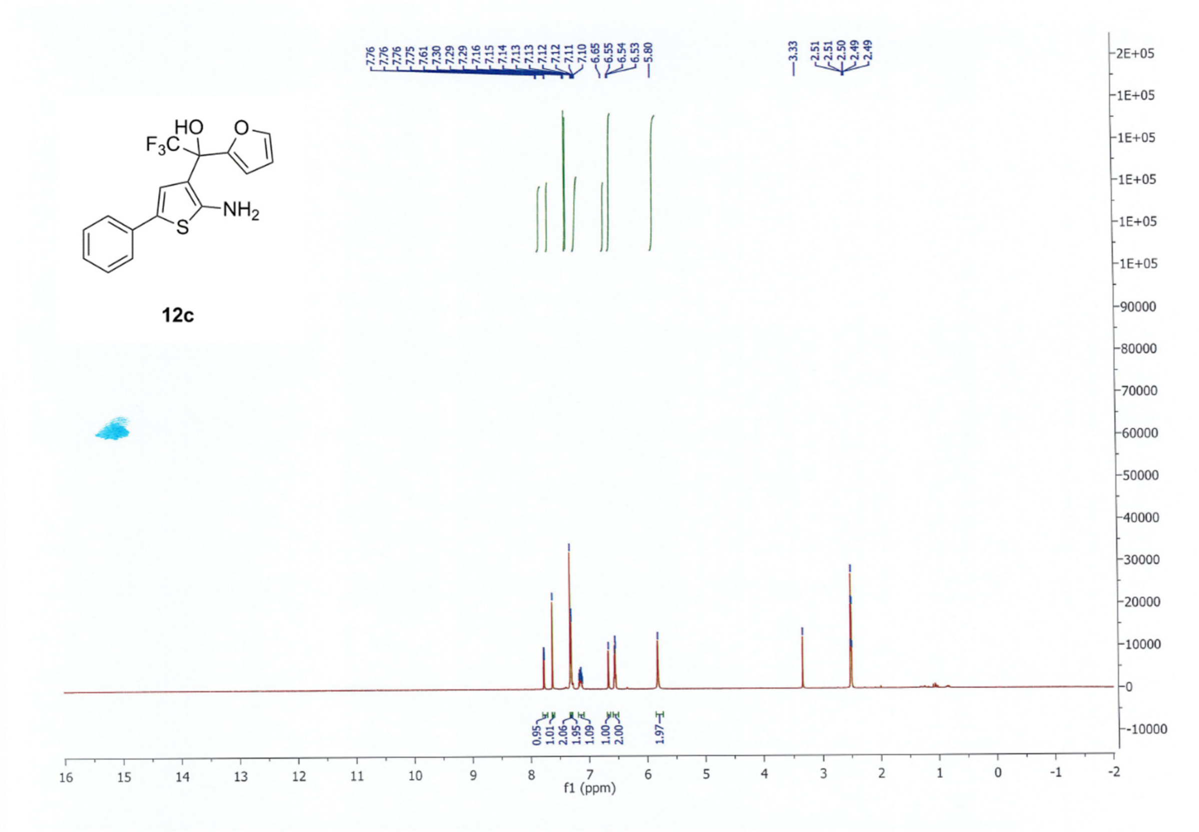

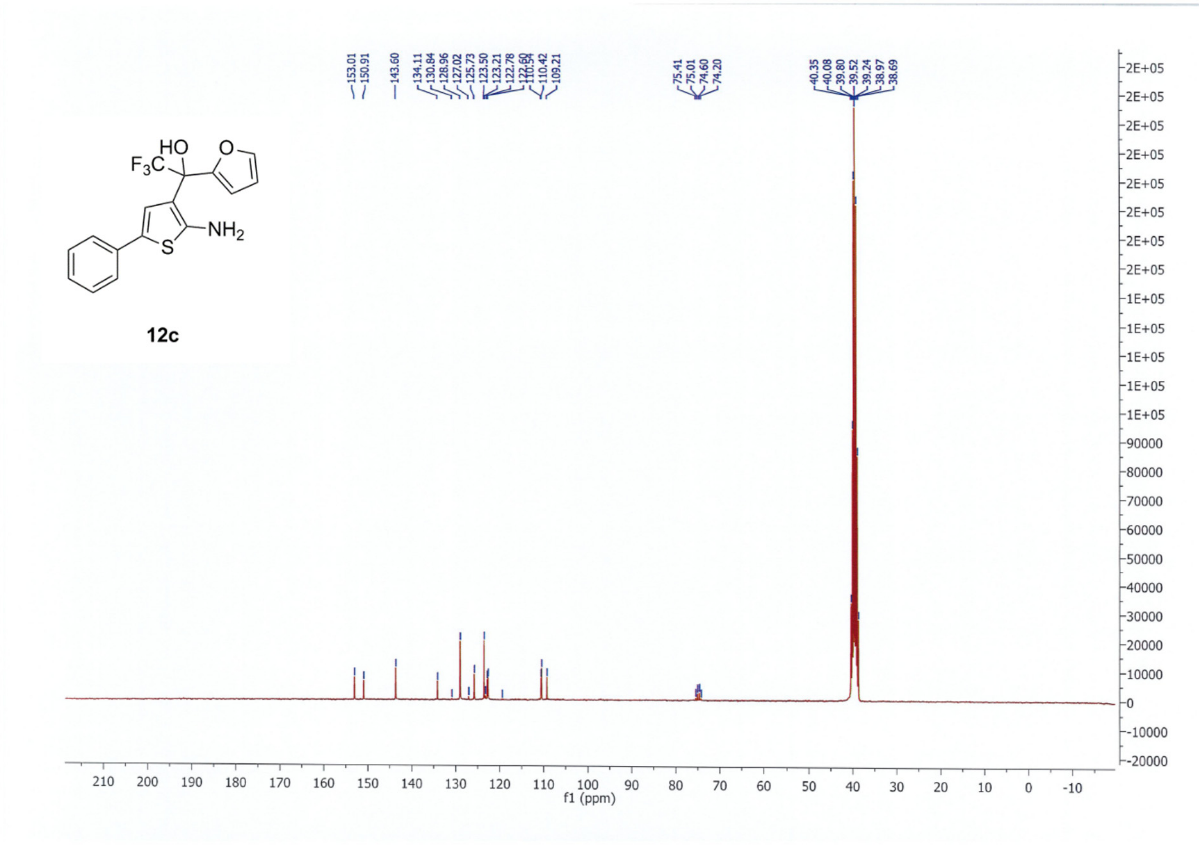

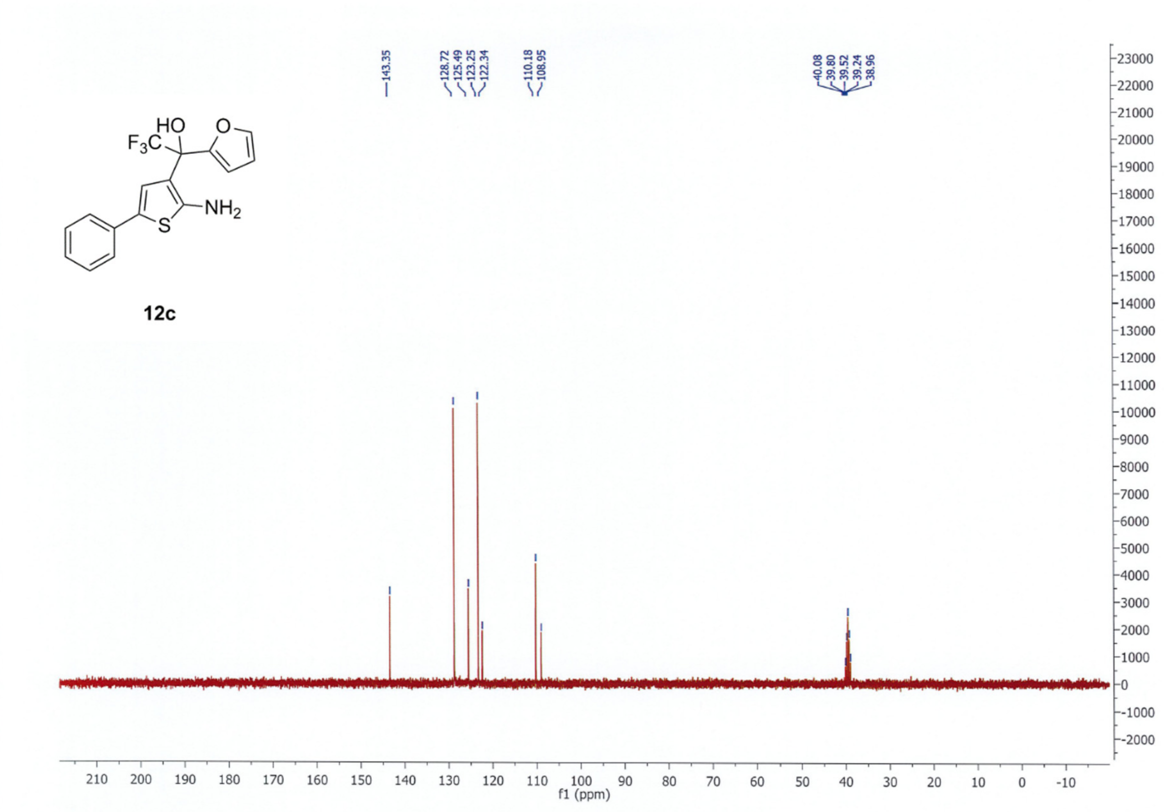
143.35
128.72
125.49
123.25
122.34
110.18
108.95
40.08
39.80
39.52
39.24
38.96
HO
O
F_3C
NH_2
S
12c
f1 (ppm)

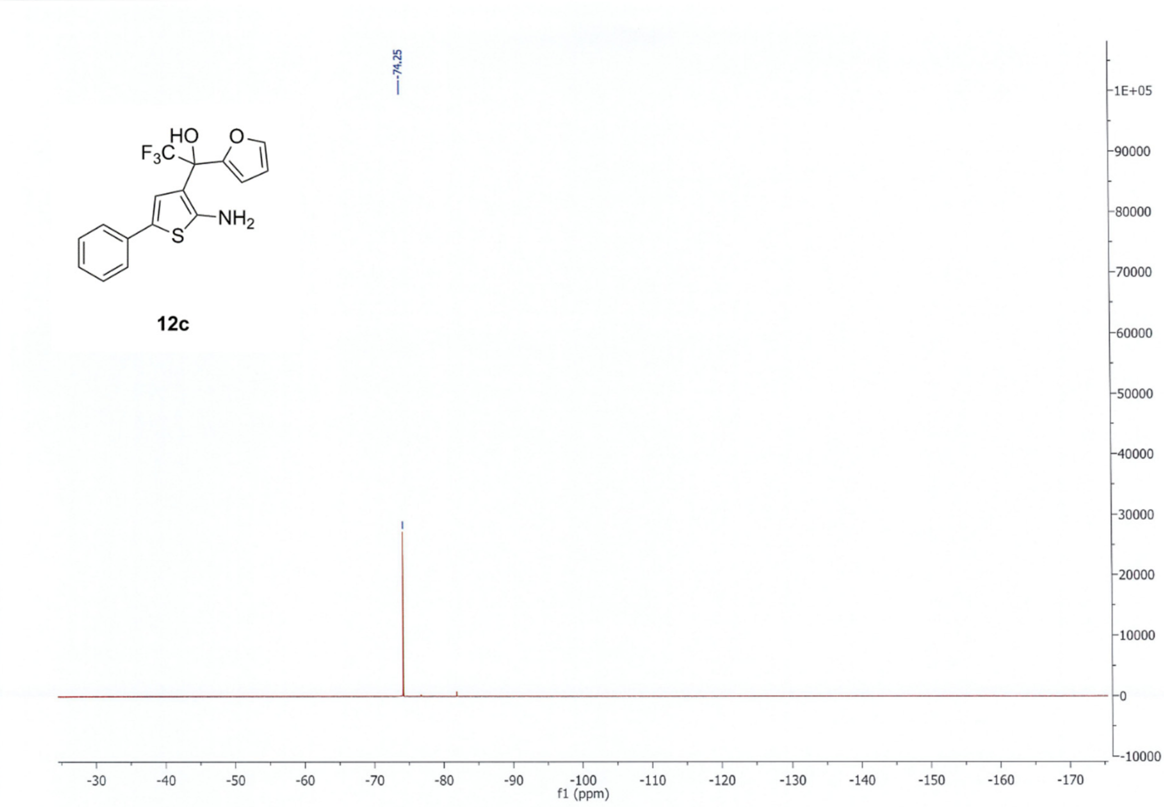
-74.25
HO
O
F_3C
NH_2
S
12c
f1 (ppm)

High Resolution Mass Result

**Analysis Info**		Acquisition Date	7/23/2021 7:30:20 PM
Sample Name	**DUV-47 _ VD-MRH-109**	Instrument / Ser#	micrOTOF-Q 228888.10300

Acquisition Parameter
Source Type ESI   Ion Polarity Positive   Scan Begin 50 m/z   Scan End 2200 m/z

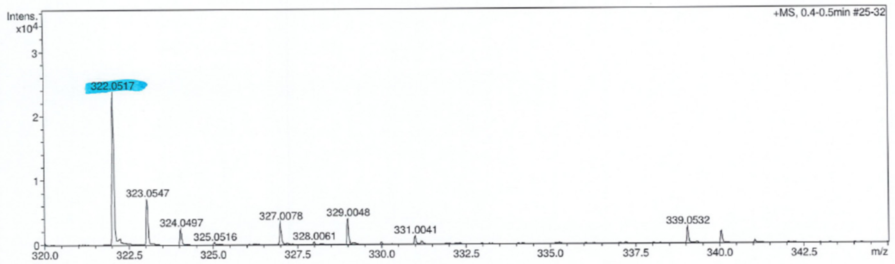

Meas. m/z	#	Ion Formula	m/z	err [ppm]	mSigma	# Sigma	Score	rdb	e⁻ Conf	N-Rule
322.0517	1	C16H11F3NOS	322.0508	2.7	61.1	1	100.00	10.5	even	ok
	2	C8H8F3N8OS	321.0488	3.7	676.0	2	0.00	7.5	even	ok
	3	C9H9F3N7OS	320.0536	2.5	702.2	3	0.00	7.5	even	ok
339.0532	1	C16H12F3NO2S	339.0535	-0.9	288.7	1	100.00	10.0	odd	ok
	2	C8H9F3N8O2S	338.0516	0.0	733.8	2	0.00	7.0	odd	ok
	3	C7H13F3N4O6S	338.0502	2.4	739.6	3	0.00	2.0	odd	ok
	4	C8H14F3N3O6S	337.0550	-0.0	758.5	4	0.00	2.0	odd	ok
	5	C9H10F3N7O2S	337.0563	-1.9	760.5	5	0.00	7.0	odd	ok
340.0606	1	C16H13F3NO2S	340.0614	-2.3	40.8	1	100.00	9.5	even	ok

Bruker Compass DataAnalysis 4.1   printed: 7/26/2021 10:32:48 AM   Page 1 of 1

VD-MRH-61 Sm (Mn, 5x1)

2: Diode Array
Range: 3.423e+1

Time	Height	Area	Area%
26.27	4124905	738595.81	7.32
30.53	2583238	668916.38	6.63
32.27	2415173	606676.38	6.01
32.93	30593302	8073059.50	80.03

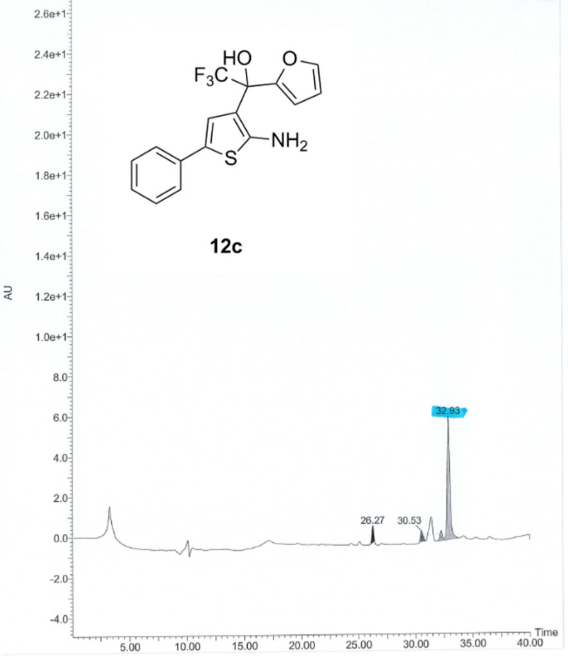

12c

*1-(2-amino-5-phenylthiophen-3-yl)-2,2,2-trifluoro-1-(thiophen-2-yl)ethan-1-ol (**12d**)*

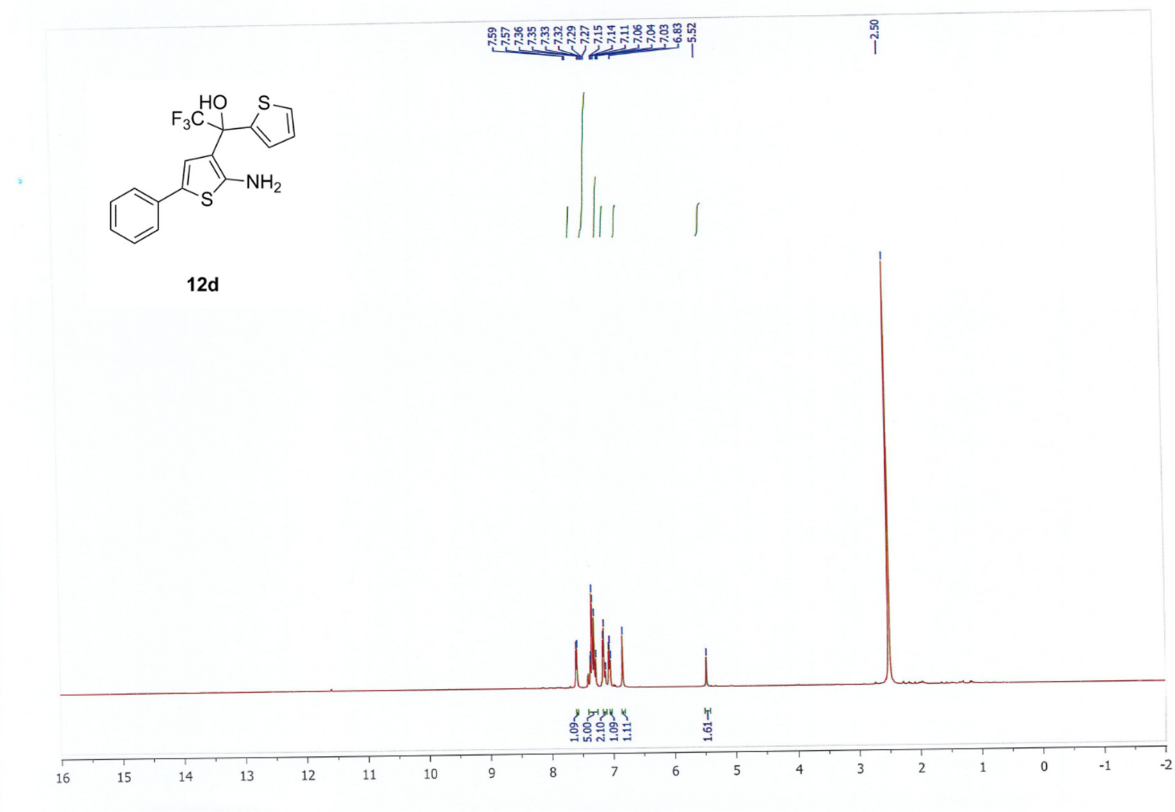

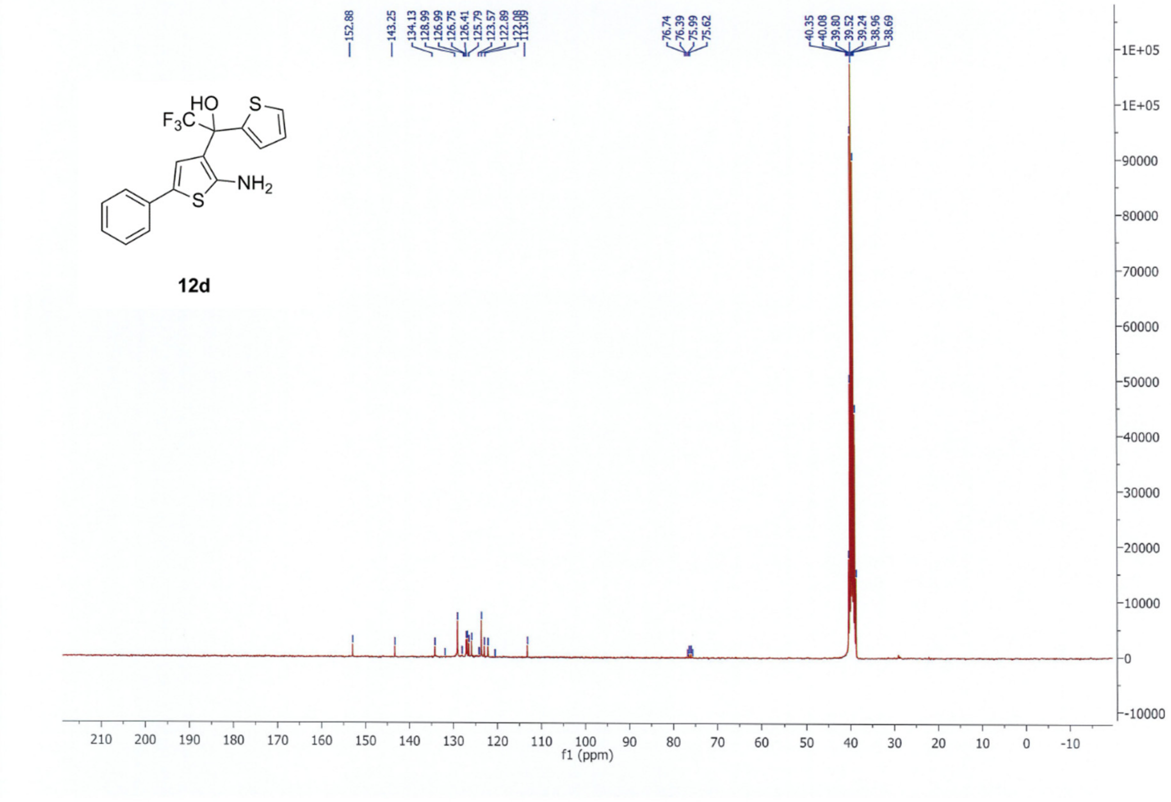

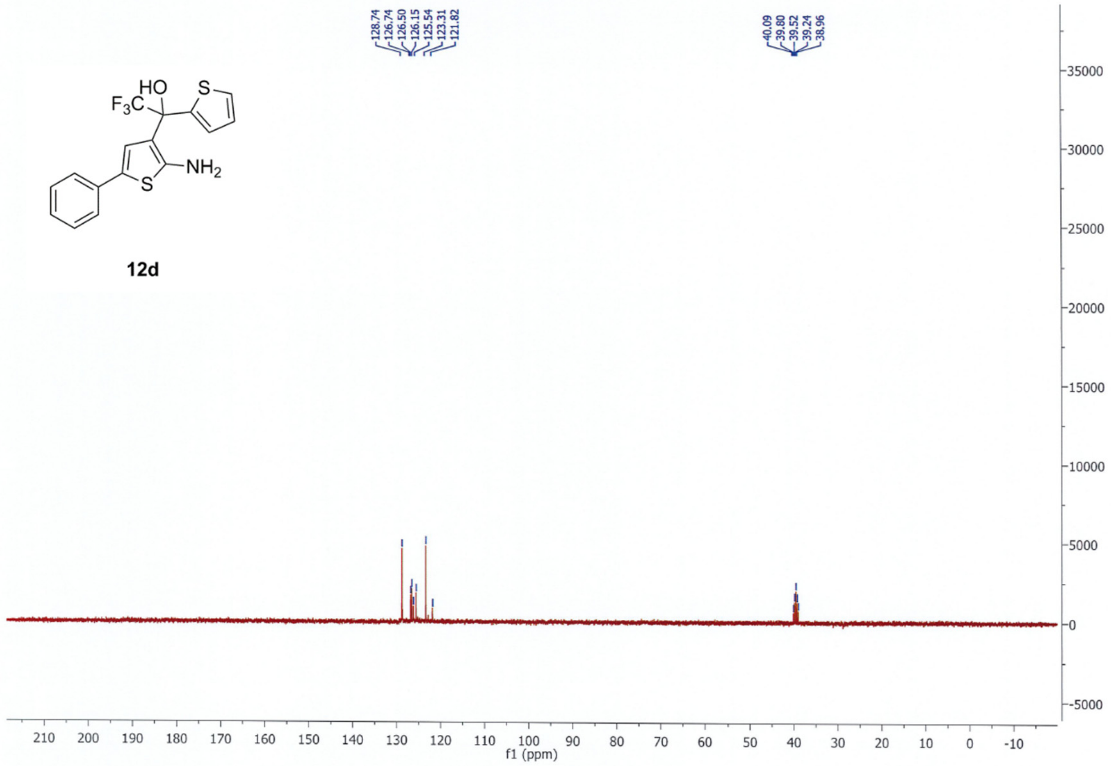
128.74
126.74
126.50
126.15
125.54
123.31
121.82
40.09
39.80
39.52
39.24
38.96
HO
F3C
S
NH2
12d
35000
30000
25000
20000
15000
10000
5000
0
-5000
210 200 190 180 170 160 150 140 130 120 110 100 90 80 70 60 50 40 30 20 10 0 -10
f1 (ppm)

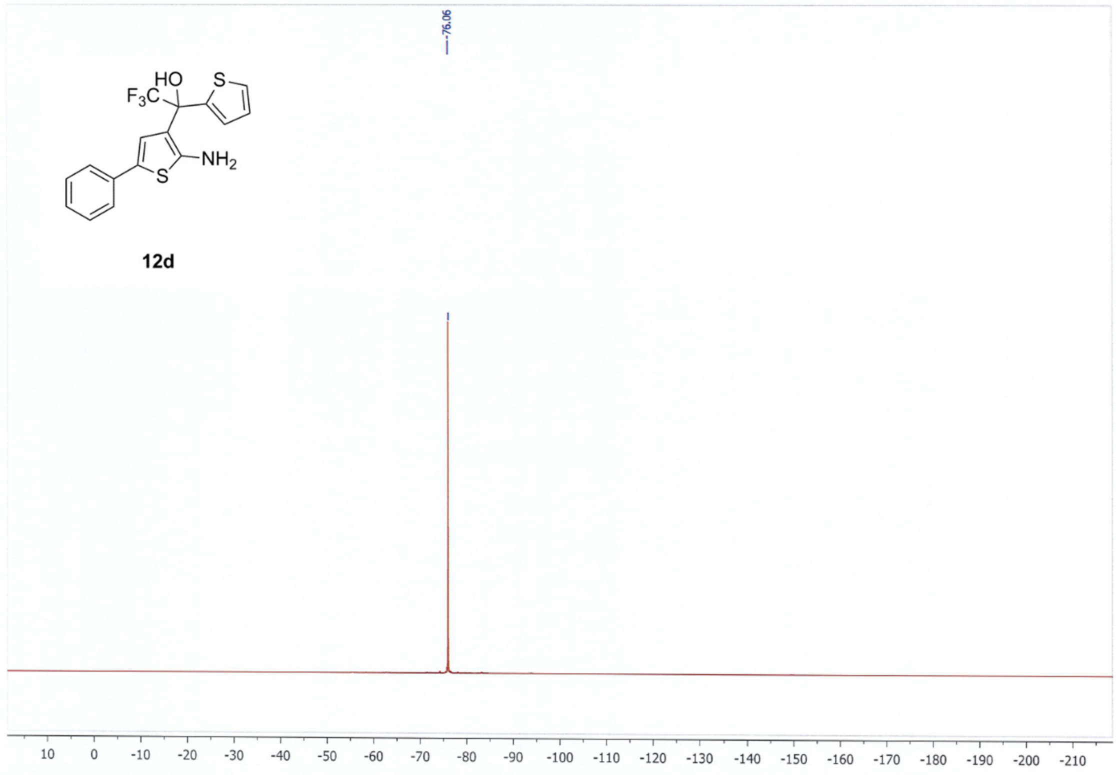
-76.06
HO
F3C
S
NH2
12d
10 0 -10 -20 -30 -40 -50 -60 -70 -80 -90 -100 -110 -120 -130 -140 -150 -160 -170 -180 -190 -200 -210

VD-MRH-65

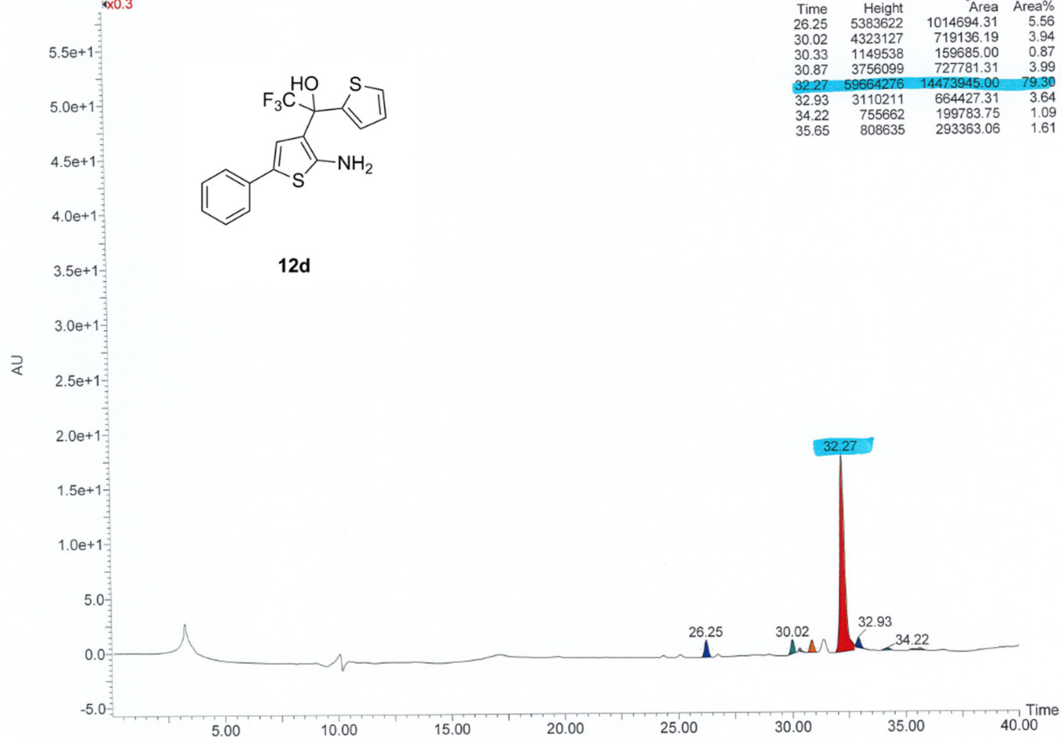

2: Diode Array
Range: 6.438e+1

Time	Height	Area	Area%
26.25	5383622	1014694.31	5.56
30.02	4323127	719136.19	3.94
30.33	1149538	159685.00	0.87
30.87	3756099	727781.31	3.99
32.27	59664276	14473945.00	79.30
32.93	3110211	664427.31	3.64
34.22	755662	199783.75	1.09
35.65	808635	293363.06	1.61

Elemental Composition Report

Page 1

Single Mass Analysis

Tolerance = 3.0 mDa / DBE: min = -1.0, max = 100.0
Element prediction: Off
Number of isotope peaks used for i-FIT = 3

Monoisotopic Mass, Even Electron Ions
702 formula(e) evaluated with 7 results within limits (up to 50 best isotopic matches for each mass)
Elements Used:
C: 0-100 H: 0-100 N: 0-20 O: 0-30 S: 1-2 F: 3-3

SYNAPT G2-S#NotSet VD-MRH-65 02-Feb-2021
CHR-2_7 12 (0.211) Cm (10:15) 1: TOF MS ES+
9.65e+004

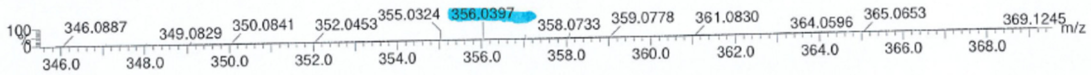

				-1.0				
Minimum:				-1.0				
Maximum:		3.0	3.0	100.0				
Mass	Calc. Mass	mDa	PPM	DBE	i-FIT	Norm	Conf(%)	Formula
356.0397	356.0391	0.6	1.7	9.5	2031.9	0.008	99.20	C16 H13 N O S2 F3
	356.0402	-0.5	-1.4	11.5	2037.2	5.243	0.53	C9 H5 N11 S F3
	356.0389	0.8	2.2	6.5	2038.7	6.775	0.11	C8 H9 N7 O4 S F3
	356.0416	-1.9	-5.3	5.5	2038.8	6.873	0.10	C12 H13 N O6 S F3
	356.0423	-2.6	-7.3	1.5	2039.9	7.989	0.03	C5 H13 N7 O4 S2 F3
	356.0375	2.2	6.2	1.5	2040.6	8.642	0.02	C7 H13 N3 O8 S F3
	356.0396	0.1	0.3	2.5	2044.4	12.481	0.00	C H9 N13 O2 S2 F3

*1-(2-amino-5-phenylthiophen-3-yl)-2,2,2-trifluoro-1-phenylethan-1-ol (**12e**)*

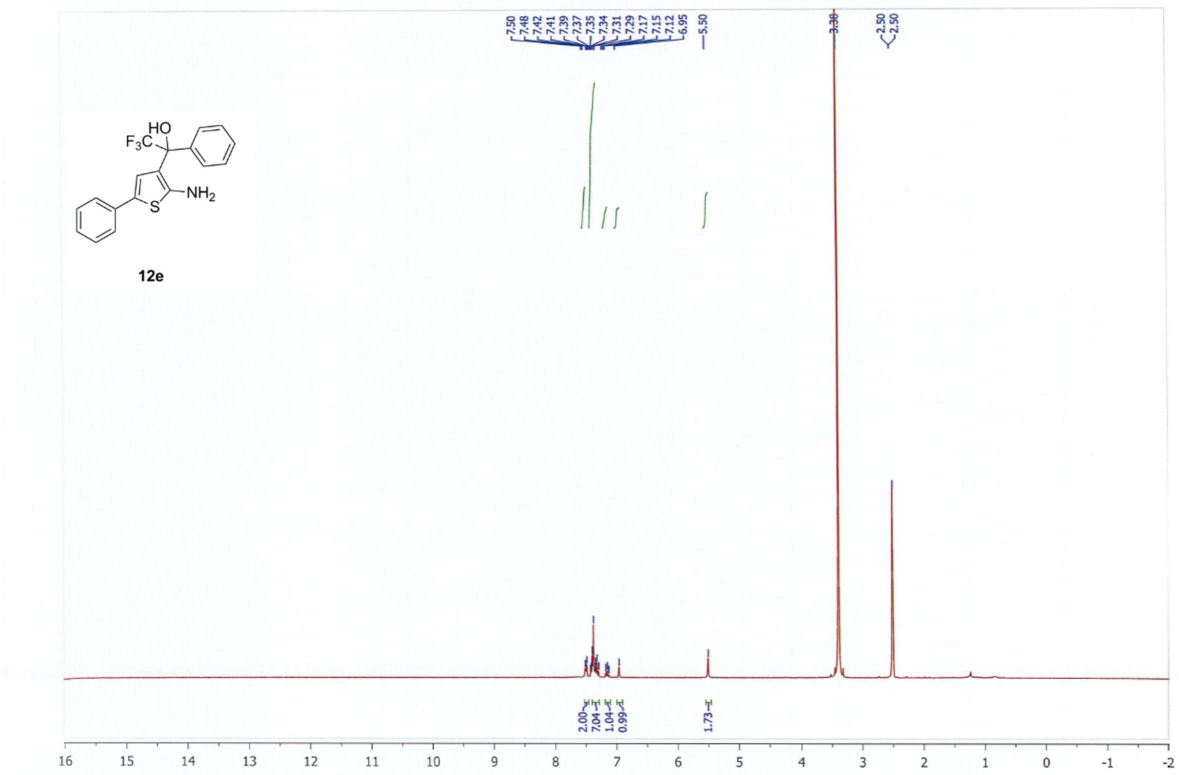

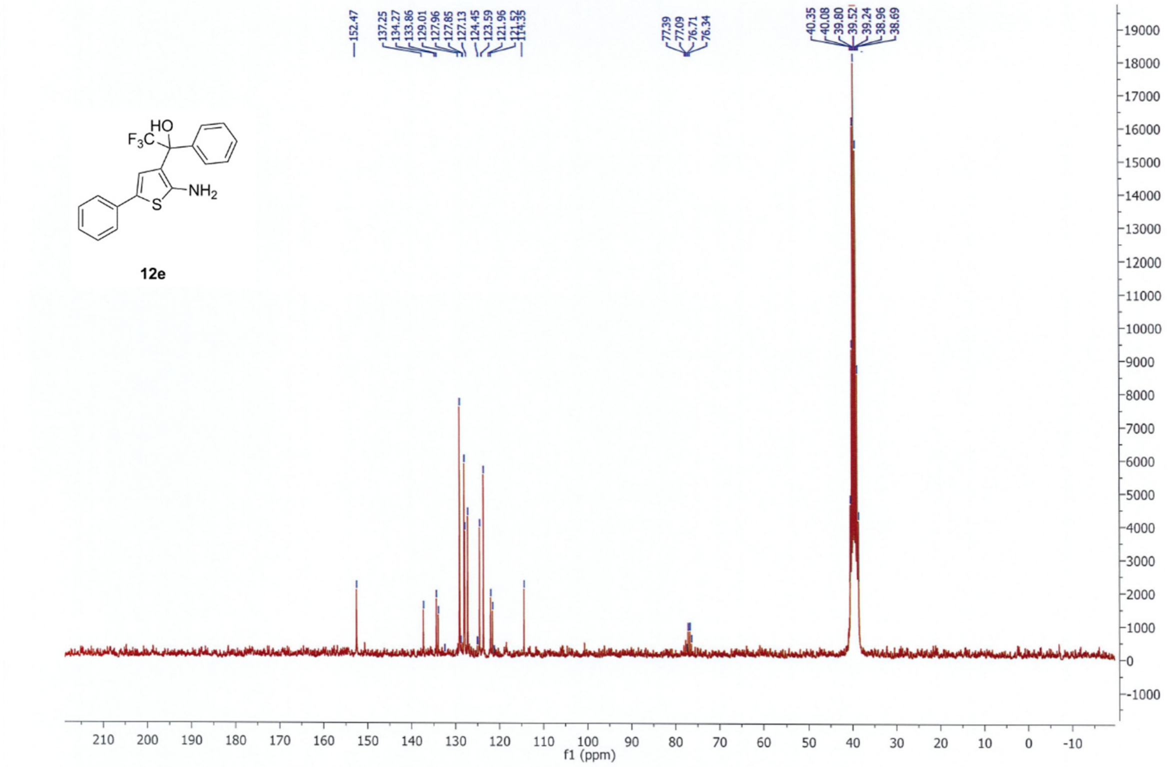

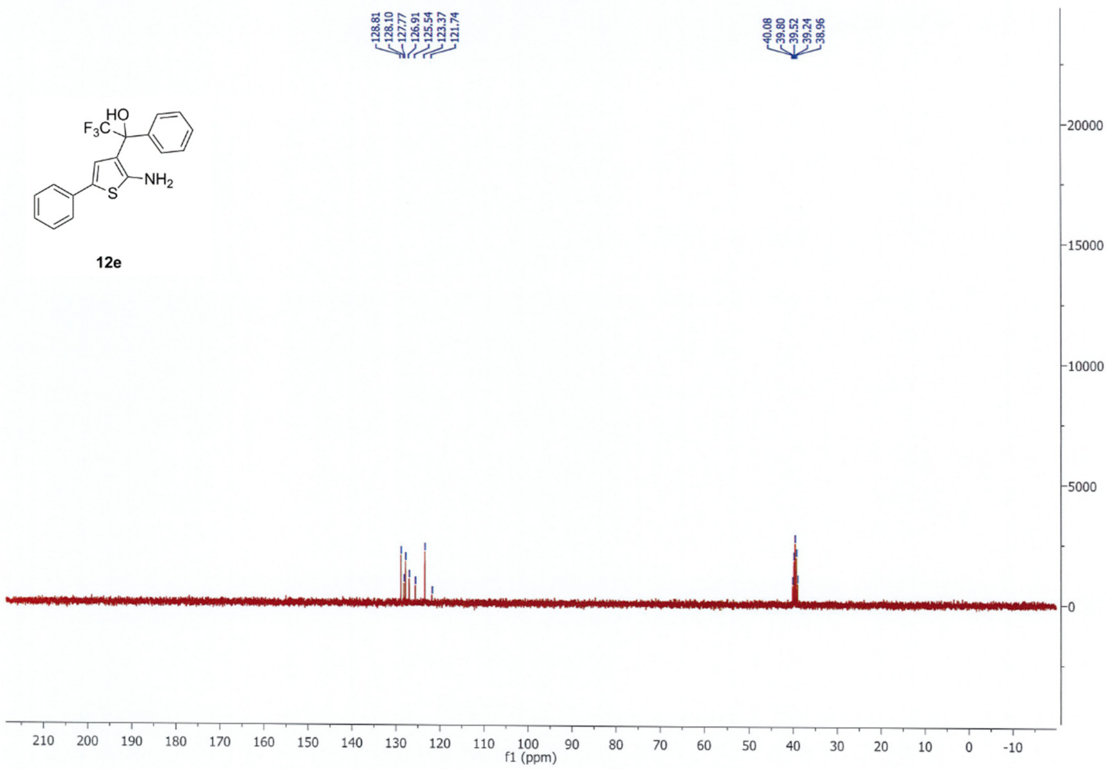
128.81
128.10
127.77
126.91
125.54
123.37
121.74
40.08
39.80
39.52
39.24
38.96
12e
f1 (ppm)

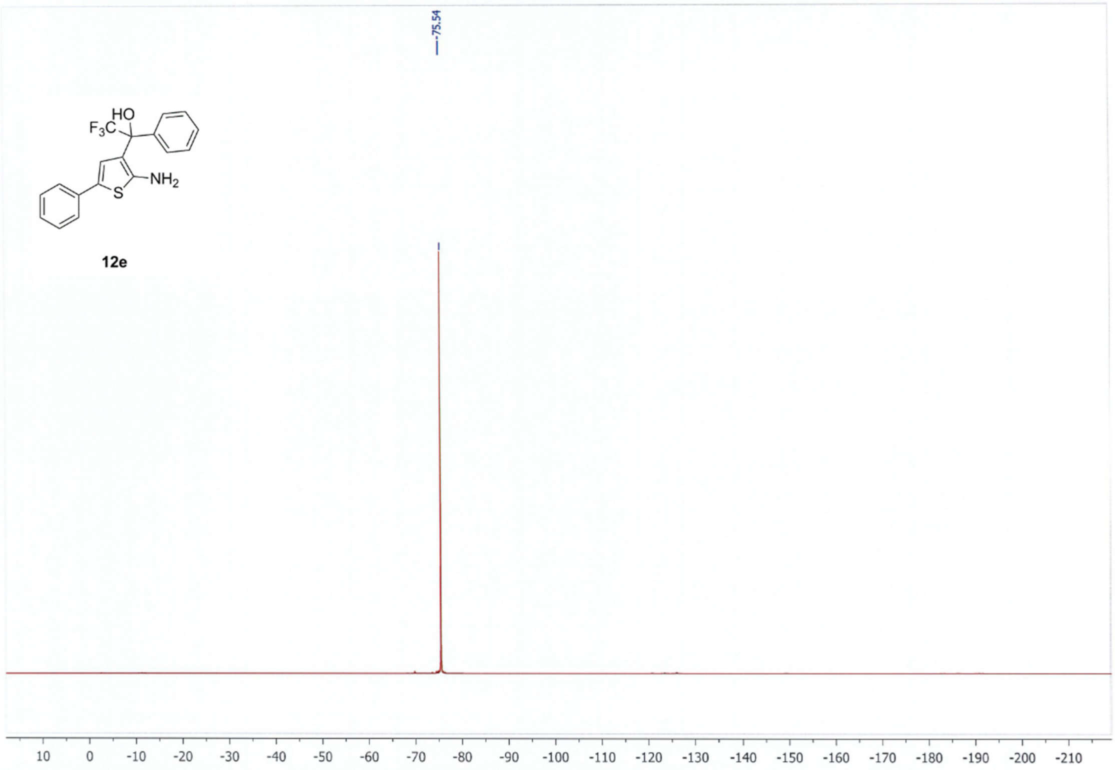
-75.54
12e

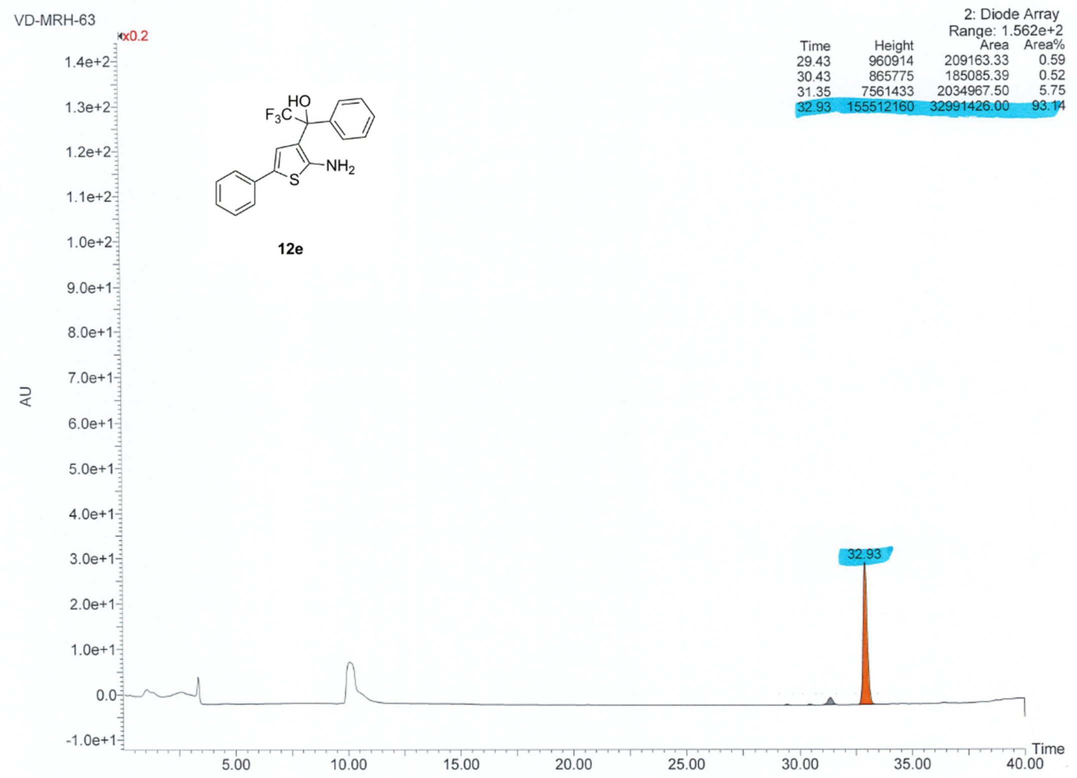

Elemental Composition Report

Page 1

Single Mass Analysis

Tolerance = 3.0 mDa / DBE: min = -1.0, max = 100.0
Element prediction: Off
Number of isotope peaks used for i-FIT = 3

Monoisotopic Mass, Even Electron Ions
384 formula(e) evaluated with 3 results within limits (up to 50 best isotopic matches for each mass)
Elements Used:
C: 0-100 H: 0-100 N: 0-20 O: 0-30 S: 1-1 F: 3-3

SYNAPT G2-S#NotSet
CHR-2_4 12 (0.211) Cm (9:13)

VD-MRH-63

02-Feb-2021
1: TOF MS ES+
1.93e+005

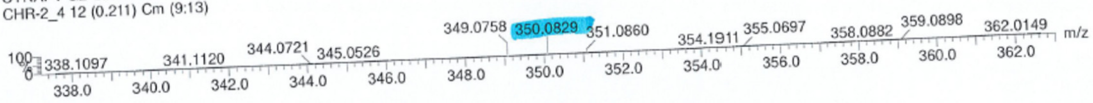

Minimum:				-1.0				
Maximum:		3.0	3.0	100.0				
Mass	Calc. Mass	mDa	PPM	DBE	i-FIT	Norm	Conf(%)	Formula
350.0829	350.0826	0.3	0.9	10.5	2205.9	0.020	98.03	C18 H15 N O S F3
	350.0858	-2.9	-8.3	2.5	2209.8	3.960	1.91	C7 H15 N7 O4 S F3
	350.0831	-0.2	-0.6	3.5	2213.2	7.326	0.07	C3 H11 N13 O2 S F3

*1-(2-amino-5-phenylthiophen-3-yl)-2,2,2-trifluoro-1-(p-tolyl)ethan-1-ol (**12f**)*

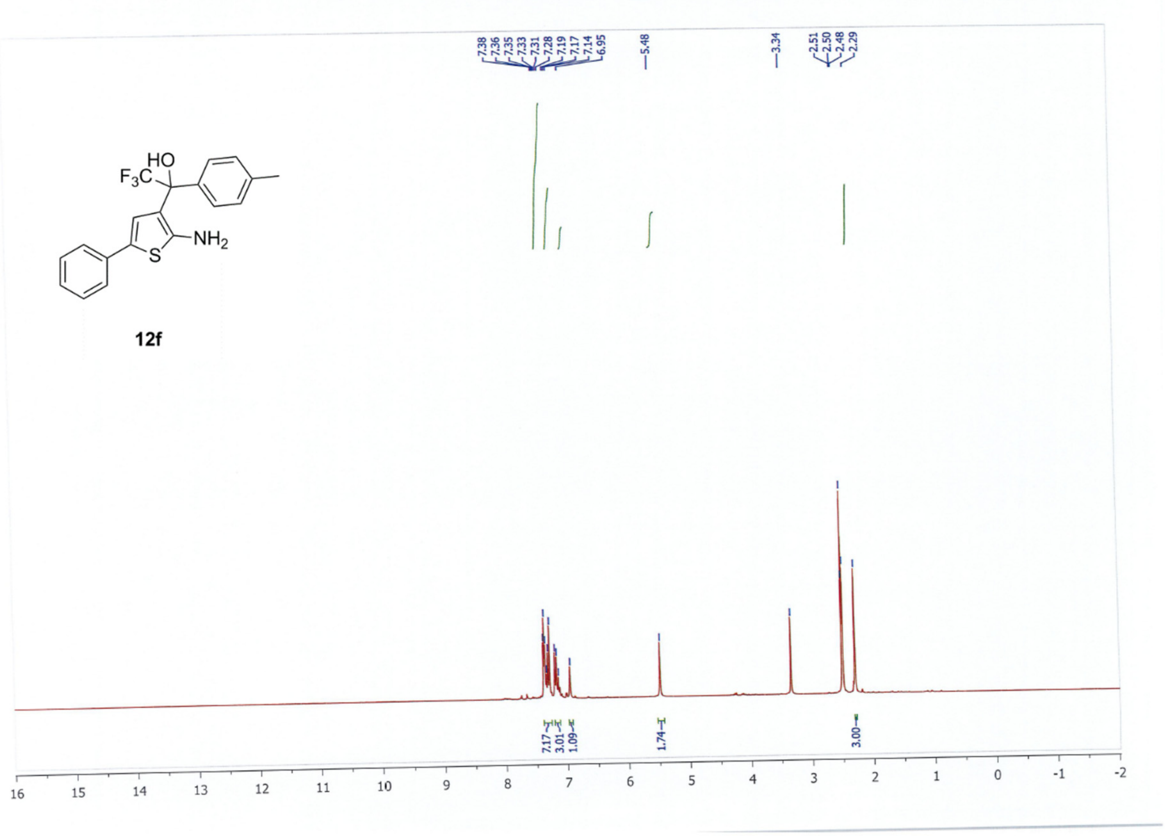

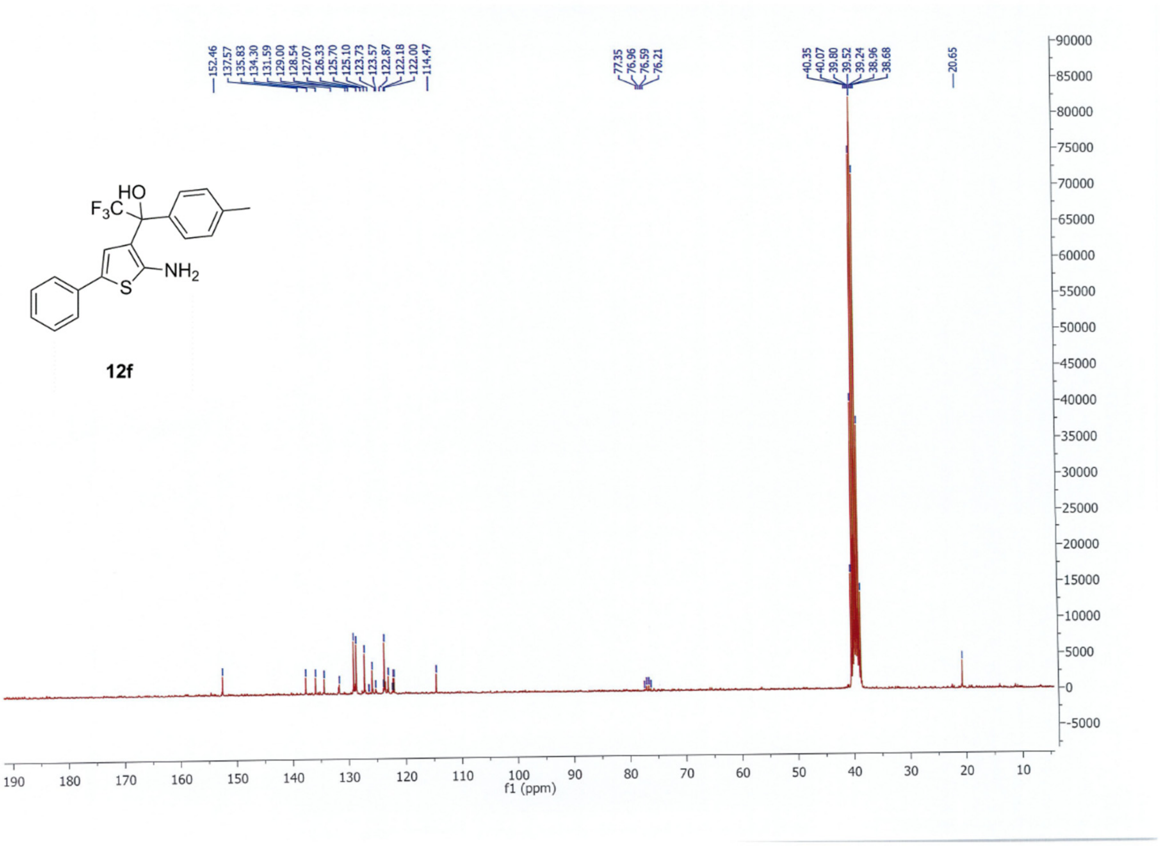

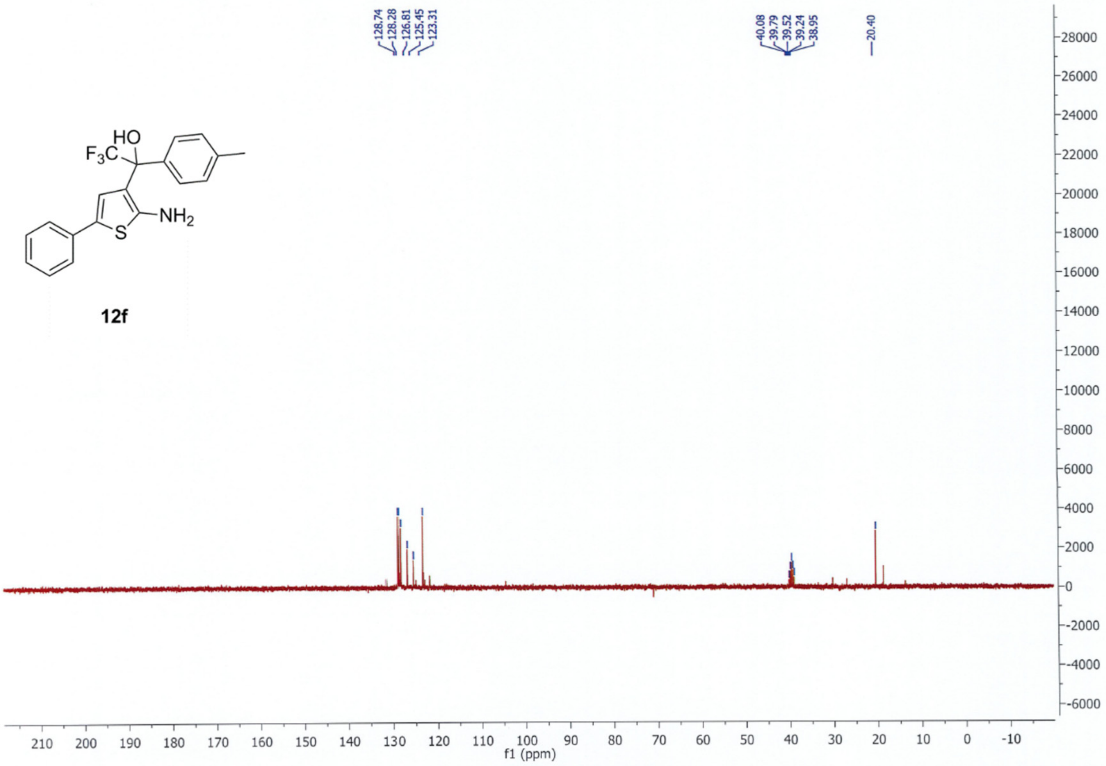

HO
F3C
NH2
S
128.74
128.28
126.81
125.45
123.31
40.08
39.79
39.52
39.24
38.95
20.40
28000
26000
24000
22000
20000
18000
16000
14000
12000
10000
8000
6000
4000
2000
0
-2000
-4000
-6000
210 200 190 180 170 160 150 140 130 120 110 100 90 80 70 60 50 40 30 20 10 0 -10
f1 (ppm)

12f

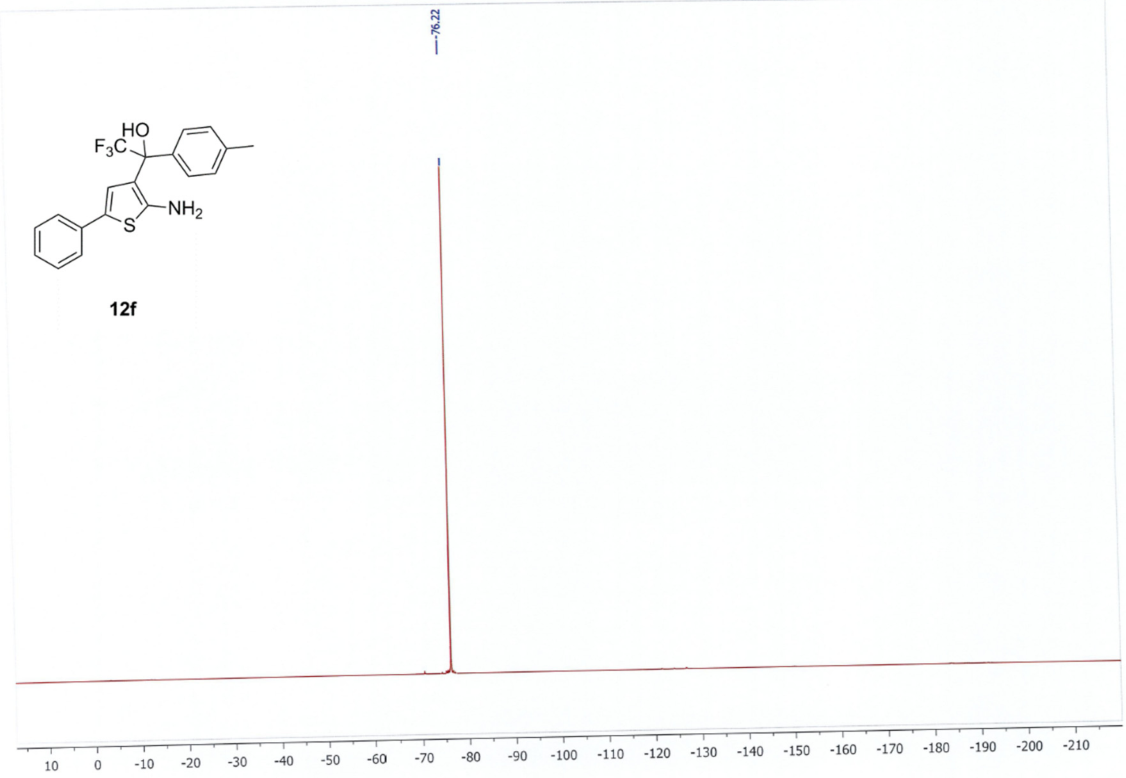

-76.22
HO
F3C
NH2
S
10 0 -10 -20 -30 -40 -50 -60 -70 -80 -90 -100 -110 -120 -130 -140 -150 -160 -170 -180 -190 -200 -210

12f

High Resolution Mass Result

**Analysis Info**		Acquisition Date	5/10/2021 2:52:18 PM
Sample Name	**VD-MRH-73**	Instrument / Ser#	micrOTOF-Q 228888.10300

Acquisition Parameter

Source Type	ESI	Ion Polarity	Positive	Scan Begin	50 m/z	Scan End	2200 m/z

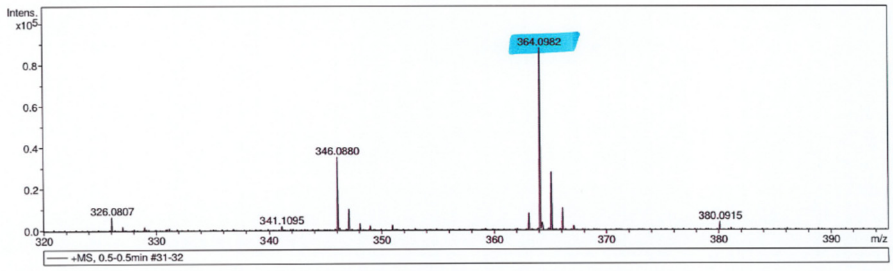

Meas. m/z	#	Ion Formula	m/z	err [ppm]	mSigma	# Sigma	Score	rdb	e⁻ Conf	N-Rule
364.0986	1	C19H17F3NOS	364.0977	-1.2	55.9	1	100.00	10.5	even	ok
	2	C8H17F3N7O4S	364.1009	-7.6	102.2	2	3.12	2.5	even	ok

Bruker Compass DataAnalysis 4.1 printed: 5/10/2021 3:40:11 PM Page 1 of 1

VD-MRH-70-F1

2: Diode Array
Range: 9.97e+1

Time	Height	Area	Area%
26.23	1866751	347896.84	1.06
27.27	2878627	1530056.75	4.66
31.88	849470	135632.48	0.41
32.63	440060	62000.40	0.19
34.05	95554400	26304918.00	80.06
34.47	9934475	2021991.00	6.15
36.12	3468855	975258.25	2.97
39.32	6981898	1478442.63	4.50

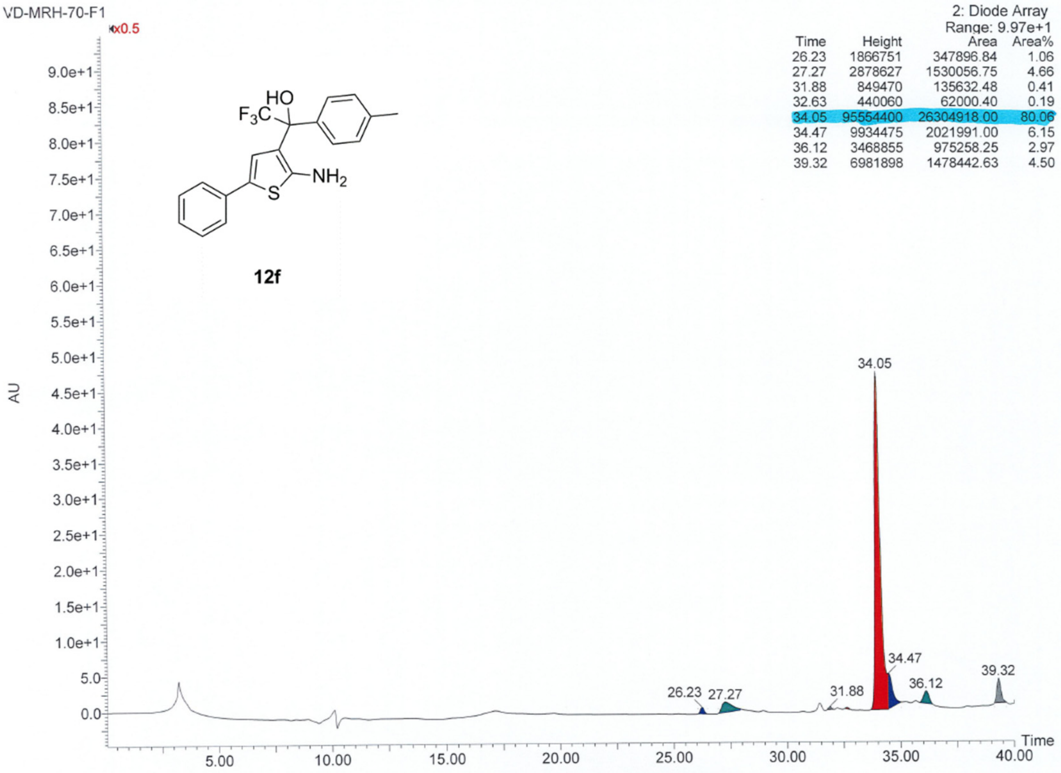

*1-(2-amino-5-phenylthiophen-3-yl)-2,2,2-trifluoro-1-(4-fluorophenyl)ethan-1-ol (**12g**)*

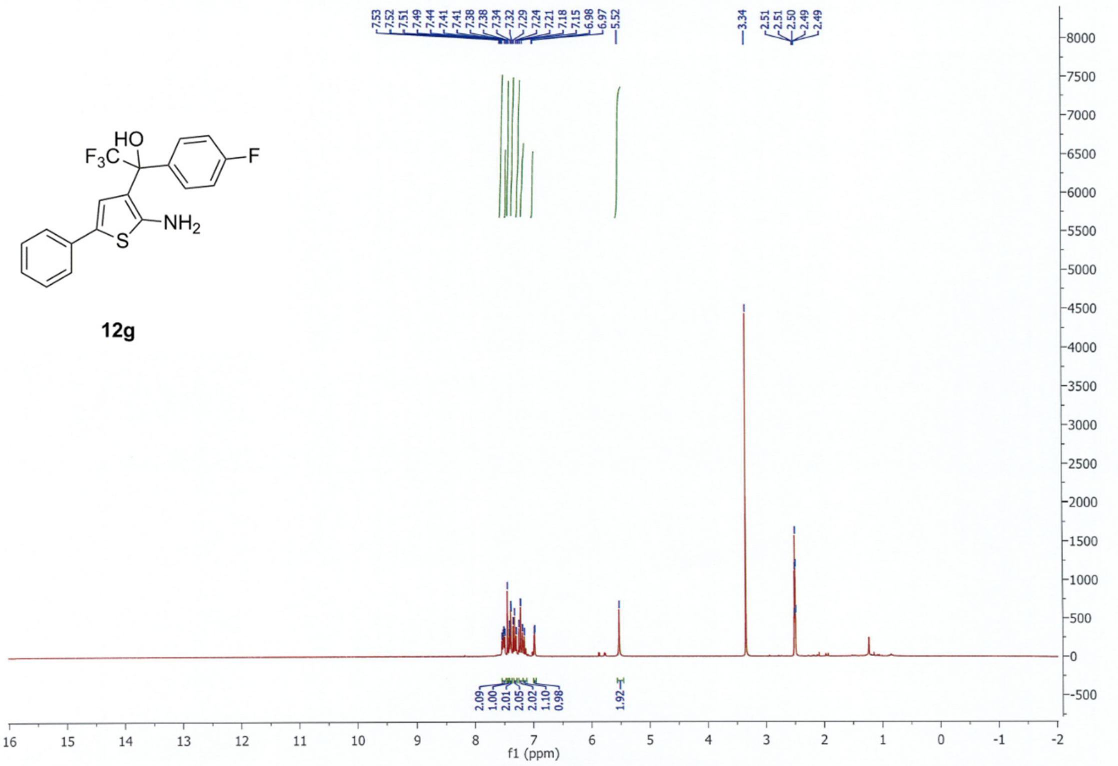

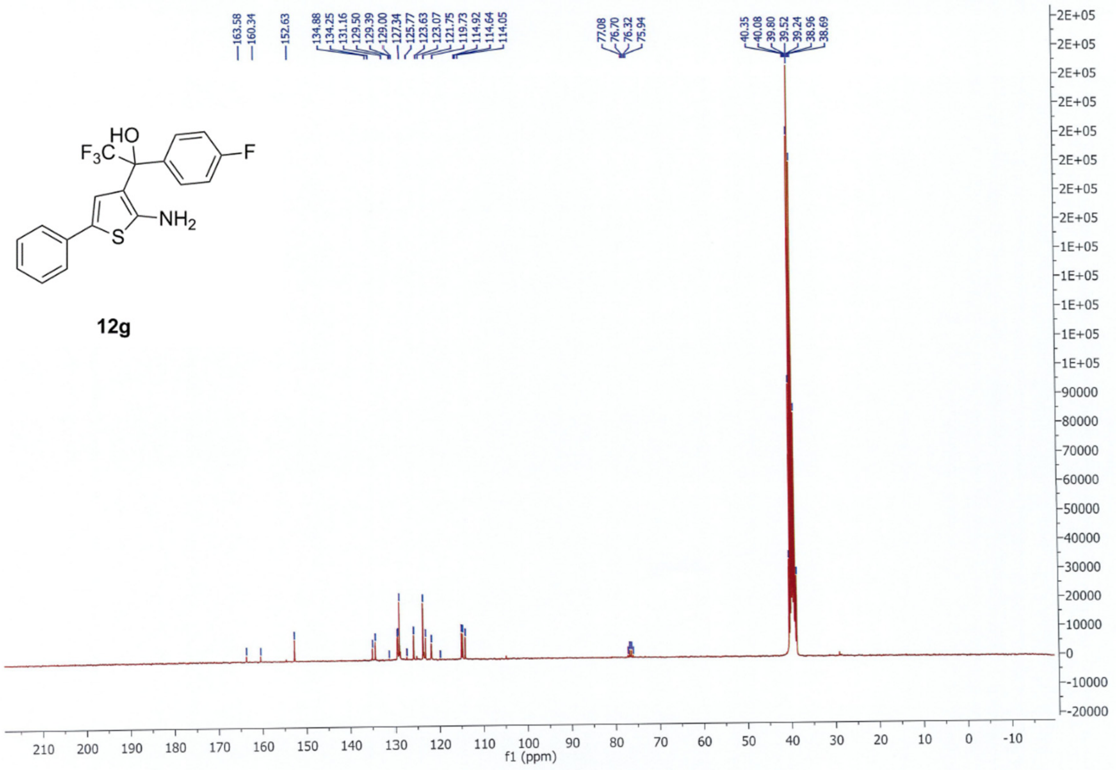

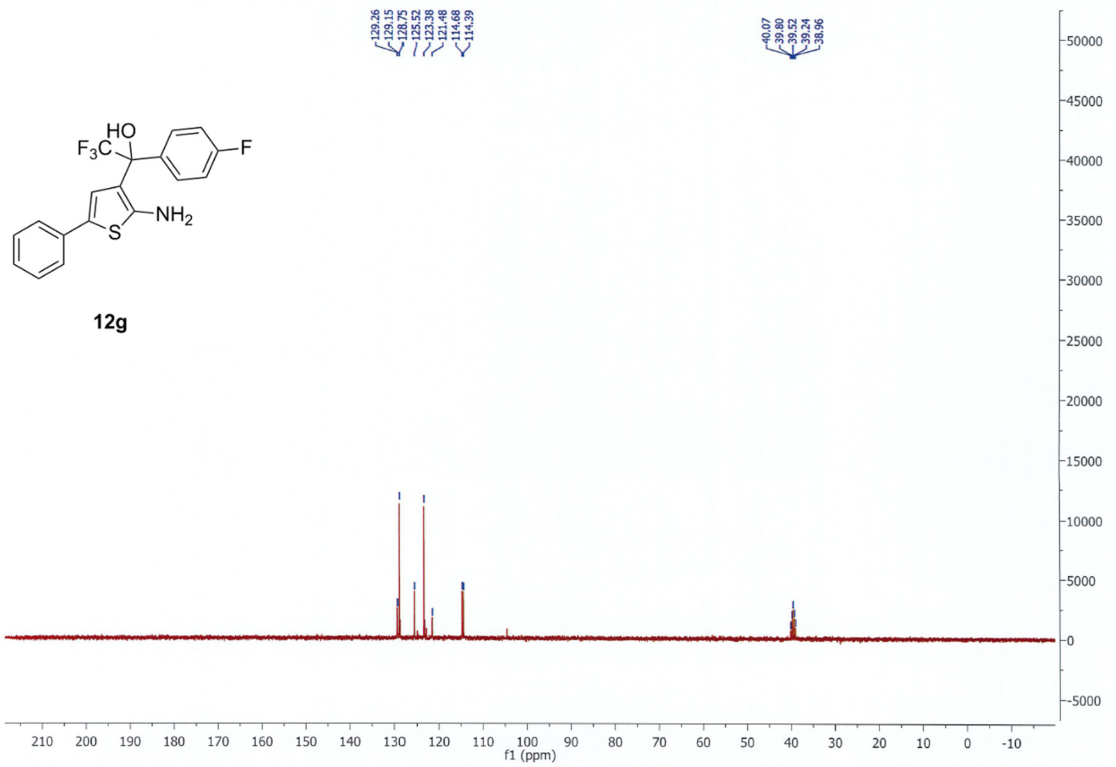

129.26
129.15
128.75
125.52
123.38
121.48
114.68
114.39
40.07
39.80
39.52
39.24
38.96
HO
F3C
F
NH2
S
12g
f1 (ppm)

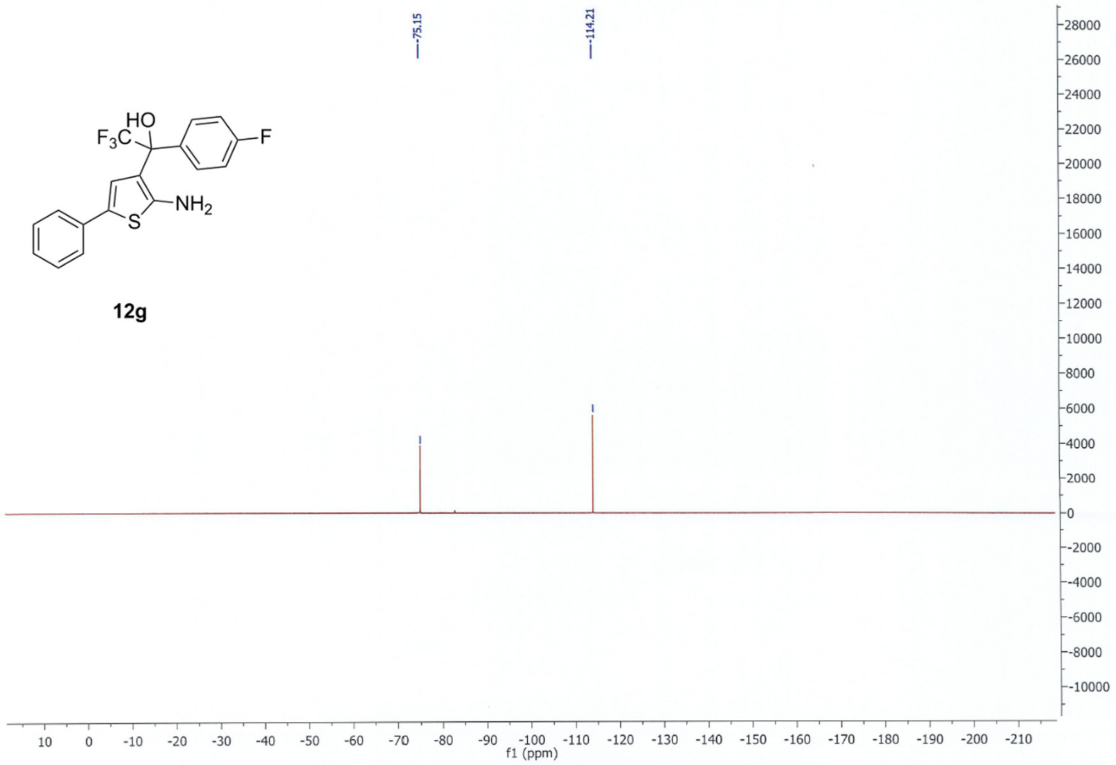

-75.15
-114.21
HO
F3C
F
NH2
S
12g
f1 (ppm)

High Resolution Mass Result

Analysis Info

Sample Name **DUV-44 _ VD-MRH-106**

Acquisition Date 7/23/2021 7:33:22 PM

Instrument / Ser# micrOTOF-Q 228888.10300

Acquisition Parameter

Source Type ESI | Ion Polarity Positive | Scan Begin 50 m/z | Scan End 2200 m/z

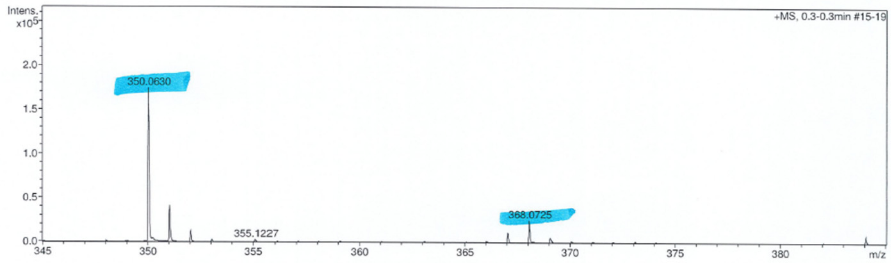

Meas. m/z	#	Ion Formula	m/z	err [ppm]	mSigma	# Sigma	Score	rdb	e⁻ Conf	N-Rule
350.0630	1	C18H12F4NS	350.0621	2.5	17.4	1	100.00	11.5	even	ok
	2	C6H16F4N3O7S	350.0640	2.8	76.3	2	16.27	-1.5	even	ok
	3	C3H8F4N13OS	350.0626	-1.0	76.8	3	22.06	4.5	even	ok
368.0725	1	C18H14F4NOS	368.0727	0.5	21.2	1	100.00	10.5	even	ok
	2	C3H10F4N13O2S	368.0732	-1.8	76.1	2	15.06	3.5	even	ok
	3	C2H14F4N9O6S	368.0718	-1.8	98.2	3	4.41	-1.5	even	ok

Bruker Compass DataAnalysis 4.1 printed: 7/26/2021 10:22:23 AM Page 1 of 1

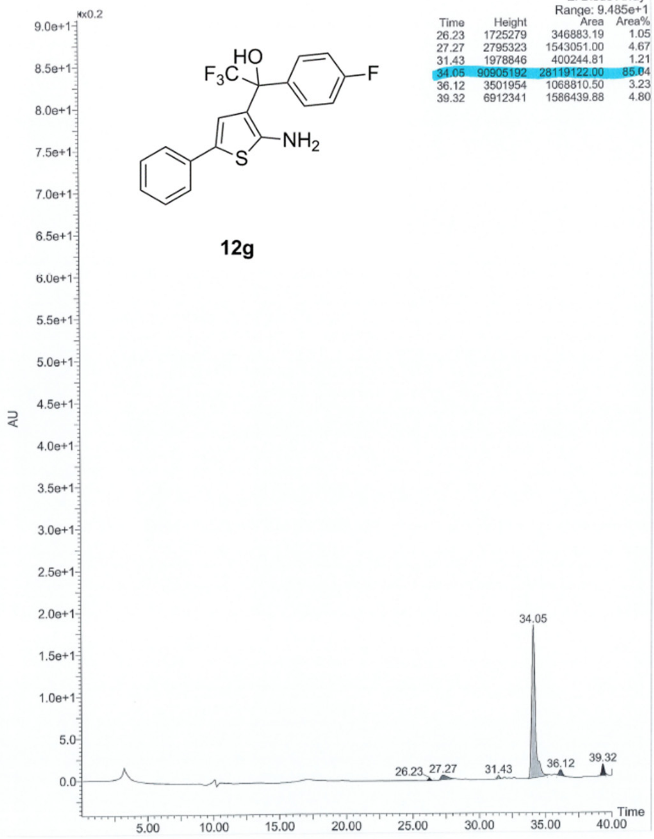

12g

2: Diode Array
Range: 9.485e+1

Time	Height	Area	Area%
26.23	1725279	346883.19	1.05
27.27	2795323	1543051.00	4.67
31.43	1978846	400244.81	1.21
34.05	90905192	28119122.00	85.04
36.12	3501954	1068810.50	3.23
39.32	6912341	1586439.88	4.80

*1-(2-amino-5-phenylthiophen-3-yl)-1-(4-chlorophenyl)-2,2,2-trifluoroethan-1-ol (**12h**)*

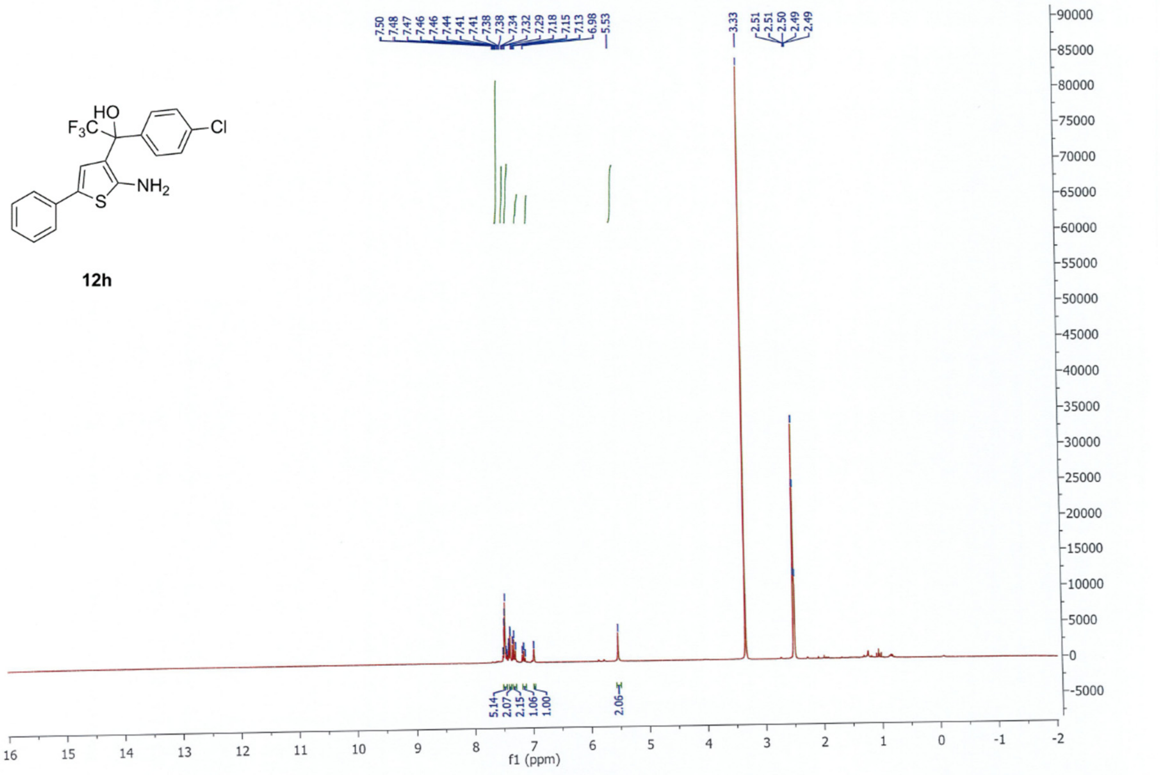

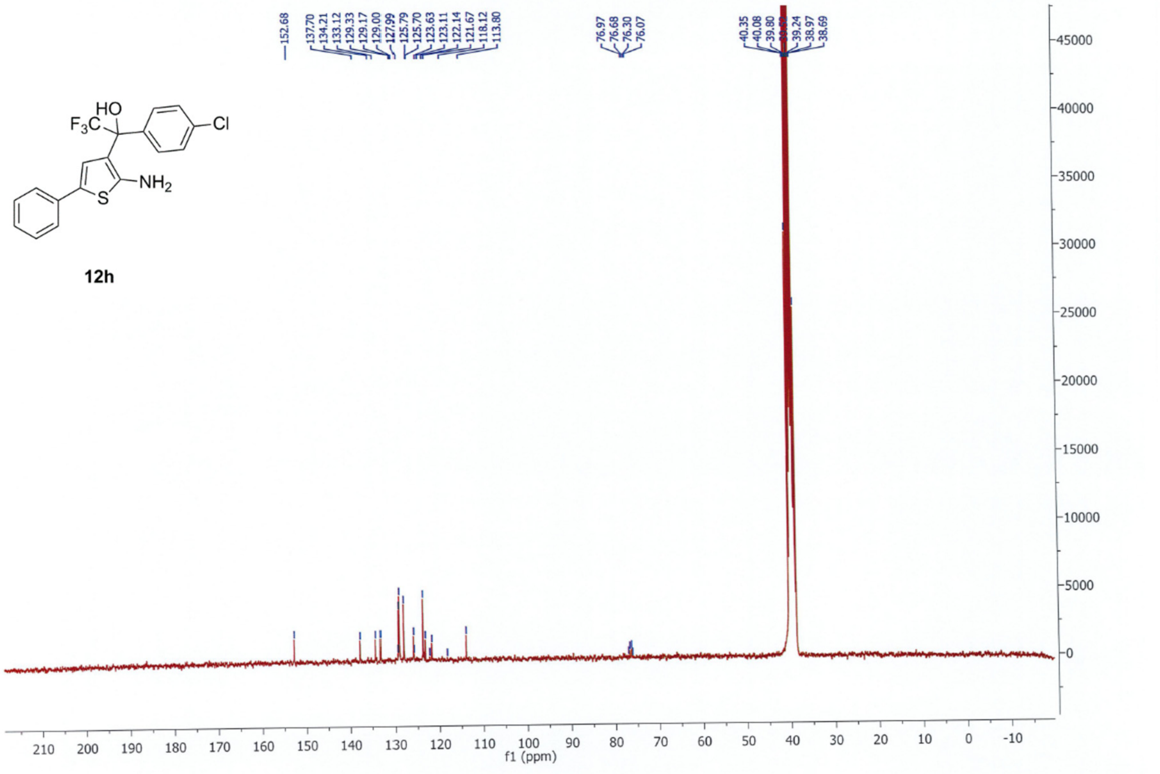

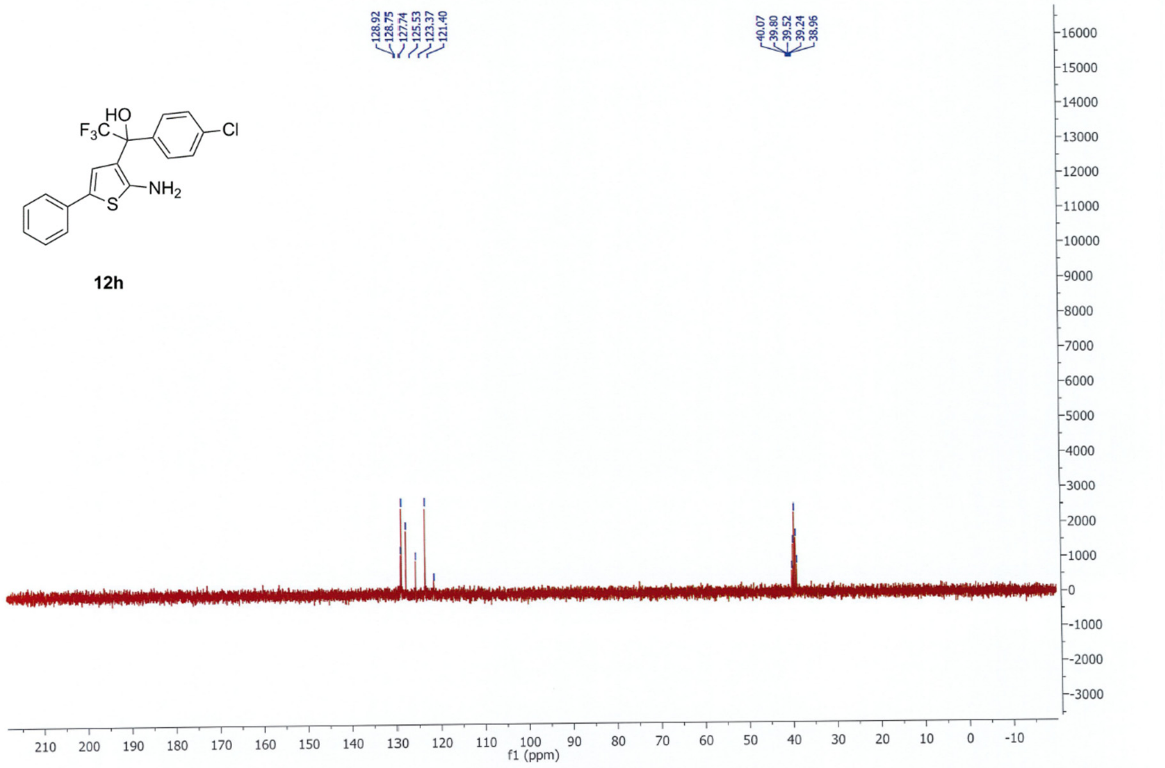
128.92
128.75
127.74
125.53
123.37
121.40
40.07
39.80
39.52
39.24
38.96
HO
F3C
Cl
NH2
S
12h
f1 (ppm)

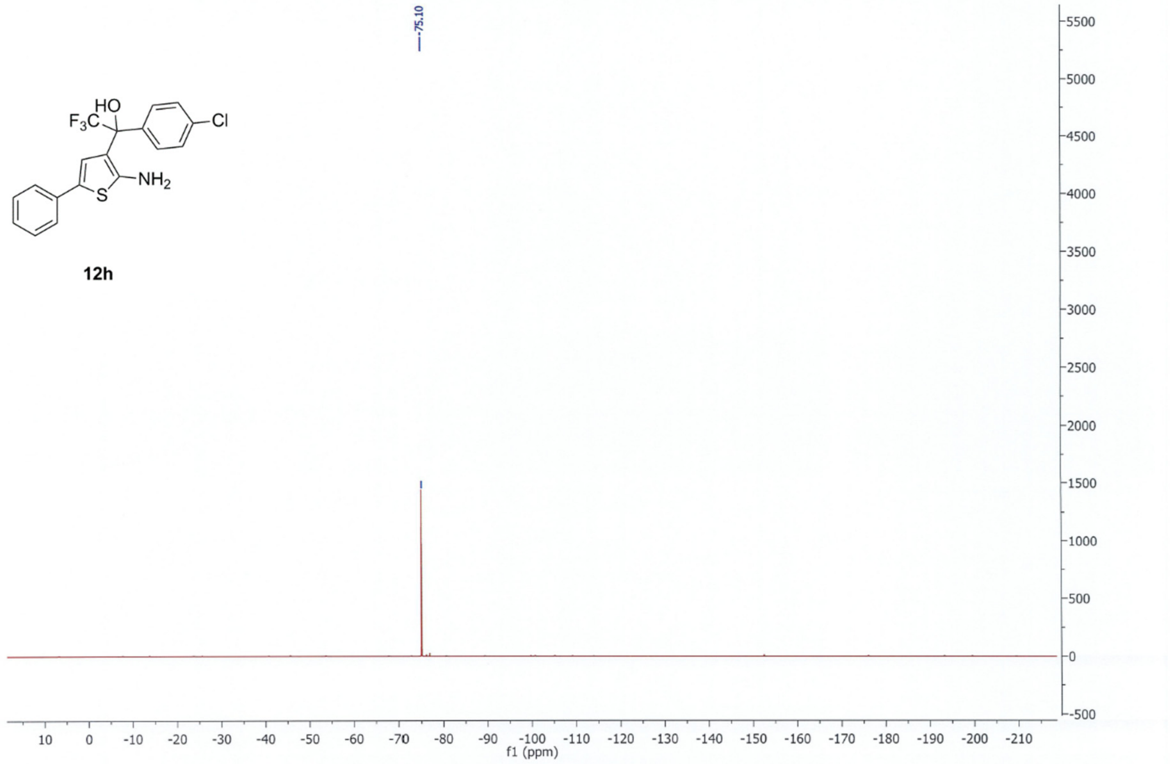
-75.10
HO
F3C
Cl
NH2
S
12h
f1 (ppm)

High Resolution Mass Result

**Analysis Info**		Acquisition Date	7/23/2021 6:53:29 PM
Sample Name	**DUV-46 _ VD-MRH-108**	Instrument / Ser#	micrOTOF-Q 228888.10300

Acquisition Parameter

Source Type	ESI	Ion Polarity	Positive	Scan Begin	50 m/z	Scan End	2200 m/z

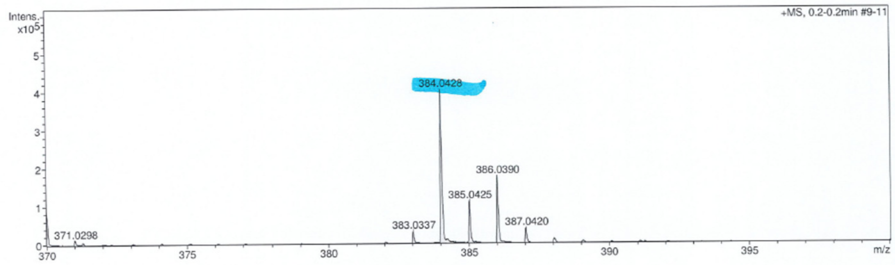

Meas. m/z	#	Ion Formula	m/z	err [ppm]	mSigma	# Sigma	Score	rdb	e⁻ Conf	N-Rule
384.0428	1	C18H14ClF3NOS	384.0431	-0.8	35.8	1	100.00	10.5	even	ok
	2	C3H10ClF3N13O2S	384.0436	-2.1	93.2	2	8.56	3.5	even	ok
	3	C2H14ClF3N9O6S	384.0423	-1.4	103.0	3	6.30	-1.5	even	ok

Bruker Compass DataAnalysis 4.1 printed: 7/23/2021 7:07:19 PM Page 1 of 1

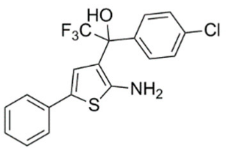

12h

2: Diode Array
Range: 4.253e+2

Time	Height	Area	Area%
32.13	412630976	122272192.00	84.98
34.75	13541008	3854901.50	2.68
35.28	6813536	1755174.88	1.22
38.75	12268167	2989635.50	2.08
39.83	65220656	13009026.00	9.04

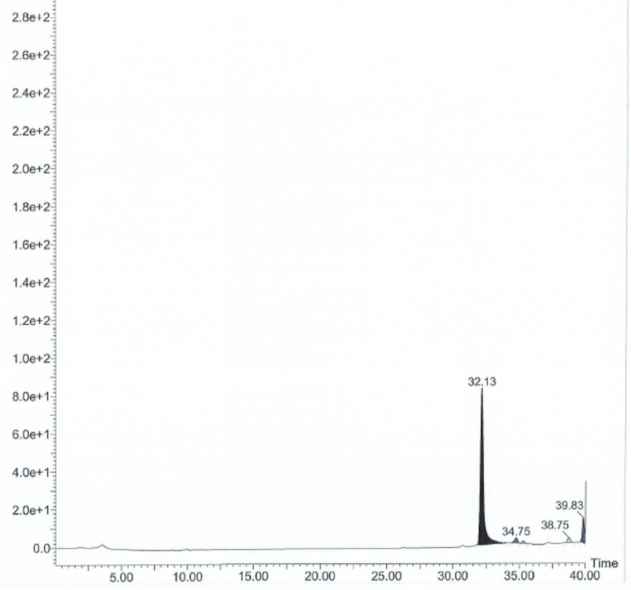

*1-(2-amino-5-phenylthiophen-3-yl)-1-(4-bromophenyl)-2,2,2-trifluoroethan-1-ol (**12i**)*

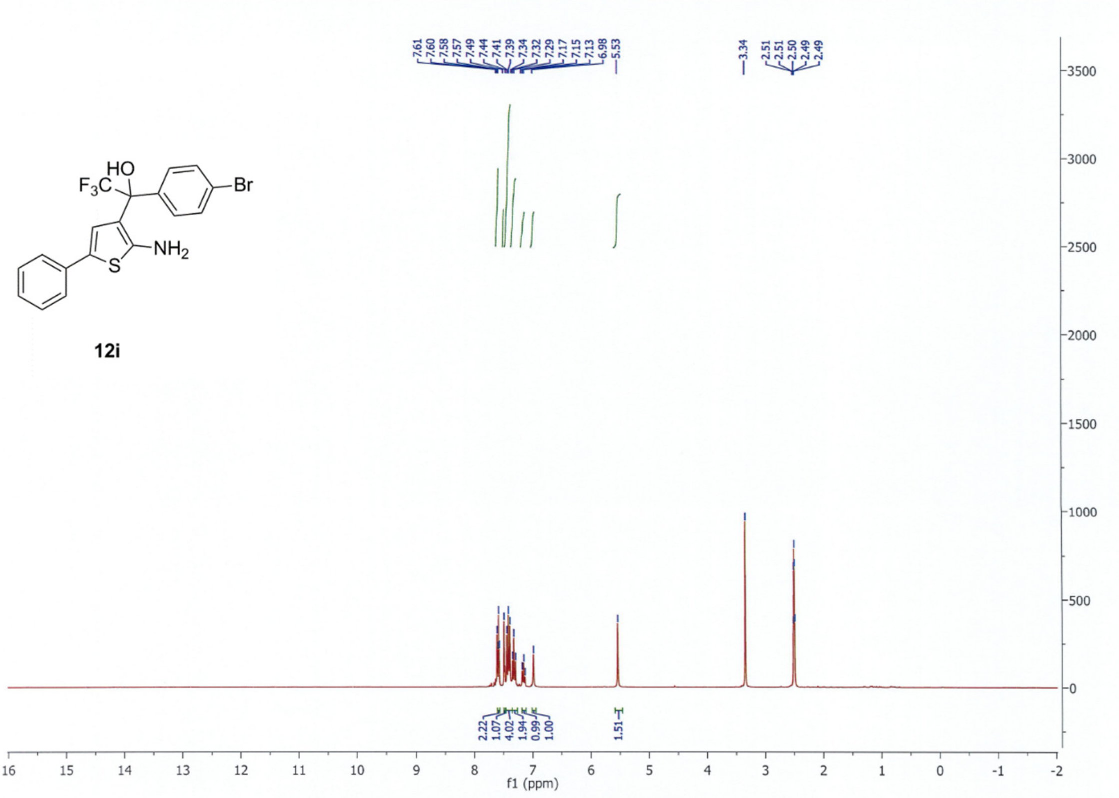

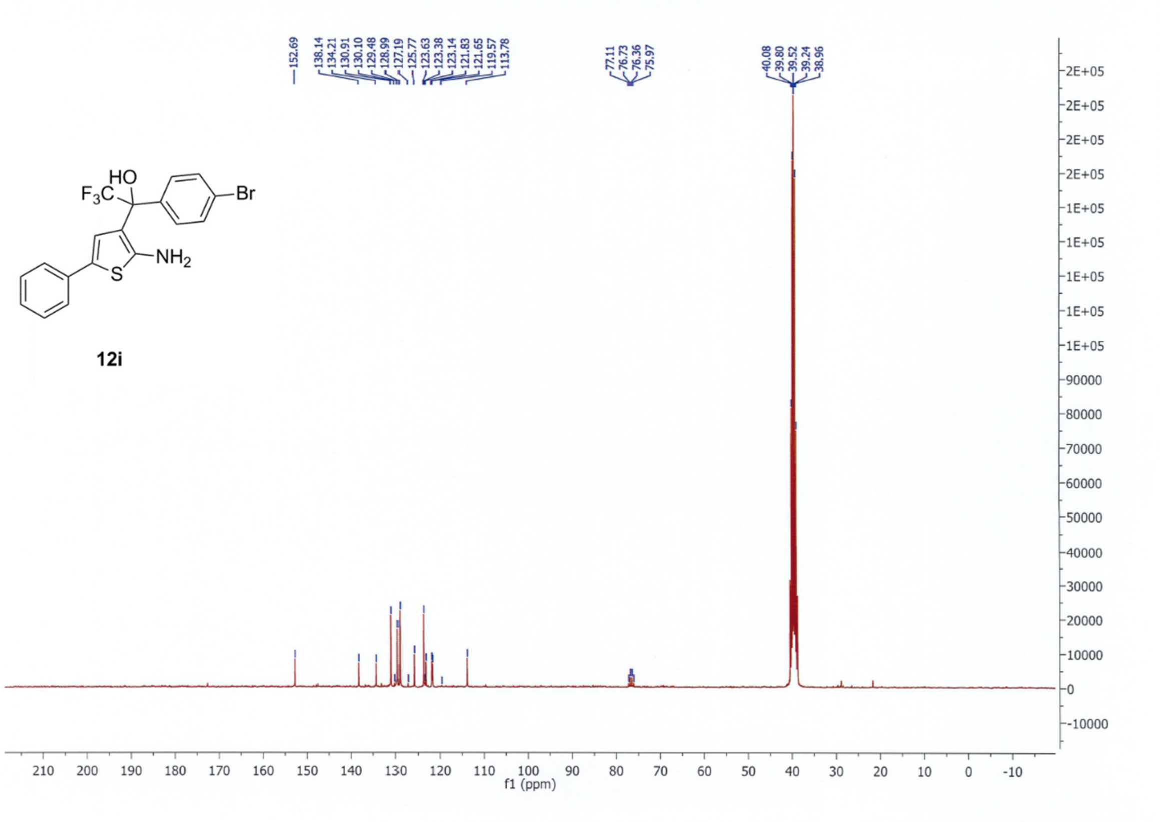

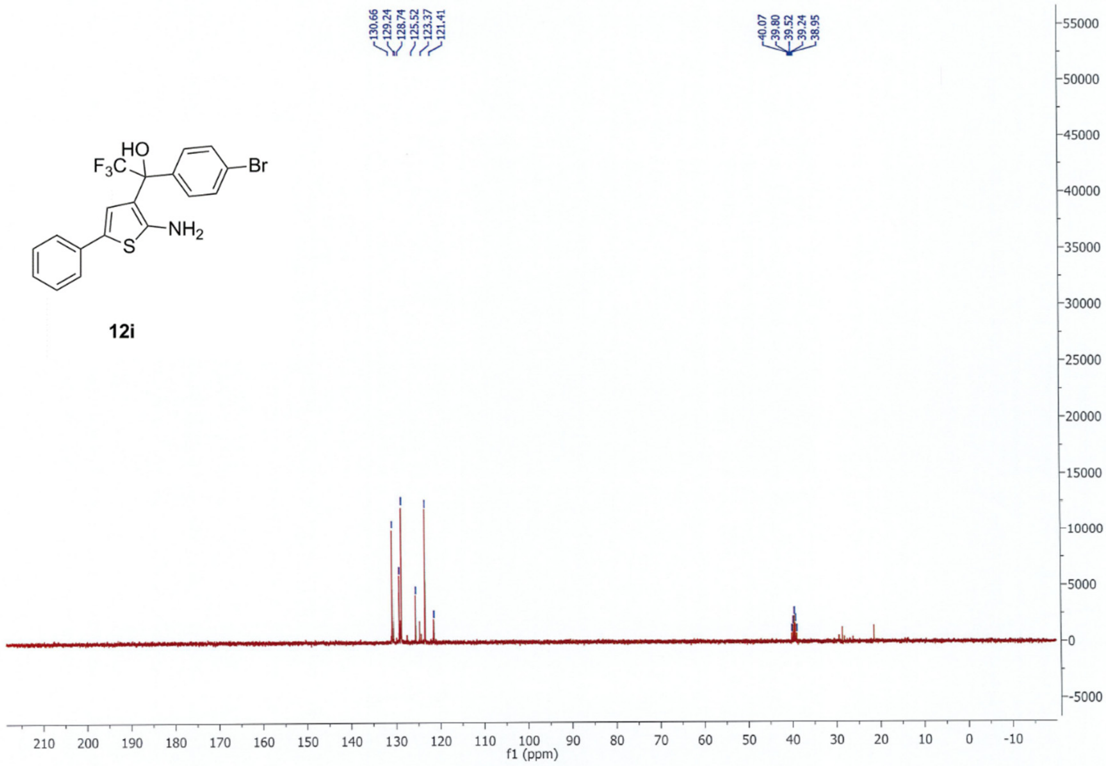
130.66
129.24
128.74
125.52
123.37
121.41
40.07
39.80
39.52
39.24
38.95
F3C
HO
Br
NH2
S
f1 (ppm)

12i

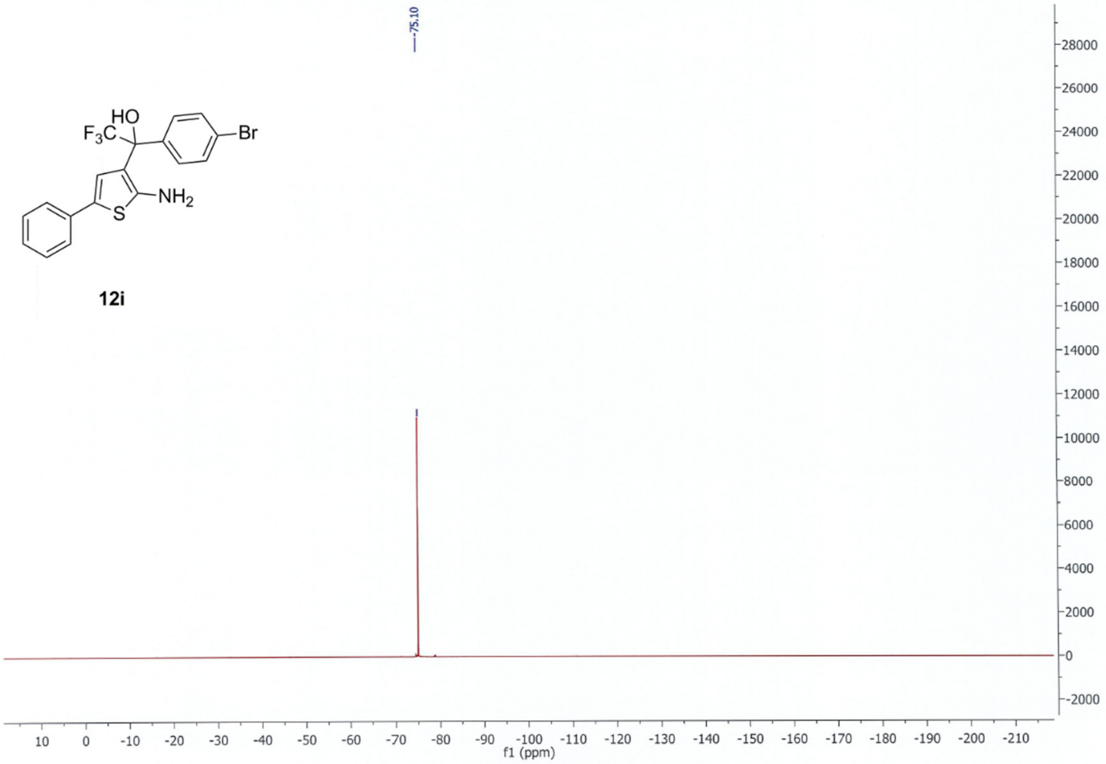
-75.10
F3C
HO
Br
NH2
S
f1 (ppm)

12i

High Resolution Mass Result

Analysis Info

Sample Name	**DUV-45 _ VD-MRH-107**	Acquisition Date	7/23/2021 6:50:28 PM
		Instrument / Ser#	micrOTOF-Q 228888.10300

Acquisition Parameter

Source Type	ESI	Ion Polarity	Positive	Scan Begin	50 m/z	Scan End	2200 m/z

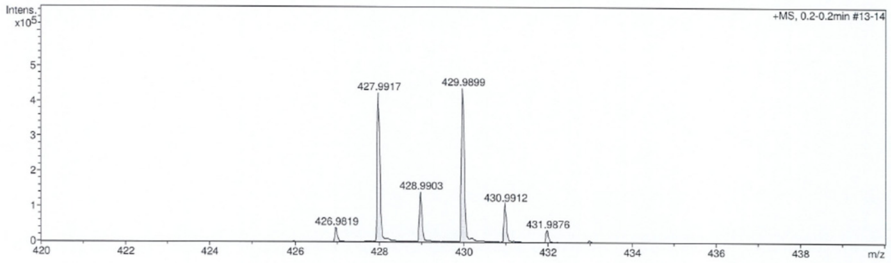

Meas. m/z	#	Ion Formula	m/z	err [ppm]	mSigma	# Sigma	Score	rdb	e⁻ Conf	N-Rule
427.9917	1	C18H14BrF3NOS	427.9926	-2.1	54.9	1	100.00	10.5	even	ok
	2	C2H14BrF3N9O6S	427.9918	0.2	143.3	2	1.91	-1.5	even	ok
	3	CH18BrF3N5O10S	427.9904	3.0	157.6	3	0.40	-6.5	even	ok

Bruker Compass DataAnalysis 4.1 printed: 7/23/2021 7:01:48 PM Page 1 of 1

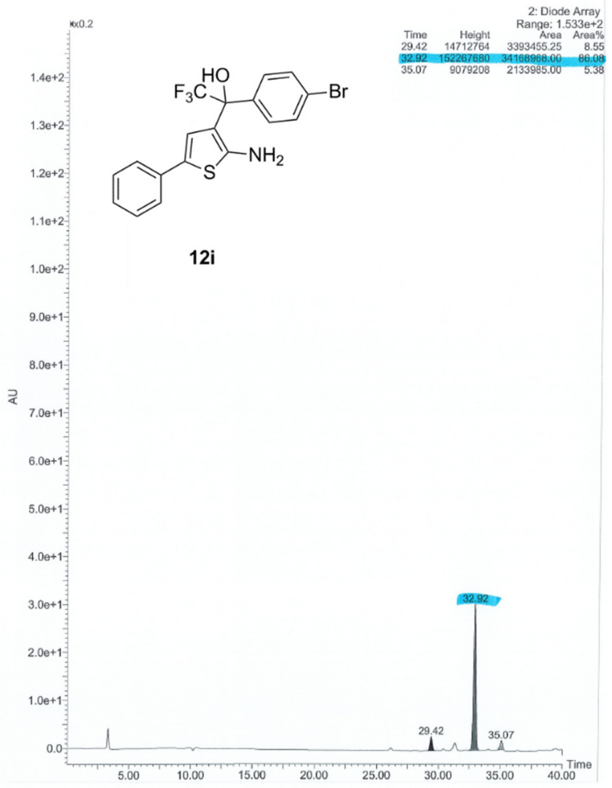

*1-(2-amino-5-phenylthiophen-3-yl)-2,2,2-trifluoro-1-(pyridin-2-yl)ethan-1-ol (**12j**)*

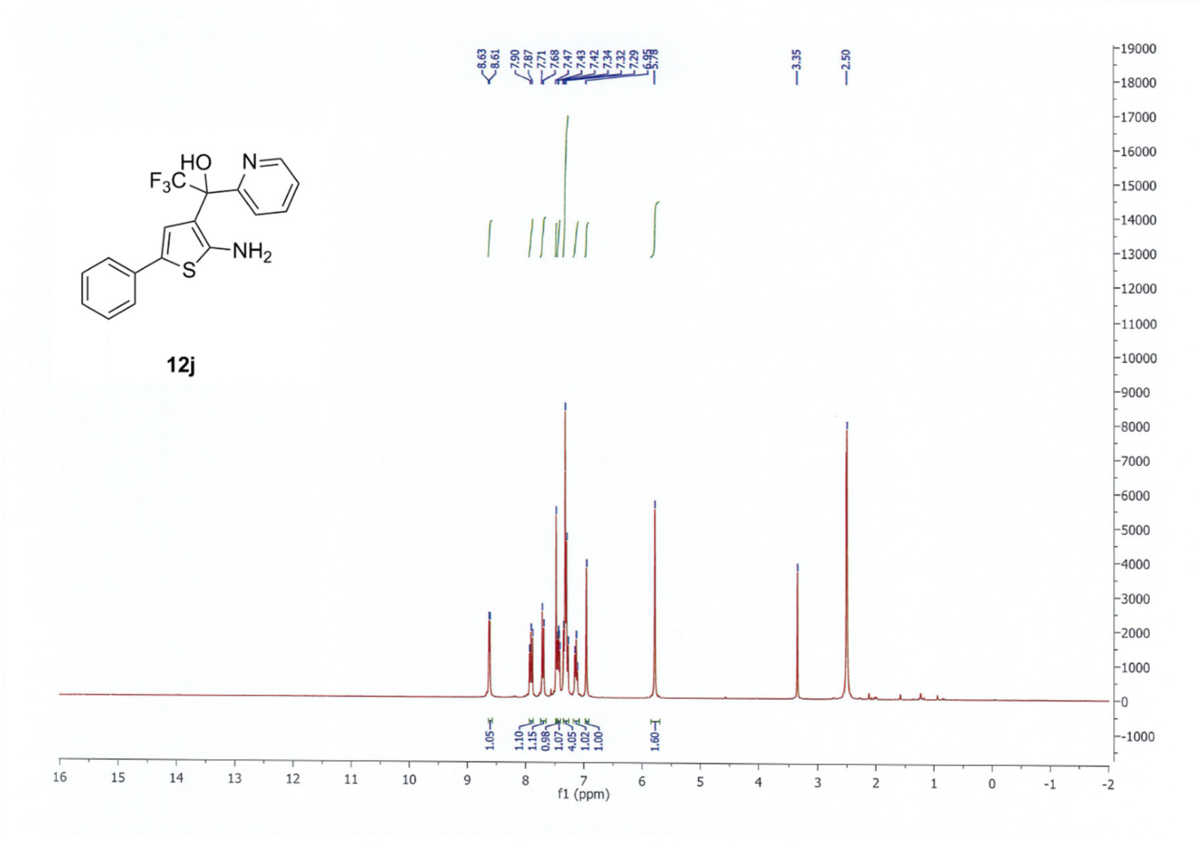

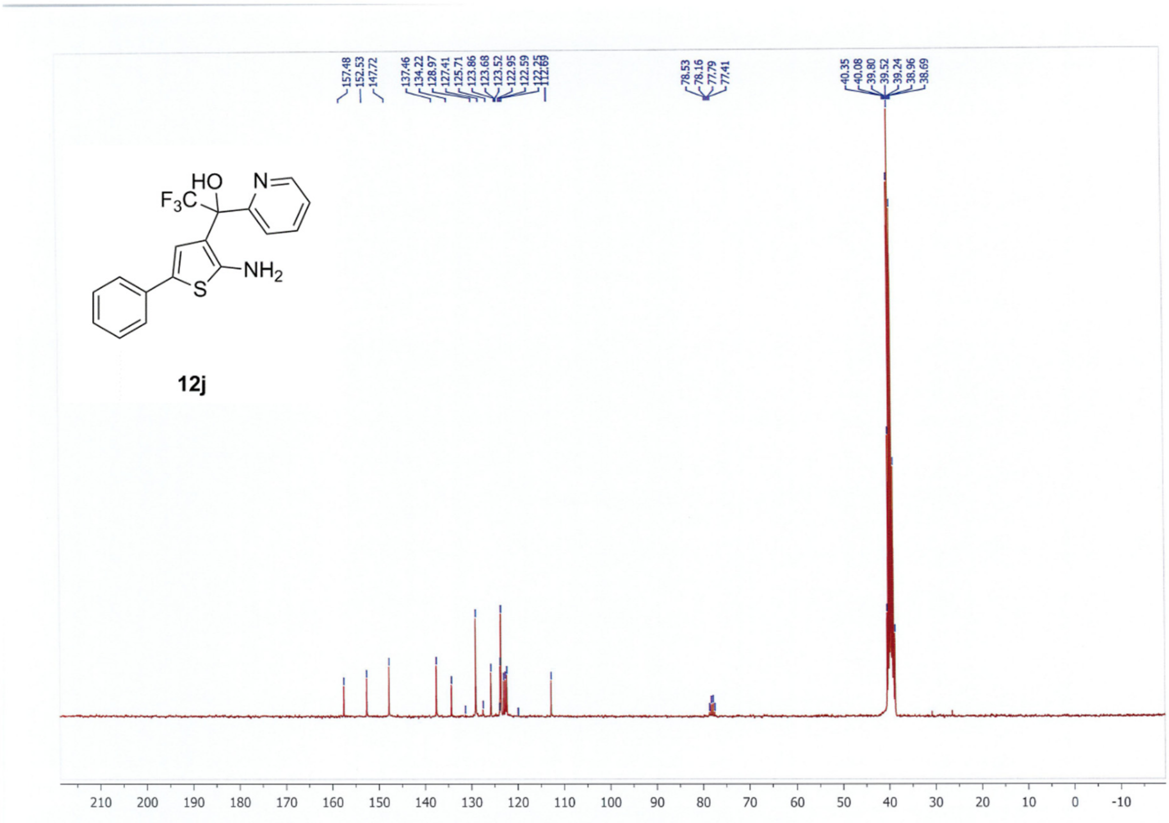

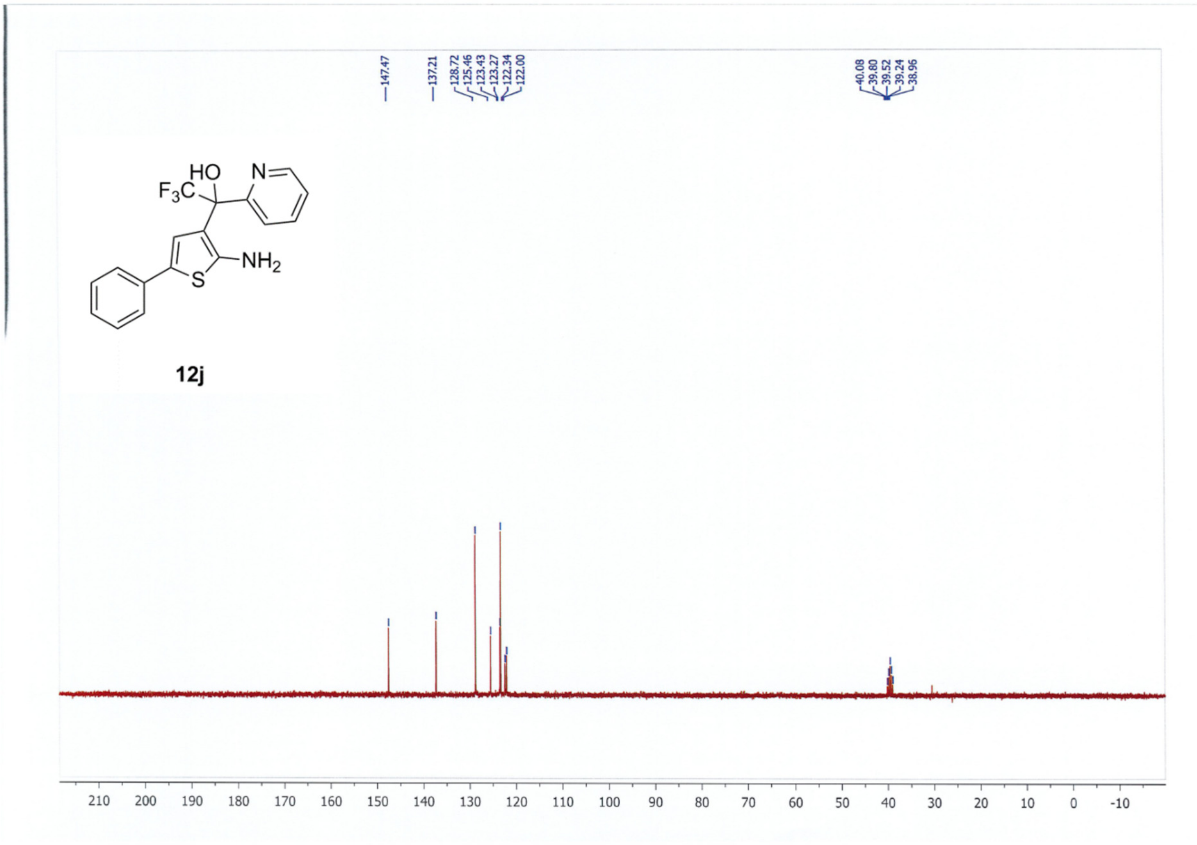
147.47
137.21
128.72
125.46
123.43
123.27
122.34
122.00
40.08
39.80
39.52
39.24
38.96
HO
N
F_3C
NH_2
S
12j
210 200 190 180 170 160 150 140 130 120 110 100 90 80 70 60 50 40 30 20 10 0 -10

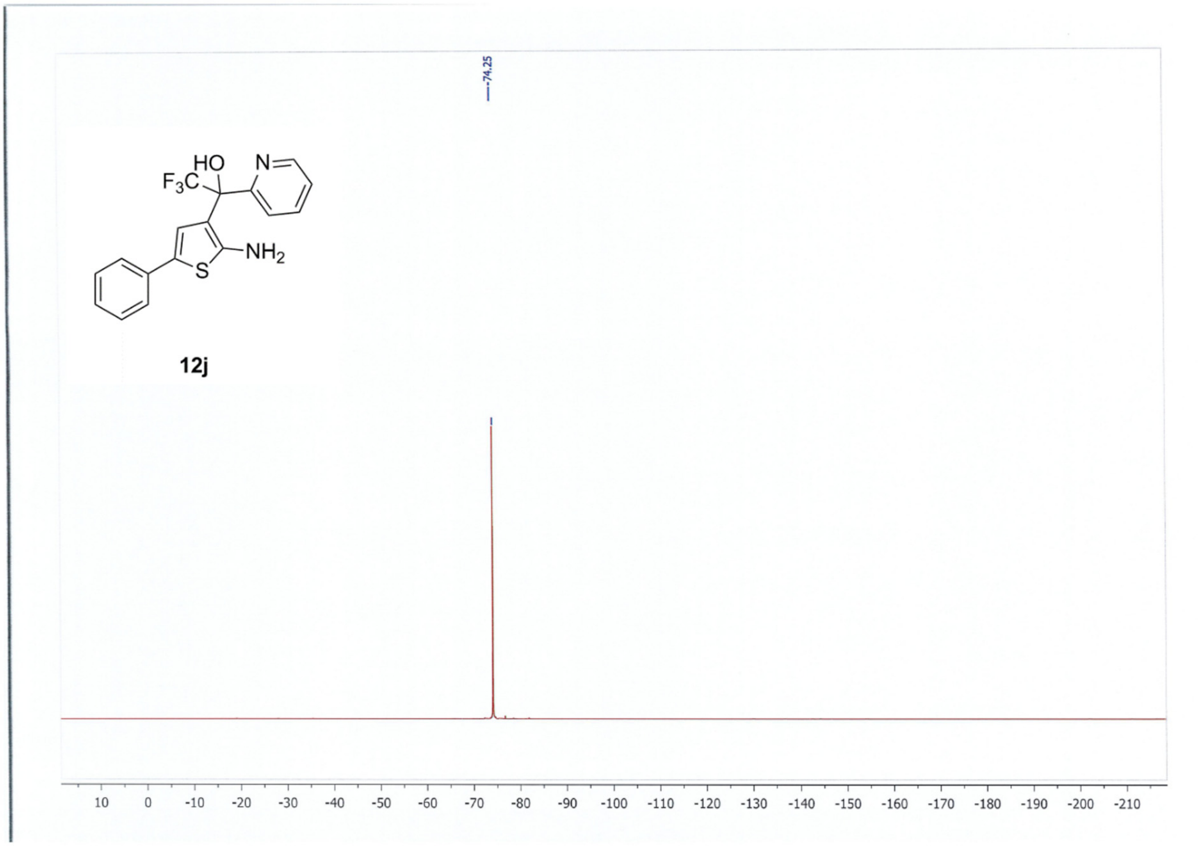
-74.25
HO
N
F_3C
NH_2
S
12j
10 0 -10 -20 -30 -40 -50 -60 -70 -80 -90 -100 -110 -120 -130 -140 -150 -160 -170 -180 -190 -200 -210

Elemental Composition Report

Page 1

Single Mass Analysis

Tolerance = 3.0 mDa / DBE: min = -1.0, max = 100.0
Element prediction: Off
Number of isotope peaks used for i-FIT = 3

Monoisotopic Mass, Even Electron Ions
282 formula(e) evaluated with 2 results within limits (up to 50 best isotopic matches for each mass)
Elements Used:
C: 0-100 H: 0-100 N: 0-10 O: 0-10 F: 3-3 S: 1-1

SYNAPT G2-S#NotSet
CHR-2_1 12 (0.211) Cm (10:14)

VD-MRH-53

02-Feb-2021
1: TOF MS ES+
2.98e+005

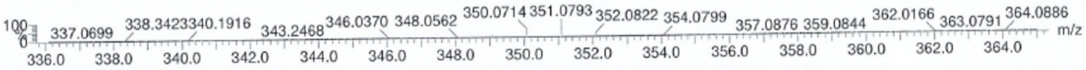

Minimum:				-1.0				
Maximum:		3.0	3.0	100.0				
Mass	Calc. Mass	mDa	PPM	DBE	i-FIT	Norm	Conf(%)	Formula
351.0793	351.0779	1.4	4.0	10.5	2095.4	0.000	100.00	C17 H14 N2 O F3 S
	351.0811	-1.8	-5.1	2.5	2105.3	9.919	0.00	C6 H14 N8 O4 F3 S

VD-MRH-53

2: Diode Array
Range: 2.633e+1

Time	Height	Area	Area%
19.73	383387	187656.61	1.99
28.97	246459	53874.64	0.57
30.30	236333	66559.60	0.71
31.37	22722778	9121802.00	96.73

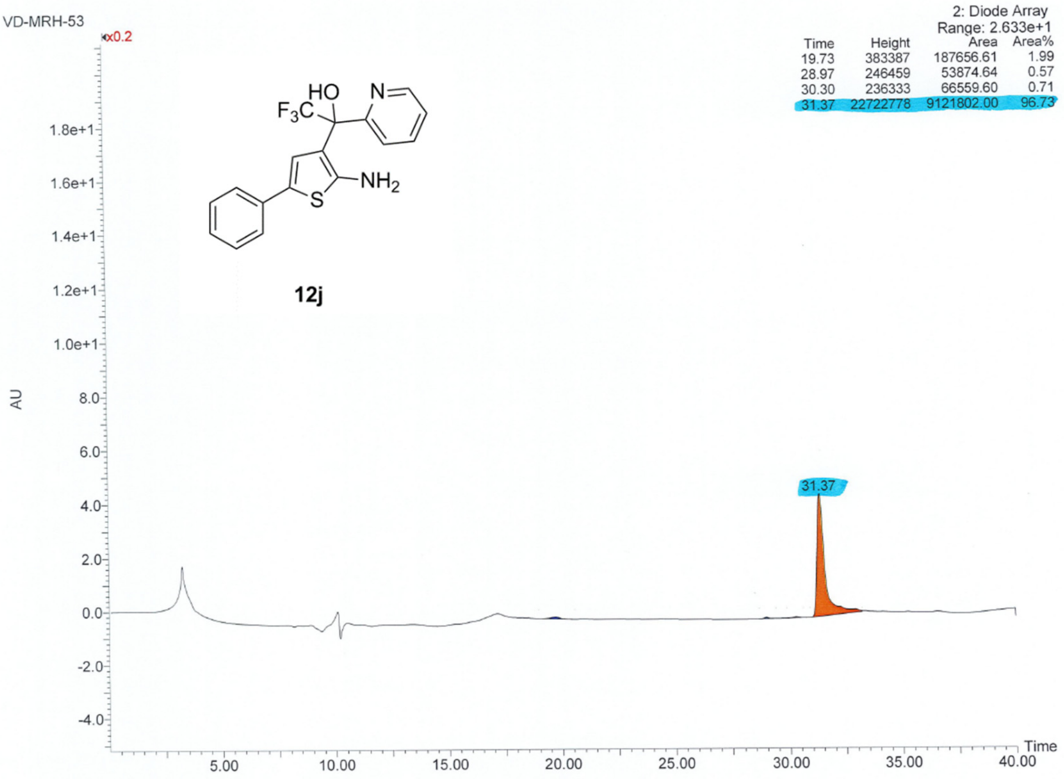

*1-(2-amino-5-phenylthiophen-3-yl)-1-(benzo[d]thiazol-2-yl)-2,2,2-trifluoroethan-1-ol (**12k**)*

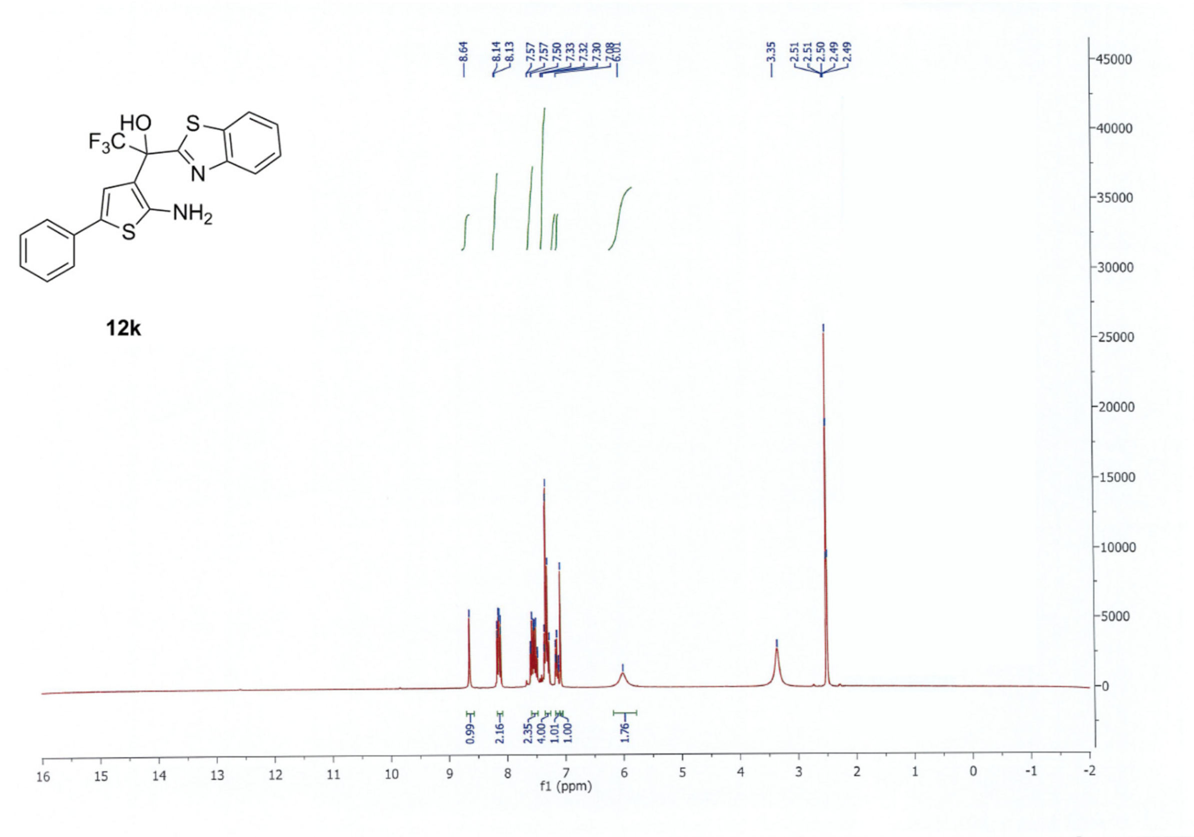

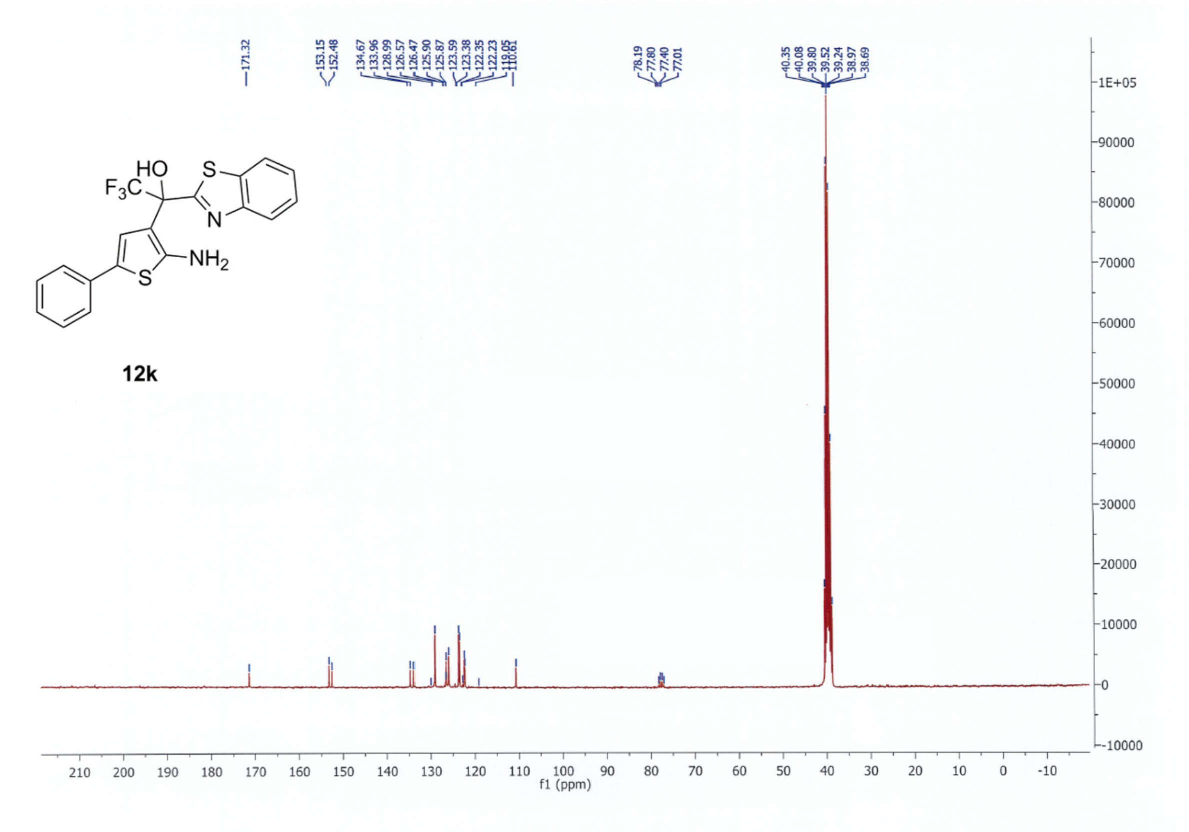

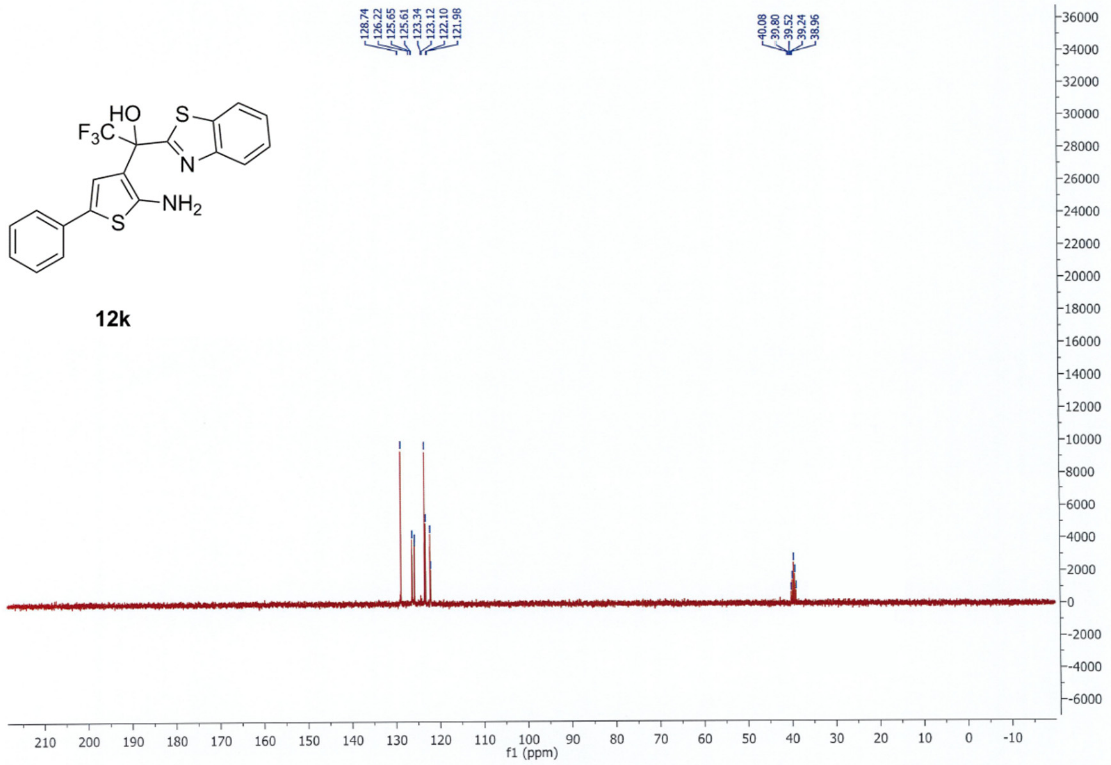
128.74
126.22
125.65
125.61
123.34
123.12
122.10
121.98
40.08
39.80
39.52
39.24
38.96
12k
f1 (ppm)

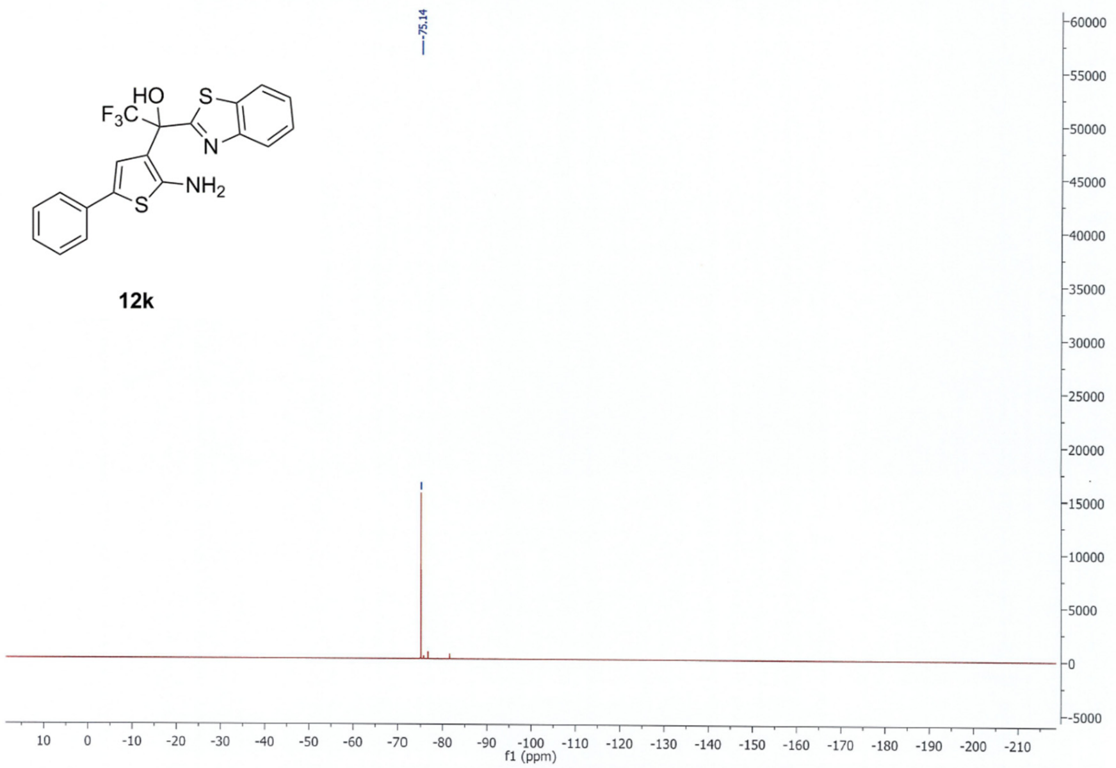
-75.14
12k
f1 (ppm)

High Resolution Mass Result

**Analysis Info**		Acquisition Date	7/23/2021 6:47:26 PM
Sample Name	**DUV-43 _ VD-MRH-105**	Instrument / Ser#	micrOTOF-Q 228888.10300

Acquisition Parameter

Source Type	ESI	Ion Polarity	Positive	Scan Begin	50 m/z	Scan End	2200 m/z

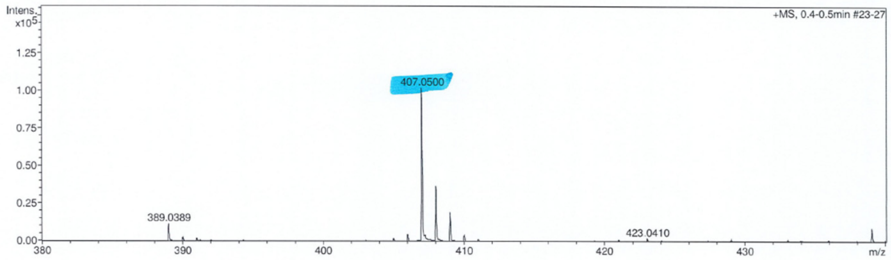

Meas. m/z	#	Ion Formula	m/z	err [ppm]	mSigma	# Sigma	Score	rdb	e⁻ Conf	N-Rule
407.0500	1	C19H14F3N2OS2	407.0494	1.5	66.7	1	100.00	12.5	even	ok
	2	C16H15F4N2O2S2	407.0506	1.3	80.4	2	61.23	8.5	even	ok

Bruker Compass DataAnalysis 4.1 printed: 7/23/2021 6:55:07 PM Page 1 of 1

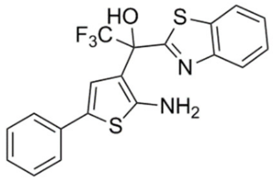

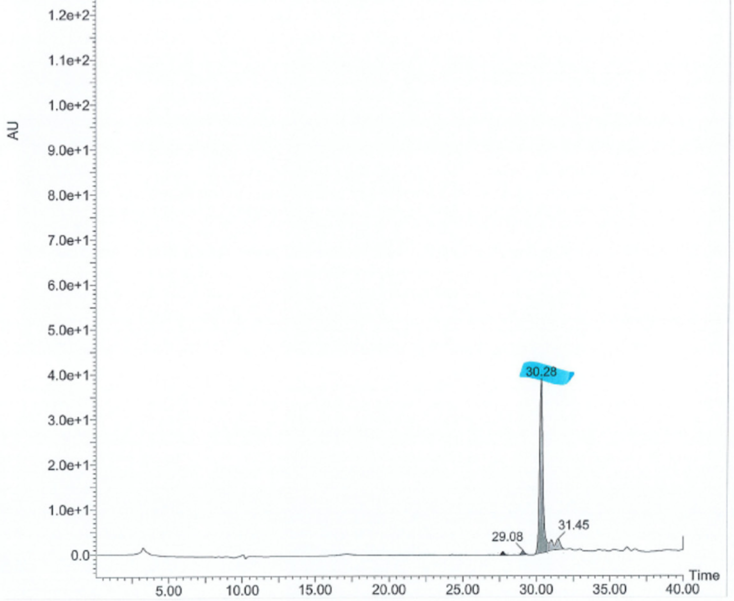

2: Diode Array
Range: 1.987e+2

Time	Height	Area	Area%
27.72	3990655	908670.88	1.53
29.08	4350372	1001353.38	1.68
30.28	193750160	50047920.00	84.18
31.45	11536283	7495846.00	12.61

12k

*1-(2-amino-5-phenylthiophen-3-yl)-2,2,2-trifluoro-1-(naphthalen-1-yl)ethan-1-ol (**12l**)*

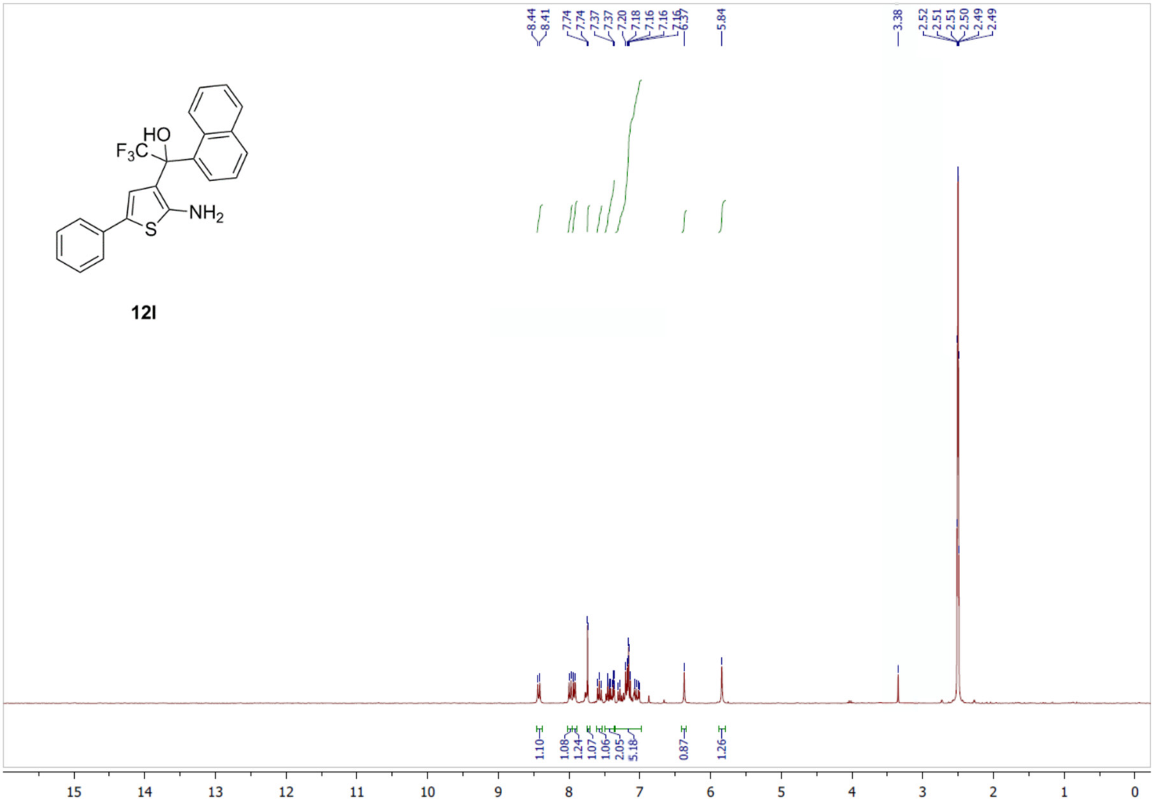

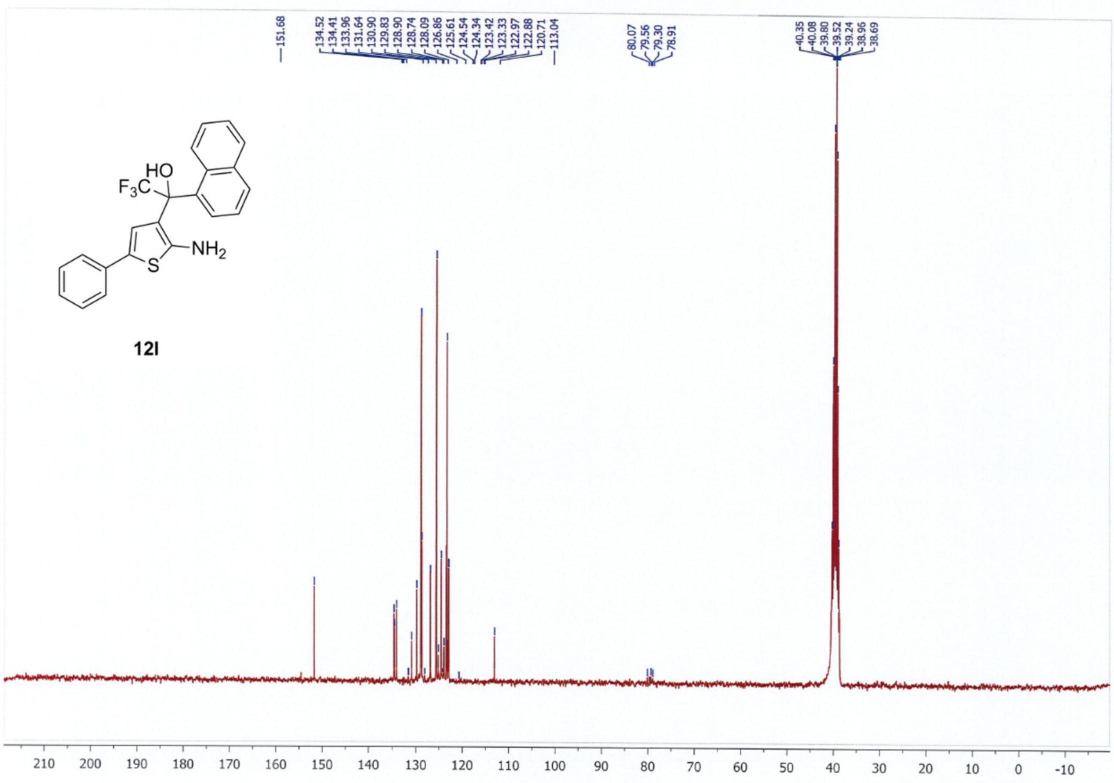

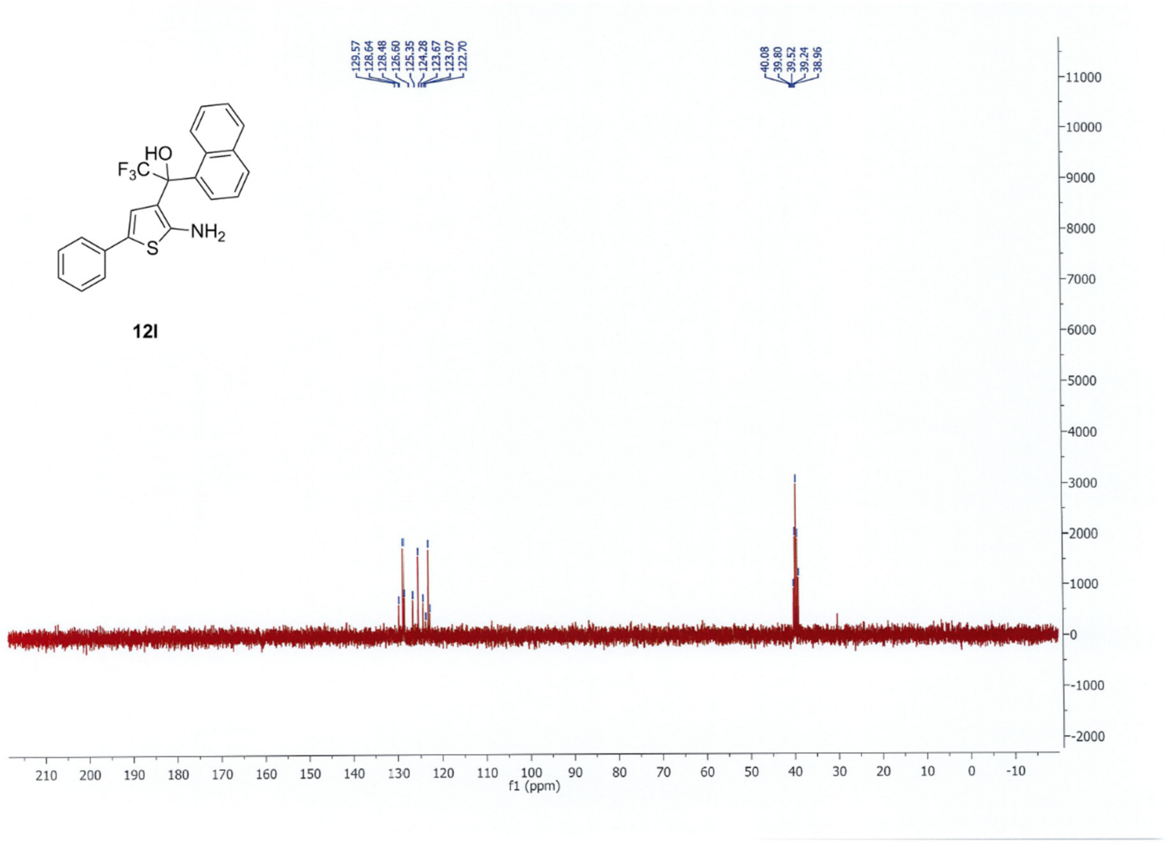
129.57
128.64
128.48
126.60
125.35
124.28
123.67
123.07
122.70
40.08
39.80
39.52
39.24
38.96
HO
F3C
NH2
S
12l
f1 (ppm)

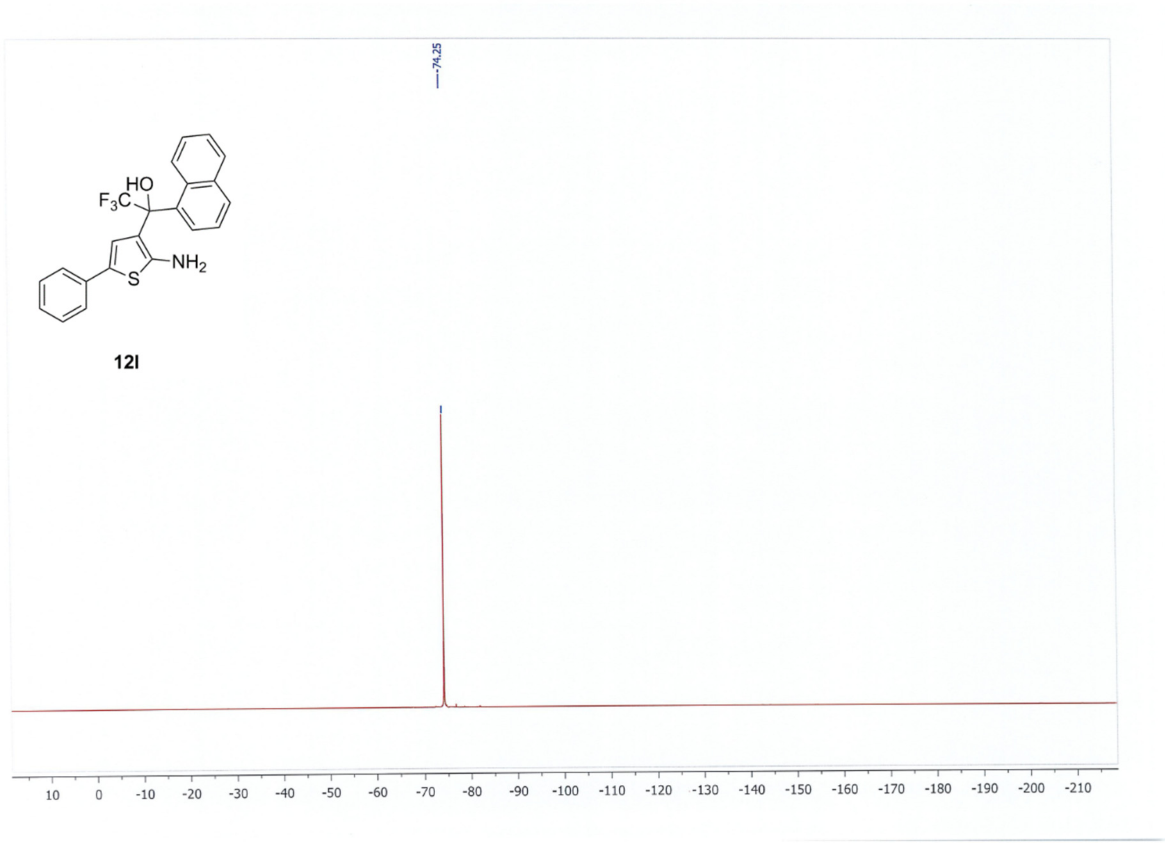
-74.25
HO
F3C
NH2
S
12l

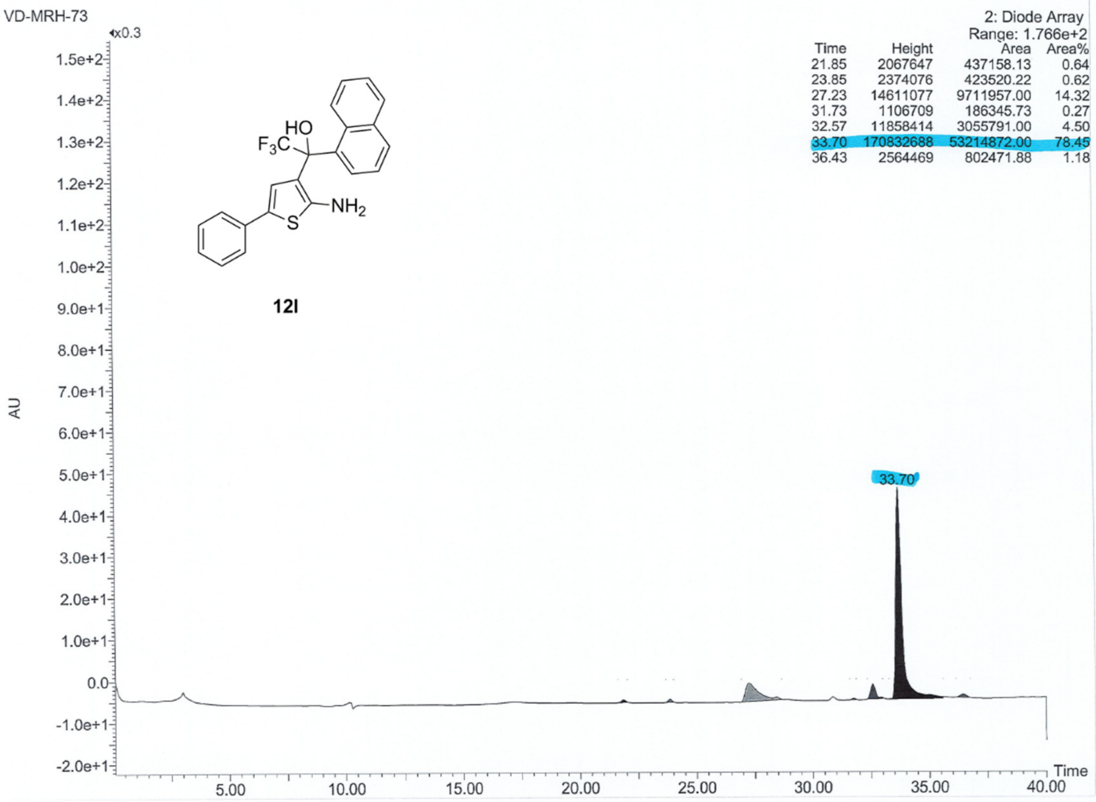

2: Diode Array
Range: 1.766e+2

Time	Height	Area	Area%
21.85	2067647	437158.13	0.64
23.85	2374076	423520.22	0.62
27.23	14611077	9711957.00	14.32
31.73	1106709	186345.73	0.27
32.57	11858414	3055791.00	4.50
33.70	170832688	53214872.00	78.45
36.43	2564469	802471.88	1.18

High Resolution Mass Result

Analysis Info

Sample Name **VD-MRH-73**

Acquisition Date 5/10/2021 2:52:18 PM

Instrument / Ser# micrOTOF-Q 228888.10300

Acquisition Parameter

Source Type ESI | Ion Polarity Positive | Scan Begin 50 m/z | Scan End 2200 m/z

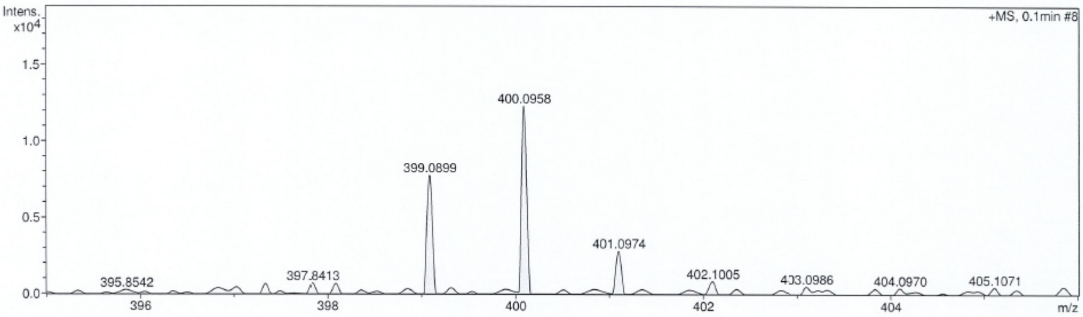

Meas. m/z	#	Ion Formula	m/z	err [ppm]	mSigma	# Sigma	Score	rdb	e⁻ Conf	N-Rule
382.0874	1	C22H15F3NS	382.0872	-0.7	15.9	1	100.00	14.5	even	ok
400.0958	1	C22H17F3NOS	400.0977	4.9	18.7	1	100.00	13.5	even	ok
432.0871	1	C22H17F3NO3S	432.0876	1.1	13.5	1	100.00	13.5	even	ok
	2	C18H13F3N7OS	432.0849	-5.1	23.2	2	26.92	14.5	even	ok
